# Vegetarian Dietary Patterns and Cardiometabolic Risk in People With or at High Risk of Cardiovascular Disease: A Systematic Review and Meta-analysis

**DOI:** 10.1001/jamanetworkopen.2023.25658

**Published:** 2023-07-25

**Authors:** Tian Wang, Cynthia M. Kroeger, Sophie Cassidy, Sayan Mitra, Rosilene V. Ribeiro, Shane Jose, Andrius Masedunskas, Alistair M. Senior, Luigi Fontana

**Affiliations:** 1 Charles Perkins Centre, Faculty of Medicine and Health School of Life and Environmental Sciences, School of Mathematics and Statistics, University of Sydney, Sydney, New South Wales, Australia; 2 Department of Endocrinology, Royal Prince Alfred Hospital, Sydney, New South Wales, Australia; 3 Department of Clinical and Experimental Sciences, Brescia University, Brescia, Italy

## Abstract

**IMPORTANCE:**

Plant-based diets are known to improve cardiometabolic risk in the general population, but their effects on people at high risk of cardiovascular diseases (CVDs) remain inconclusive.

**OBJECTIVE:**

To assess the association of vegetarian diets with major cardiometabolic risk factors, including low-density lipoprotein cholesterol (LDL-C), hemoglobin A_1c_ (HbA_1c_), systolic blood pressure (SBP), and body weight in people with or at high risk of CVDs.

**DATA SOURCES:**

This meta-analysis was registered before the study was conducted. Systematic searches performed included Embase, MEDLINE, CINAHL, and CENTRAL from inception until July 31, 2021.

**STUDY SELECTION:**

Eligible randomized clinical trials (RCTs) that delivered vegetarian diets in adults with or at high risk of CVDs and measured LDL-C, HbA_1c_ or SBP were included. Of the 7871 records screened, 29 (0.4%; 20 studies) met inclusion criteria.

**DATA EXTRACTION AND SYNTHESIS:**

Two reviewers independently extracted data including demographics, study design, sample size, and diet description, and performed risk of bias assessment. A random-effects model was used to assess mean changes in LDL-C, HbA_1c_, SBP, and body weight. The overall certainty of evidence was evaluated using the Grading of Recommendations, Assessment, Development, and Evaluation (GRADE) tool.

**MAIN OUTCOMES AND MEASURES:**

Mean differences between groups in changes (preintervention vs postintervention) of LDL-C, HbA_1c_, and SBP; secondary outcomes were changes in body weight and energy intake.

**RESULTS:**

Twenty RCTs involving 1878 participants (range of mean age, 28-64 years) were included, and mean duration of intervention was 25.4 weeks (range, 2 to 24 months). Four studies targeted people with CVDs, 7 focused on diabetes, and 9 included people with at least 2 CVD risk factors. Overall, relative to all comparison diets, meta-analyses showed that consuming vegetarian diets for an average of 6 months was associated with decreased LDL-C, HbA_1c_, and body weight by 6.6 mg/dL (95% CI, −10.1 to −3.1), 0.24% (95% CI, −0.40 to −0.07), and 3.4 kg (95% CI, −4.9 to −2.0), respectively, but the association with SBP was not significant (−0.1 mm Hg; 95% CI, −2.8 to 2.6). The GRADE assessment showed a moderate level of evidence for LDL-C and HbA_1c_ reduction.

**CONCLUSIONS AND RELEVANCE:**

In this study, consuming a vegetarian diet was associated with significant improvements in LDL-C, HbA_1c_ and body weight beyond standard therapy in individuals at high risk of CVDs. Additional high-quality trials are warranted to further elucidate the effects of healthy plant-based diets in people with CVDs.

## Key Points

**Question:**

Do vegetarian diets improve the cardiometabolic profile of people with or at high risk of cardiovascular diseases (CVDs)?

**Findings:**

In this meta-analysis of 20 randomized clinical trials (with 1878 participants) with an average 6 months of intervention, vegetarian diets were associated with significant improvements in low-density lipoprotein cholesterol by 6.8 mg/dL, hemoglobin A_1c_ by 0.25%, and body weight by 3.4 kg. The GRADE assessment showed a moderate level of evidence for low-density lipoprotein cholesterol and hemoglobin A_1c_ reduction.

**Meaning:**

These results suggest that consuming a vegetarian diet may modestly but significantly improve cardiometabolic outcomes beyond standard pharmacological therapy in individuals at high risk of CVDs, highlighting the potential protective and synergistic effects of vegetarian diets for the primary prevention of CVD.

## Teaser

This systematic review and meta-analysis examines 20 clinical trials of various vegetarian diets for improved outcomes for patients with major cardiometabolic risk factors.

## Introduction

Despite major therapeutic advancements, cardiovascular diseases (CVDs) remain the leading cause of disease burden and escalating health care costs worldwide. Much of this is attributable to the failed implementation of prevention strategies to comprehensively address modifiable risk factors in individuals at risk.^1^ In fact, researchers found that most US residents who experienced a myocardial infarction had at least 1 suboptimal CVD risk factor before the event.^2,3^ Thus, identifying any practical intervention that can improve cardiometabolic profiles beyond standard therapy in high-risk individuals is critical in CVD prevention and should be one of the main focuses of clinicians, health practitioners, and researchers.

Poor diet is mechanistically linked to an elevated risk of CVD morbidity and mortality.^4^ The 2021 dietary guidelines of the American Heart Association emphasize the importance of dietary patterns rich in minimally processed plant foods, fish and seafood, and low-fat dairy products.^5^ Different forms of vegetarian diets, which exclude meat and sometimes also eggs and dairy, are becoming increasingly popular because of their health and environmental benefits.^6^ Diabetes organizations recommend the consumption of well-balanced vegetarian diets in line with the vast evidence supporting their beneficial effects in preventing type 2 diabetes and some of its metabolic complications.^7,8,9^

Accumulating data from meta-analyses of randomized clinical trials (RCTs) suggest a role of vegetarian diets in the primary prevention of CVDs in the general population,^10,11^ but little is known on their effectiveness in patients with or at high risk of CVDs. Moreover, metabolic outcomes among different vegetarian diets (eg, vegan vs lacto-ovo-vegetarian diets) were not investigated thoroughly,^10,11,12,13,14^ with little control for key confounders, such as energy restriction,^12,15,16^ physical activity,^12,15^ and medication changes.^10,12^

To the best of our knowledge, no meta-analysis of RCTs has been conducted to investigate the association of vegetarian diets with outcomes among people with CVD—indeed, research here has primarily focused on observational studies.^17,18^ For example, Glenn et al^17^ combined evidence from 7 cohort studies and found that a vegetarian diet was not associated with CVD mortality, with the evidence graded as very low due to indirectness and imprecision. An observational study found that individuals may choose to follow vegetarian diets because of perceived health benefits; these individuals may also present fewer adverse health behaviors (eg, smoking, excessive alcohol intake), and the findings can be affected by these confounders.^18^ In general, findings from observational studies provide less strength of evidence, as they cannot rule out residual confounding, and a causal relationship cannot be established.^14,17,18,19^ Thus, our meta-analysis aims to fill in this gap, with subgroup analyses controlling for energy restriction, physical activity, medication use, and the type of control diet.

## Methods

This systematic review and meta-analysis was registered with PROSPERO (CRD42021218348) before the study was conducted. Institutional review board approval and informed consent were not required as this was a secondary analysis of deidentified data. We followed the Preferred Reporting Items for Systematic Reviews and Meta-analyses (PRISMA) reporting guideline and the AMSTAR-2 checklist^20^ for this study.

### Search Strategy and Selection Criteria

Search strategy was informed by PICOS criteria (Population, Intervention, Comparator, Outcome, Study design) (eTable 1 in Supplement 1). We performed systematic searches in Embase, MEDLINE, CINAHL and CENTRAL (Cochrane Central Register of Controlled Trials), from inception until July 31, 2021 (eTable 2 in Supplement 1). Hand searches of reference lists of reviews, protocols, abstracts, and gray literature (eg, websites mentioning relevant studies) were performed to supplement searches. The authors of the ongoing trials and abstracts were contacted at least 3 times to retrieve preliminary findings and full manuscripts.

Eligible RCTs delivered vegetarian diets in adults with or at high risk of CVDs and measured low-density lipoprotein cholesterol (LDL-C), hemoglobin A_1c_ (HbA_1c_), or systolic blood pressure (SBP) were included. Of the 7871 records screened, 29 (0.4%; 20 studies) met inclusion criteria. Two reviewers (T. W. and one of C. K., S. C., A. M., S. M., or R. R.) independently extracted data including demographics, study design, sample size, and diet description, and performed risk of bias assessment.

### Statistical Analysis

The primary outcomes are the mean differences between groups in changes (preintervention vs postintervention) in LDL-C, HbA_1c_, and SBP. The secondary outcomes are changes in body weight, and energy intake. The meta package of R version 1.4.1717 (R Project for Statistical Computing)^21^ was used to perform meta-analysis and meta-regression.^22^ A random-effects model was used, implemented using the metacont function for mean differences. We estimated the overall pooled effect size based on inverse-variance weighting using a restricted maximum likelihood estimator for the among-study heterogeneity.^23^ Confidence intervals are at the 95% level and estimated based on a standard-normal distribution (ie, default method in the meta package). The total heterogeneity was quantified as τ^2^ (ie, variance among effect sizes not attributable to sampling). The statistical significance for heterogeneity was assessed by a modified *Q* test, which used the Farebrother method to obtain the distribution of *Q* values, as recommended for mean differences by Kulinskaya et al.^24^ We evaluated the overall certainty of evidence using the Grading of Recommendations, Assessment, Development, and Evaluation (GRADE) tool.

## Results

### Characteristics of Included Studies

**Figure 1.**
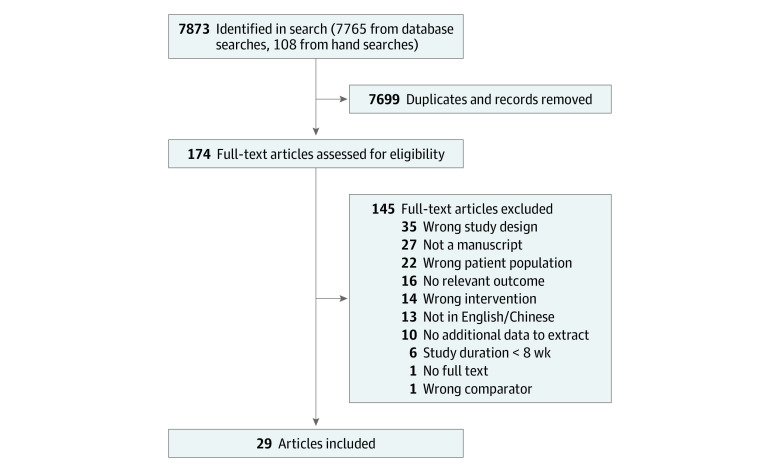
Flow Diagram of Study Selection

Of the 7871 records screened, 29 articles (20 RCTs; 1878 total participants) were included (Figure 1). Reasons for exclusion at the full-text stage are presented in Figure 1 and eTable 3 in Supplement 1. Seven ongoing trials were identified (eTable 4 in Supplement 1); however, preliminary results were not yet available. RCTs were mostly parallel-group trials, except for 2 crossover design studies (Table).^31,49^ Studies were conducted in the US,^25,26,30,31,32,33,38,41,42,44,45,47,51,52^ Asia,^36,39,40^ Europe,^37,49^ or New Zealand,^53^ and all published between 1990 and 2021. Sample size ranged from 13 to 291 participants (mean age, 28 to 64 years), and mean duration of intervention was 25.4 weeks (range, 2-24 months).

**Table.** Study Characteristics of Included Randomized Clinical Trials

Source	Country	Study groups (No. randomized)	Participants (intervention/control)			Diet description	Study length and design
			Population condition	Gender	Age, mean (SD), y		
Aldana et al,^25^ 2006	US	Dr Ornish Program (46)	CHD	47.8% male	60.9 (9.7)	Very LF, LOV diet (no animal proteins except for nonfat dairy and egg whites); recommend liberal consumption of fruits and vegetables, whole grains, and legumes; daily serving of soy food	1 y, RCT, parallel group
		Traditional cardiac rehabilitation (47)		64.6% male	62.2 (8.9)	No dietary intervention (traditional cardiac rehabilitation)	
Barnard et al,^26,27,28,29^ 2006	US	LF vegan diet (49)	Type 2 diabetes and being overweight (BMI ≥25)	27 female; 22 male	56.7 (range, 35-82)	No animal products and added fats; fruits and vegetables, grains, and legumes, favor low-GI foods; vitamin B12 supplement (100 μg) every other day	22 wk, RCT, parallel group; 52 wk follow-up
		ADA diet (50)		33 female; 17 male	54.6 (range, 27-80)	Participants with a BMI >25 were prescribed energy deficits of 500-1000 kcal; vitamin B12 supplement (100 μg) every other day	
Barnard et al,^30^ 2018	US	LF vegan diet (22)	Type 2 diabetes (HbA_1c_, 6.5%-10.5%) and being overweight (BMI ≥25)	13 female; 9 male	62 (range, 41-79)	Whole grains, fruits and vegetables, legumes; no animal products and added oils; no restrictions on energy or carbohydrate intake	20 wk RCT, parallel group
		Portion-controlled eating plan (23)		11 female; 12 male	61 (range, 30-75)	Energy limits when needed for weight loss (calorie restricted −500 kcal/d)) and guidance on portion sizes	
Barnard et al, 2021^31^	US	LF vegan diet first (31)	Overweight (BMI, 28-40) and high LDL cholesterol (>100 mg/dL)	22 female; 8 male	58.3 (8.4)	Grains, fruits and vegetables, legumes; no animal products and added oils; daily vitamin B12 supplement (500 mcg) during the vegan phase	16 wk, randomized, crossover trial; 4 wk washout period
		Mediterranean diet first (31)		26 female; 6 male	56.6 (10.9)	Select white meats (with visible fat removed) instead of red meats; use extra virgin olive oil instead of other fats or oils; daily food servings: vegetables ≥2, fresh fruits ≥2-3; weekly food servings: legumes ≥3, fish or shellfish ≥3, nuts or seeds ≥3	
Bunner et al,^32^ 2015	US	LF vegan diet + B12 supplement (17)	Type 2 diabetes with painful diabetic neuropathy for ≥6 mo	11 female; 6 male	57 (6)	No animal products; focused on grains, fruits and vegetables, legumes; limited fat intake to 20-30 g/d; favored low-GI foods; daily vitamin B12 supplement (1000 mcg)	20 wk randomized, parallel group, clinical trial
		No intervention + B12 supplement (17)		8 female; 9 male	58 (6)	No dietary change except for daily vitamin B12 supplement (1000 mcg)	
Burke et al,^33,34,35^ 2006	US	SBT + LOV (participant preferred, 36; participant not preferred, 48)	Overweight (BMI, 27-43) and high LDL cholesterol (>100 mg/dL)	73 female (86.9)	45.0 (8.2)	No animal flesh foods; restrict consumption of calories (1200-1500 for women and 1500-1800 for men) and fat (25% of total calories)	12 mo, randomized, parallel group, clinical trial; 6 mo follow up
		SBT (participant preferred, 63^a^; participant not preferred, 50)		86 female (87.8)	43.4 (8.9)	SBT: restrict calories (1200-1500 for women and 1500-1800 for men) and fat (25% of total calories)	
Garousi et al, 2021^36^	Iran	LOV (40)	Overweight (BMI ≥25) and high LDL cholesterol (>100 mg/dL)	13 male; 13 female	43.51 (9.85)	Included protein sources from egg (24%), dairy (19%), gluten (26%), soy (16%), nuts (8%), vegetables, and fruits (7%); no animal flesh foods; calorie restricted (−500 kcal/d)	3 mo (12 wk), randomized, parallel group, clinical trial
		Standard weight loss diet (40)		14 male; 12 female	42.84 (9.85)	Approximately 18% of protein sources from meat and meat products, poultry, fish and seafood, and flesh of any other animal; calorie restricted (−500 kcal/d)	
Kahleova et al,^37^ 2010	Czech Republic	Lacto-vegetarian diet (37)	Type 2 diabetes (HbA_1c_, 6%-11%) and being overweight (BMI, 25-53)	20 female; 17 male	54.6 (7.8)	Grains, fruits and vegetables, legumes; animal products limited to ≤1 portion of LF yogurt/d; vegetarian meals provided in 2 vegetarian restaurants; calorie-restricted (−500 kcal/d); daily vitamin B12 supplement (50 μg).	24 wk, randomized, open, parallel clinical trial; second 12 wk diet were combined with aerobic exercise
		Conventional diabetic diet (37)		19 female; 18 male	57.7 (4.9)	Following dietary guidelines of the Diabetes and Nutrition Study Group of the European Association for the Study of Diabetes; meals were provided; calorie-restricted (−500 kcal/d); daily vitamin B12 supplement (50 μg)	
Kahleova et al,^38^ 2020	US	LF vegan diet (122)	Overweight (BMI, 28-40) and high LDL cholesterol (>100 mg/dL)	105 female; 17 male	53 (10)	No animal products or added fats; daily vitamin B12 supplement (500 μg); consisted of fruits and vegetables, grains, legumes	16 wk, RCT using a single-center, open parallel design
		No dietary change (122)		106 female; 16 male	57 (13)	No dietary changes	
Lee et al,^39^ 2016	Korea	Vegan diet + brown rice (53)	Type 2 diabetes (HbA_1c_ level, 6.0%-10.0%)	40 female; 6 male	57.5 (7.7) [32–70]	Fruits and vegetables, whole grains, legumes; no animal products, polished rice (white rice) and processed food made of rice or wheat flour; have unpolished rice (brown rice)	3 mo (12 wk), RCT, parallel group
		Conventional diabetic diet (53)		35 female; 12 male	58.3 (7.0) [range, 40-69]	Followed 2011 treatment guidelines by the KDA; restrict individualized daily energy intake based on body weight, physical activity, need for weight control, and compliance	
Liao et al,^40^ 2007	Republic of China	Soy low-calorie diet (15)	Overweight (BMI, >26) and high LDL cholesterol (>100 mg/dL)	3 male; 12 female	28.8 (9.1)	Soy protein as the only protein source; provided various soy foods, including drinks, miso, tofu, and vegetarian meat substitutes from markets; 1200 kcal/d	8 wk, RCT, parallel group
		Traditional low-calorie diet (15)		3 male; 12 female	38.0 (11.1)	Two-thirds of total protein consumed was animal protein; 1200 kcal/d	
Mahon et al,^41^ 2007	US	LOV plus carbohydrates	Overweight (BMI >25) and high LDL cholesterol (>100 mg/dL)	61 female	58 (2)	1250 kcal/d-1000 kcal/d LOV diet and provided 250 kcal/d portioned nonmeat carbohydrate (shortbread cookies and sugar-coated chocolates)	11-wk protocol: a 2-wk weight maintenance period, followed by a 9-wk period of dietary intervention and energy restriction. RCT, parallel group
		LOV plus beef					
		LOV plus chicken					
		Control				Habitual diet	
Mishra et al,^42,43^ 2013	US	LF vegan diet (142)	Overweight (BMI >25) and/or previous diagnosis of type 2 diabetes	110 female; 32 male	44.3 (15.3)	No animal products; minimize added oils; vitamin B12 supplement/d; consisted of fruits and vegetables, whole grains, legumes; no energy restriction; LF vegan menu options made available	18 wk cluster RCT, parallel group
		Usual diet (149)		132 female; 18 male	46.1 (13.6)	No dietary changes; no dietary guidance	
Nicholson et al,^44^ 1999	US	LF vegan diet (7)	Noninsulin-dependent diabetes	3 female; 4 male	51 (range, 34-62)	No animal products, added oils and refined carbohydrates; consisted of fruits and vegetables, whole grains, legumes; the diet was adequate in all nutrients except vitamin B12	12 wk RCT, parallel group
		Conventional LF diet (6)		2 female; 2 male	60 (range, 51-74)	Emphasized fish and poultry, rather than red meat	
Ornish et al,^45,46^ 1990	US	LF LOV diet (53)	CHD	1 female; 21 male	56.1 (7.5)	Fruits and vegetables, grains, legumes and soybean products; no animal proteins except for nonfat dairy and egg whites; vitamin B12 supplemented	Initially 1 y RCT, but extended the study for an additional 4 y, parallel group
		Usual care (40)		4 female; 15 male	59.8 (9.1)	No lifestyle changes	
Shah et al,^47,48^ 2018	US	Vegan diet (50)	CHD; >3/4 with dyslipidemia; >1/2 with hypertension	57 female; 43 male	Median (IQR), 63.0 (57.0-68.0)	The vegan diet: whole-food plant-based diet with no processed foods; no animal products; vitamin B12 fortified soy milk; given vegan cookbook	8 wk randomized, open-label, masked clinical trial, parallel group
		AHA diet (50)		58 female; 42 male	Median (IQR), 59.5 (53.0-67.0)	Given AHA LF, low-cholesterol cookbook	
Sofi et al,^49,50^ 2018	Italy	LOV (60)	Overweight (BMI ≥25) and presence of ≥1 of: TC >190 mg/dL; LDL >115 mg/dL, TG >150 mg/dL, glucose levels 110-126 mg/dL	49 female; 11 male	Median, 49.5 (range, 24-70)	No animal products except for eggs and dairy products; included all the other food groups; hypocaloric with respect to the energy requirements of the participants, but completely isocaloric with the Mediterranean diet	6-mo randomized, open, crossover trial with no washout period.
		Mediterranean diet (58)		43 female; 15 male	Median, 52 (range, 21-75)	Consumption of all food groups, including animal flesh foods; hypocaloric with respect to the energy requirements of the participants	3 mo for each intervention.
Tang et al,^51^ 2013	US	Normal protein: the lacto-vegetarian diet	Overweight (BMI 25.0-39.9) and high LDL cholesterol (>100 mg/dL)	45 total participants (all male)	44.8 (3.6); Median, 43 (range, 24- 75)	No animal flesh foods and egg products; portioned quantities of milk comprising 13% of total protein intake; 0.8 g protein/kg/d	12 wk RCT, parallel group
		High protein: omnivorous			51.0 (2.6); Median, 52 (24-69)	Portioned quantities of cooked lean pork and egg products comprising 40% of total protein intake (25% from pork, 15% from eggs); 1.4 g protein kg/d	
Toobert et al,^52^ 2000	US	Prime Time program: very LF, LOV diet (17)	CHD	Female	64 (10)	No animal products other than egg whites and nonfat yogurt; no added oils or other concentrated fats; <10% calories from fat	2 y RCT, parallel group
		Usual care (11)			63 (11)	Usual care	
Wright et al,^53^ 2017	New Zealand	Whole food plant-based diet + normal care (33)	Overweight (BMI ≥25) with a diagnosis of type 2 diabetes, CHD, orhypertension, or hypercholesterolemia	22 female; 11 male	56 (9.9)	Fruits and vegetables, whole grains, legumes; no animal products and refined oil; discouraged high-fat plant foods (eg, nuts and avocados), and highly processed foods	6-mo 2-group, parallel RCT
		Normal care (32)		17 female; 15 male	56 (9.5)	Normal care	

Abbreviations: ADA, American Diabetes Associations; AHA, American Heart Associations; BMI, body mass index (calculated as weight in kilograms divided by height in meters squared); CHD, coronary heart disease; GI, glycemic index; HbA_1c_, hemoglobin A_1c_; KDA, Korean Diabetes Association; LDL, low-density lipoprotein cholesterol; LF, low-fat; LOV, lacto-ovo-vegetarian diet; RCT, randomized clinical trial; SBT, standard calorie- and fat-restricted weight loss diet; TC, total cholesterol; TG, triglyceride.

To convert glucose to millimoles per liter, multiply by 0.0555; HbA_1c_ to proportion of total hemoglobin, 0.01; LDL cholesterol to millimoles per liter, 0.0259; total cholesterol to millimoles per liter, 0.0259.

^a^ Results for 15 participants discarded.

Of included trials, 4 targeted people with CVDs^25,45,47,52^ of which 3 applied the Ornish diet.^25,45,52^ This diet is a very low-fat (less than 10% energy from fat), lacto-ovo-vegetarian diet that excludes all animal proteins except for nonfat dairy products and egg whites (Table). Seven studies^26,30,32,37,39,42,44^ focused on individuals with type 2 diabetes, and most^26,30,32,39,42,44^ delivered a low-fat, vegan diet. This diet excludes all animal products, with vitamin B12 supplemented in some studies^26,32,42,44^ to balance nutrient intake. Different vegetarian diets were delivered in individuals with at least 2 risk factors for CVDs, including vegan diets,^31,38,40,53^ lacto-ovo-vegetarian diets^33,36,41,49^ and a lacto-vegetarian diet.^51^ Energy restrictions were prescribed in several studies^33,36,40,41,49,51^ to promote weight loss. Overall, the most commonly prescribed diets were vegan diets,^26,30,31,32,38,39,40,42,44,47,53^ followed by lacto-ovo-vegetarian diets,^25,33,36,41,45,49,52^ and lacto-vegetarian diets.^37,51^

### Effects of Vegetarian Diets on LDL-C, HbA_1c_, SBP, Weight, Energy Intake, and Medication Use

**Figure 2.**
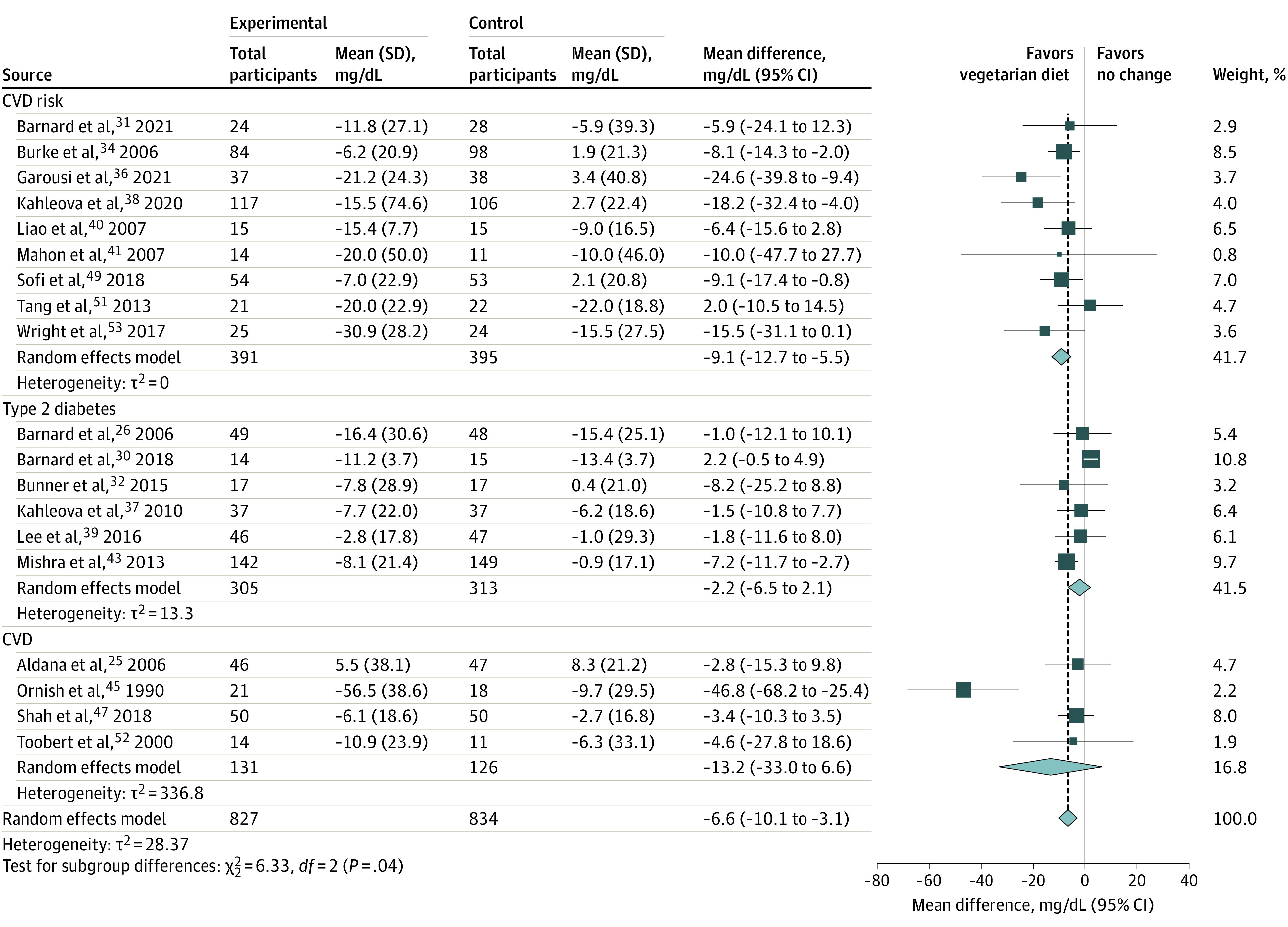
Random Effects Model Meta-Analysis for Changes in Low-Density Lipoprotein-Cholesterol Concentrations Comparing Vegetarian Diets Intervention and All Comparison Diets, Grouped by Disease Status of Participants CVD indicates cardiovascular disease.

Nineteen studies (1661 participants; trial duration, 8 weeks to 2 years)^25,26,30,31,32,33,36,37,38,39,40,41,42,45,47,49,51,52,53^ were included in the meta-analysis of LDL-C (Figure 2; eFigures 1-14 in Supplement 1). Compared with control diets, consuming a vegetarian diet was associated with significantly decreased LDL-C by 6.6 mg/dL (95% CI, −10.1 to −3.1 mg/dL) in a mean of 6 months of intervention beyond that achieved with standard therapy (to convert LDL-C to millimoles per liter, multiply by 0.0259). However, a moderate but statistically significant among-study heterogeneity was noted (*Q* = 20788.2; *P* = .04). We therefore conducted a sensitivity analysis excluding Ornish et al^45^ and the effect size remained statistically significant (−5.4 mg/dL; 95% CI, −8.4 to −2.3 mg/dL) (eFigure 1 in Supplement 1). Baseline LDL-C levels were associated with responsiveness to dietary intervention, with greater reductions detected in studies with higher baseline values. Baseline values explained 100% of among-study heterogeneity (*P* < .001) (eFigure 15 in Supplement 1). The most consistent reduction was observed in people at high risk of CVDs (−9.1 mg/dL; 95% CI, −12.7 to −5.5 mg/dL) (Figure 2). Among all different vegetarian diets, lacto-ovo vegetarian diets were associated with the greatest reduction in LDL-C (−14.1 mg/dL; 95% CI, −24.5 to −3.6 mg/dL) (eFigure 2 in Supplement 1); however, 4 out of 5 trials^33,36,41,49^ restricted energy intake. Compared with usual diet, vegetarian diets lowered LDL-C by 12.9 mg/dL (95% CI, −21.4 to −4.5 mg/dL), and this reduction is clinically significant (eFigure 3 in Supplement 1). However, the association of vegetarian diets with LDL-C levels was not significant when compared with active controls (eg, diabetic diet). Vegetarian diets were associated with similar LDL-C reduction in studies with (−7.2 mg/dL; 95% CI, −10.8 to −3.5 mg/dL) and without energy restriction (−6.8 mg/dL; 95% CI, −12.1 to −1.6 mg/dL) (eFigure 4 in Supplement 1). Furthermore, vegetarian diets were associated with lowered LDL-C (−5.9 mg/dL; 95% CI, −10.1 to −1.7 mg/dL) in studies with no physical activity intervention (eFigure 5 in Supplement 1). The association of vegetarian diets with LDL-C was similar among individuals with (−6.1 mg/dL; 95% CI, −10.3 to −2.0 mg/dL) and without (−6.2 mg/dL; 95% CI, −11.0 to −1.4 mg/dL) changes of lipid-lowering medication dosage (eFigure 6 in Supplement 1). Finally, a greater decrease in LDL-C was observed in trials reporting only data of completers (−11.3 mg/dL; 95% CI, −17.0 to −5.6 mg/dL) compared with those that used the intention-to-treat analysis (−5.1 mg/dL; 95% CI, −8.1 to −2.2 mg/dL) (eFigure 7 in Supplement 1). Funnel plot of LDL-C was symmetrical indicating low risk of publication bias (eFigure 16 in Supplement 1).

**Figure 3.**
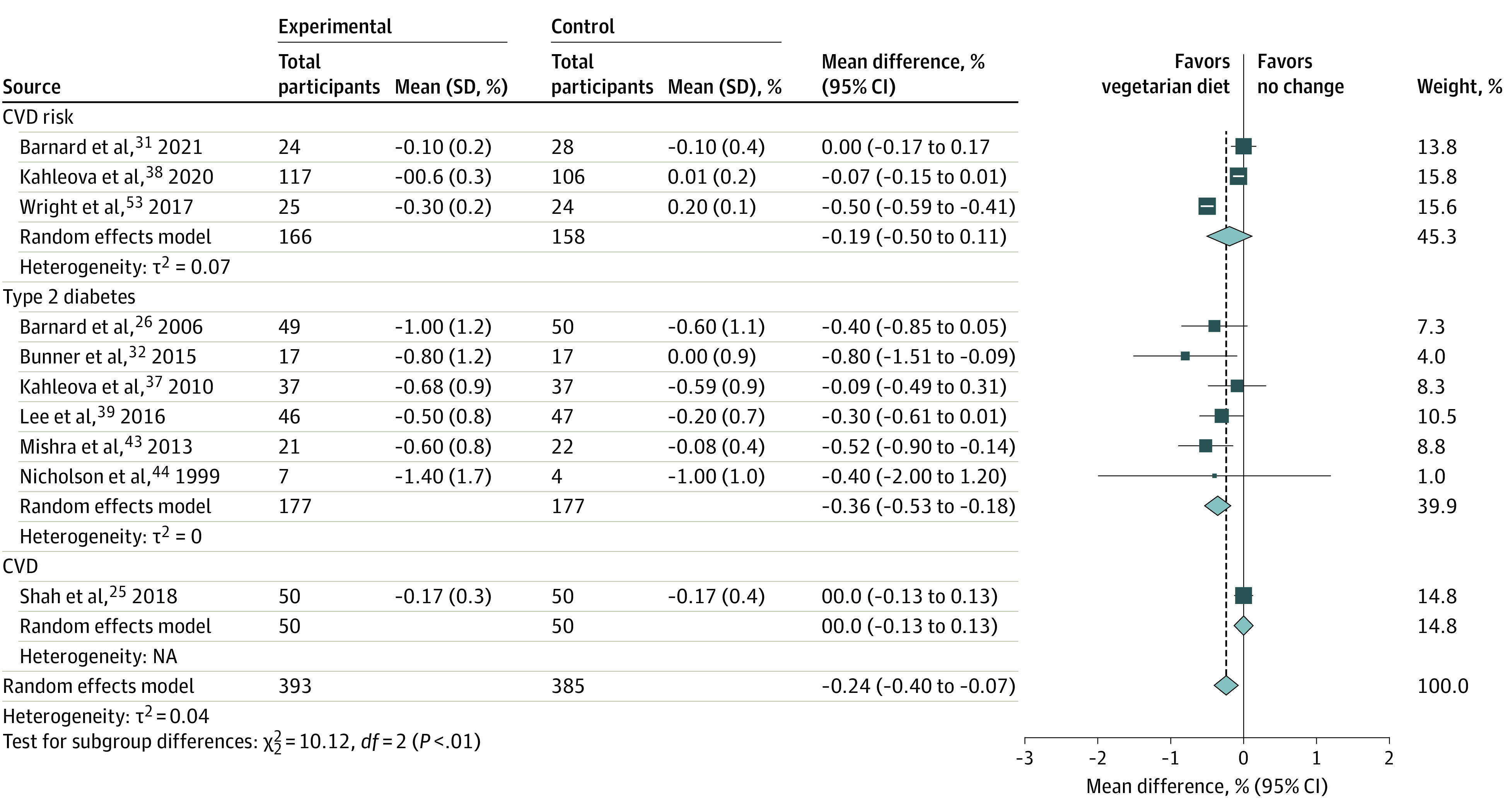
Random Effects Model Meta-Analysis for Changes in Hemoglobin A_1c_ Comparing Vegetarian Diets Intervention and All Comparison Diets, Grouped by Disease Status of Participants CVD indicates cardiovascular disease; NA, not applicable.

Ten studies (778 participants; trial duration, 8 weeks to 6 months)^26,31,32,37,38,39,42,44,47,53^ were included in the HbA_1c_ meta-analysis (Figure 3, eFigures 17-29 in Supplement 1). Overall, consuming vegetarian diets was associated with decreased HbA_1c_ by 0.24% (95% CI, −0.40 to −0.07) in a mean 6 months of intervention (to convert HbA_1c_ to proportion of total hemoglobin, multiply by 0.01), and the heterogeneity was not statistically significant (*Q* = 9.22; *P* = .15), with greater effect observed in studies of people with type 2 diabetes (−0.36%; 95% CI, −0.53 to −0.18) (Figure 3; eFigure 30 in Supplement 1). A reduction in HbA_1c_ (−0.26%; 95% CI, −0.44 to −0.08) was observed in people following a vegan diet^26,31,32,38,39,42,44,47,53^ even without energy restriction (eFigures 17 and 18 in Supplement 1). Improvements in HbA_1c_ were also observed when vegetarian diets were compared with both usual (−0.39%; 95% CI, −0.69 to −0.10) and the conventional energy-restricted diabetic diet (−0.26%; 95% CI, −0.48 to −0.05) (eFigure 19 in Supplement 1).^26,37,39^ Furthermore, vegetarian diets were associated with improved HbA_1c_ (−0.21%; 95% CI, −0.38 to −0.05) in studies with no physical activity prescription (eFigure 20 in Supplement 1). Finally, the improvement in HbA_1c_ remained significant (−0.27%; 95% CI, −0.49 to −0.05) in studies that used the intention-to-treat analysis (eFigure 21 in Supplement 1). Funnel plot of HbA_1c_ were symmetrical suggesting low risk of publication bias (eFigure 31 in Supplement 1).

**Figure 4.**
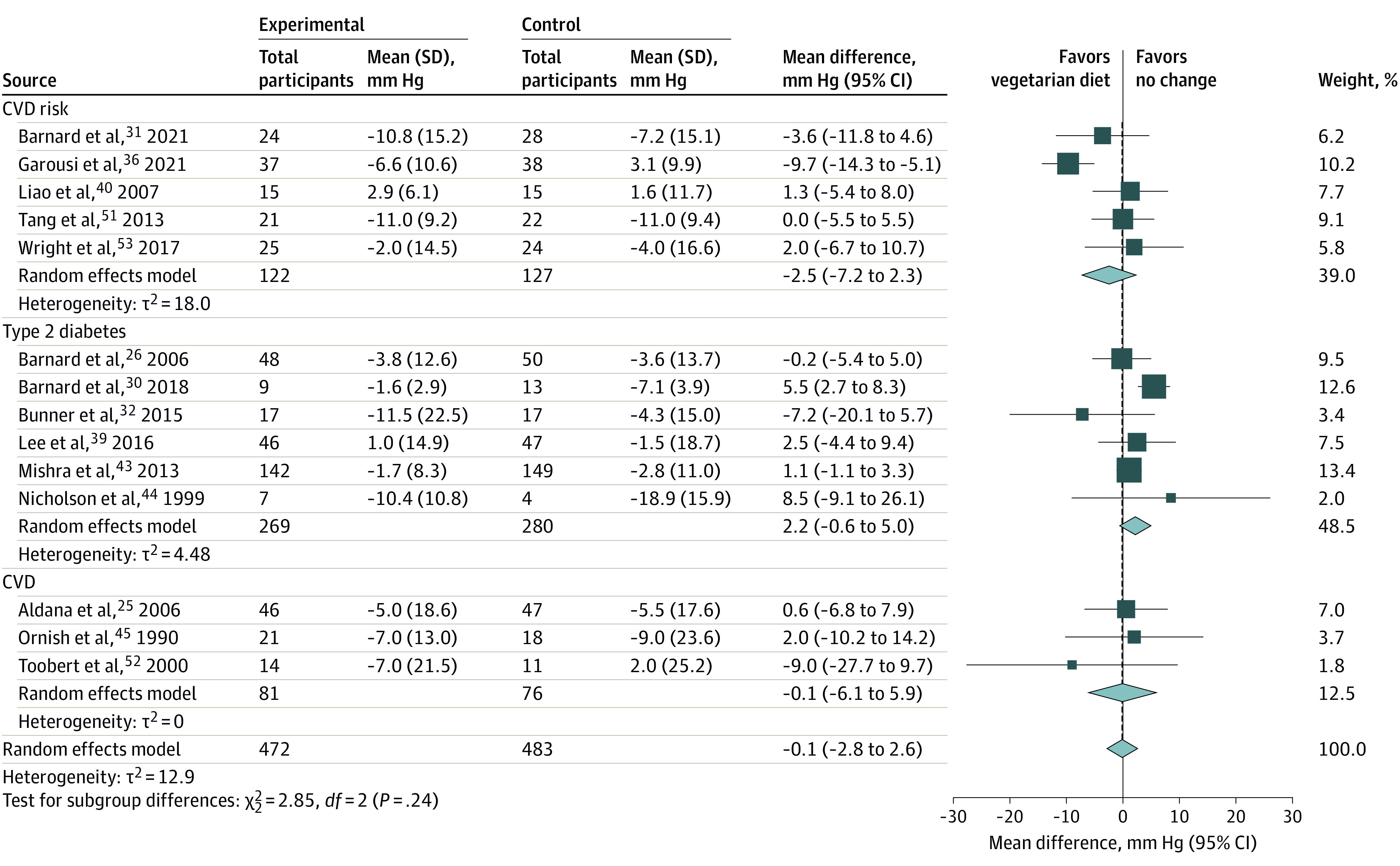
Random Effects Model Meta-Analysis for Changes in Systolic Blood Pressure Comparing Vegetarian Diets Intervention and All Comparison Diets, Grouped by Disease Status of Participants CVD indicates cardiovascular disease.

Fourteen studies (955 participants; trial duration, 8 weeks to 2 years)^25,26,30,31,32,36,39,40,42,44,45,51,52,53^ were included in the meta-analysis of SBP, and the pooled effect size of vegetarian diets was not statistically significant (−0.1 mm Hg; 95% CI, −2.8 to 2.6 mm Hg) (Figure 4; eFigures 32-44 in Supplement 1). Among-study heterogeneity was not statistically significant (*Q* = 3520.37, *P* = .29) (eFigure 45 in Supplement 1). Funnel plot of SBP was symmetrical, indicating low risk of publication bias (eFigure 46 in Supplement 1).

Sixteen RCTs (1395 participants; trial duration, 8 weeks to 1 year)^25,26,30,31,32,33,36,38,40,41,42,44,45,49,51,53^ were included in the meta-analysis of body weight (eFigures 47-52 in Supplement 1). Overall, body weight decreased by 3.4 kg in a mean 6 months of intervention (95% CI, −4.9 to −2.0 kg) in individuals randomized to vegetarian diets (eFigure 47 in Supplement 1). Among-study heterogeneity was very high (*Q* = 2795.31, *df* = 15, *P* < .001) (eFigure 53 in Supplement 1). The greatest reduction was observed in people at high risk of CVD (−3.6 kg; 95% CI, −5.8 to −1.4 kg), followed by people with type 2 diabetes (−2.8 kg; 95% CI, −4.2 to −1.4 kg). Paradoxically, a greater reduction was observed in interventions without energy restriction (−4.7 kg; 95% CI, −6.6 to −2.7 kg) when compared with energy-restricted vegetarian diets (−1.8 kg; 95% CI, −3.3 to −0.2 kg) (eFigure 48 in Supplement 1). However, it is important to note that 6 out of 10 studies^25,32,38,42,45,53^ of vegetarian diets without energy restriction used usual diet as the comparison group; whereas in 5 out of 6 trials^33,36,40,49,51^ of energy-restricted vegetarian diets, omnivorous energy-restricted interventional diet (eg, diabetic diet) were the comparison group. This was consistent with our results that showed how vegetarian diets were associated with greater weight reduction compared with usual diets (−5.5 kg; 95% CI, −7.7 to −3.2 kg) than with energy-restricted interventional diet (−1.4 kg; 95% CI, −2.4 to −0.4 kg) (eFigure 49 in Supplement 1). Funnel plot of weight was symmetrical indicating low risk of publication bias (eFigure 54 in Supplement 1).

Finally, individuals on energy-unrestricted vegetarian diets^38,42,45,52^ significantly reduced energy intake compared with the usual diet (−275.7 kcal; 95% CI, −376.5 to −175.0 kcal) (eFigures 55 and 56 in Supplement 1). Funnel plot of energy intake was symmetrical, suggesting low risk of publication bias (eFigure 57 in Supplement 1).

It should be noted that most participants included in the studies (14 total studies)^25,26,30,31,32,33,37,38,39,42,44,47,52,53^ were taking medication for management of suboptimal cardiometabolic profiles at enrollment (eTable 5 in Supplement 1). Eight RCTs^26,30,31,32,33,37,44,53^ found a reduction in medication dose for hyperglycemia, dyslipidemia, and/or hypertension, although their improvements in LDL-C and HbA_1c_ did not reach clinical significance. In contrast, 2 RCTs^36,49^ excluded patients on medications that could influence cardiometabolic outcomes; these studies significantly improved SBP and LDL-C.

### Diet Quality and Adherence of Trials

Thirteen studies^25,26,30,31,32,37,38,39,42,44,45,47,53^ emphasized the consumption of plant-based whole foods (Table). eTable 6 in Supplement 1 shows comprehensive dietary data of all included trials. Most studies^26,30,31,33,36,37,38,39,40,41,42,44,45,47,49,52,53^ used a 3-day food record or validated 24-hour recall to collect dietary data. However, key macronutrients intake was not assessed thoroughly, and this limits the capacity to evaluate diet quality and adherence. More than one-third of included studies^25,32,33,38,40,41,51,52,53^ did not report saturated fat intake and/or total cholesterol intake; nearly half of the trials^25,30,32,33,45,49,51,52,53^ did not report dietary fiber intake; only 2 studies^26,47^ reported trans fatty acid intake; finally, only 1 trial^31^ reported alcohol intake. In most of the trials that carefully measured these macronutrients, a significant improvement in saturated fat, total cholesterol, and dietary fiber intake was found.

### Risk of Bias Assessment and GRADE Quality Rating

eFigure 58 in Supplement 1 shows the risk of bias assessment (the detailed descriptions can be found in eTable 7 in Supplement 1). Only 8 trials^26,30,31,37,38^ clearly described the randomization process; 12 studies^25,30,31,36,37,39,41,44,47,49,51,52^ failed to present any deviations from the intended intervention; 6 studies^25,31,36,41,44,52^ raised bias concern in missing outcome data and all studies performed well in terms of outcome measurement. Indeed, our primary outcomes were objective measurements that are unlikely to be influenced by assessors’ knowledge of group allocation. Finally, 11 studies^25,30,31,32,33,37,41,44,47,49,51^ selectively reported outcomes. Overall, only 3 studies^26,38,53^ rated as having a low risk of bias, and the majority^25,30,31,33,36,37,41,44,47,49,51,52^ rated as having a high risk of bias.

eTables 8-11 in Supplement 1 show the GRADE assessment for outcomes by different populations. We did not rate down studies for lack of masking because it is impossible to mask participants in nutritional trials targeting whole dietary patterns. Overall, the level of evidence was rated moderate for LDL-C and HbA_1c_ reduction, and low for SBP and weight reduction (eTable 12 in Supplement 1). However, a high level of evidence for LDL-C reduction was found in people at high risk of CVD, with the mean difference close to clinical significance (cut point of 10 mg/dL). In contrast, in this group, the evidence of reduction in HbA_1c_, SBP, and weight was rated very low due to inconsistency (high heterogeneity) and imprecision (sample size under 400 and mean difference includes zero). In individuals with type 2 diabetes, the certainty of the evidence was rated moderate for a reduction in HbA_1c_ and an increase in SBP. Note that 4 of 6 studies^26,30,39,44^ included in the GRADE assessment of SBP used active control as the comparison diet. We found low evidence for LDL-C and weight reduction in people with type 2 diabetes. Finally, in people with CVD, the certainty of the evidence was low in LDL-C and SBP reduction. The outcomes and funding sources of included studies are described in eTable 13 and eTable 14 in Supplement 1.

## Discussion

Findings from pharmacological randomized trials of statins, antidiabetic, and antihypertensive drugs have clearly shown that lowering cholesterol, glucose, and blood pressure levels exerts major antiatherosclerotic and nephro-protective effects.^54,55^ The results of this meta-analysis demonstrate that consuming a vegetarian diet exerts a modest but significant effect in concomitantly reducing multiple key risk factors, including LDL-C, HbA_1c_, and body weight, especially in high-risk patients. In population-stratified analysis, the greatest reduction in LDL-C was observed in individuals at high risk of CVD. Vegetarian diets were most effective in glycemic control among people with type 2 diabetes, and led to favorable changes in weight in people at high risk of CVD and in those with type 2 diabetes, suggesting that vegetarian diets might have a synergistic (or at least nonantagonistic) use in potentiating the effects of optimal drug therapy in the prevention and treatment of a range of cardiometabolic diseases.

To the best of our knowledge, this meta-analysis is the first that generates evidence from RCTs to assess the association of vegetarian diets with outcomes in people affected by CVDs. Previous meta-analyses of RCTs reported a favorable association of vegetarian diets with LDL-C (−12.2 mg/dL; 95% CI, −17.7 to −6.7 mg/dL),^56^ HbA_1c_ (−0.29%; 95% CI, −0.45 to −0.12%),^16^ SBP (−2.5 mm Hg; 95% CI, −3.6 to −1.4 mm Hg),^12^ and body weight (−2.2 kg; 95% CI, −2.8 to −1.2 kg),^10^ which is consistent with our findings in terms of LDL-C, HbA_1c_, and body weight improvement. However, most prior meta-analyses did not stratify populations by disease status,^10,56^ type of vegetarian diet,^16^ nor comparison diet.^10,12,16,56^ Moreover, important confounders such as energy restriction,^12,15,16,56^ physical activity,^12,15,16,56^ or changes in medication use^10,12,16^ were not controlled for. For instance, Yoko et al^56^ reported a greater improvement in LDL-C, but 7 out of the 17 studies targeted general population, and 5 studies had a duration of less than 8 weeks. To fill in this research gap, our meta-analysis only included mid- to long-term trials (ie, longer than 8 weeks) of patients with or at high risk of CVD.

Interestingly, we did not observe a significant change in SBP, consistent with the findings of previous meta-analyses,^57,58^ suggesting that diet quality plays a major role in lowering blood pressure, independent of animal food consumption, as the DASH diet trial demonstrated.^59^ Different from the general population, most patients included in our study^25,26,30,31,32,33,37,38,39,42,44,47,52,53^ took medications to manage their hypertension, hyperglycemia, and/or dyslipidemia at trial enrollment. Eight RCTs^26,30,31,32,33,37,44,53^ reported a decrease in medication dose due to intervention effect. Although our results show an overall improvement in LDL-C, HbA_1c_, and weight, independent of energy restriction, the changes in LDL-C and HbA_1c_ did not reach the clinically significance as per cutoff target. In fact, the use of glucose, lipid, and blood pressure-lowering drugs, and the reduction in medication dosage, may obscure the favorable effect on cardiometabolic outcomes induced by vegetarian diets, implying a larger actual effect size. This hypothesis is supported by 2 RCTs^36,49^ in our meta-analysis that required patients not to take medication that could influence cardiometabolic outcomes; these studies significantly improved SBP and LDL-C.

### Potential Mechanisms

There are different forms of vegetarian diets: pesco-vegetarian diets eliminate animal products except for fish and seafood; lacto-ovo-vegetarians exclude meat and fish but not dairy products and eggs; vegans eliminate all animal food including honey. Well-balanced and adequately supplemented vegetarian (and vegan) diets can have multiple health benefits, including lower intake of: saturated fat,^60^ L-carnitine and choline (precursors of the atherogenic TMAO),^61^ and branch chain amino acids (promoters of insulin resistance^62,63^ and platelet activation via tropomodulin-3 propionylation).^64^ In our meta-analysis, 12 studies emphasized low-fat content, which may in part contribute to the observed improvement in LDL-C. Depending on their design, these diets may also be high in dietary fiber, sterols, mono- and polyunsaturated fatty acids, potassium, magnesium, phytochemicals, and have lower energy density and lower scores on the glycemic index.^7,65,66^ Indeed, not all vegetarian diets could be considered healthy. For instance, more than one-third of the studies included in our meta-analysis did not emphasize the importance of consuming minimally processed plant-based whole foods. Vegetarian diets, particularly those practiced for ethical reasons or focused on convenience, may contain high levels of so-called empty calories, refined carbohydrates, hydrogenated oils, high-fructose corn syrup, sucrose or artificial sweeteners and salt. We were unable to perform a thorough evaluation of diet quality and adherence for the studies included in our meta-analysis because of limited dietary data (eTable 6 in Supplement 1). Therefore, it is possible that some diets were of poor quality, which could explain the modest reductions in body weight and HbA_1c_, and no significant change in SBP. Furthermore, consumption of plant-based diets that emphasize energy-dense high–glycemic index refined carbohydrates, deep-fried foods rich in trans fatty acids and salty take-away meals are associated with a 32% higher risk of coronary heart disease^67^ as well as high risk of type 2 diabetes.^68^

### Strengths and Limitations

The strengths of this study included: (1) to our knowledge, this is the first meta-analysis generating evidence of vegetarian diets from RCTs in people with CVDs; (2) we strictly followed a prespecified protocol; (3) we conducted a rigorous search using 4 databases, including trials registries, supplemented by hand searches; (4) we performed comprehensive subgroup analyses to examine people with different disease status and the effect of different vegetarian diets, control diets, energy restriction, physical activity, medication, and analyses method; (5) we contacted authors of included studies to extract unpublished data, and to improve the accuracy of meta-analysis calculations; (6) we used the GRADE approach to assess the overall certainty of the evidence for each cardiometabolic outcome in people with different disease status; and (7) we published the R script for meta-analysis and shared the data set, hence our findings are transparent and reproducible (eAppendix in Supplement 2).

This article had several limitations. Only 4 trials (each of them with 100 participants or less) investigated vegetarian diets in patients with CVDs, of which 3 required SD imputation as these could not be obtained from authors. Second, although we conducted comprehensive subgroup analyses, the results could be limited by the relatively small sample sizes (200 participants or less) of the subgroups as reflected by the GRADE assessment. Furthermore, these subgroup analyses did not reach the required sample size to draw solid conclusions, and some of them had high heterogeneity in the pooled estimate. Third, we were not able to assess adherence and diet quality thoroughly due to limited dietary data. Future research should fully document dietary data including energy, macronutrients, micronutrients, and ideally food groups and other relevant information (eg, cooking methods) to allow for a thorough assessment of adherence and diet quality. Fourth, many of the studies included in this meta-analysis emphasized a low-fat diet, and data on the effectiveness of vegetarianism combined with higher or moderate fat intake on cardiometabolic health are limited. Fifth, most of included studies were conducted in Western countries (17 studies) and mostly in the US (14 studies), which could limit the generalizability of our findings to other populations. Past research revealed that the quality of vegetarian diets varied between South Asian and American with vegetarians in the US consistently consuming healthier food groups than South Asian vegetarians.^69^ Thus, our findings should be cautiously interpreted when applied to other populations. Sixth, most included studies had a trial duration between 8 weeks to 6 months, and there is a lack of studies investigating long-term adherence (ie, beyond 6 months) to vegetarian diets on cardiometabolic health in individuals at high risk of CVD. Seventh, although the detected publication bias was low and we conducted a rigorous search, we cannot rule out the possibility of missing unpublished data. Eighth, only studies published in English and Chinese were included, and trials published in other languages were likely to have been missed (eTable 12 in Supplement 1).

## Conclusions

In summary, our findings contribute important information to the development of clinical guidelines in cardiometabolic risk factors management as they demonstrate that consuming a vegetarian diet was associated with significant reductions in LDL-C, HbA_1c_ and body weight, beyond standard therapy, in patients with or at high risk of CVDs. The greatest improvements in HbA_1c_ and LDL-C were observed in individuals with type 2 diabetes and people at high risk of CVD, highlighting the potential protective and synergistic effects of vegetarian diets for the primary prevention of CVD. Well-designed nutrition clinical trials with comprehensive dietary information are warranted to investigate the full effect of high-quality vegetarian diets in combination with optimal pharmacological therapy in people with CVDs.

## Notes

Supplement 1.
eMethods.
**eTable 1.** Description of PICOS Criteria **eTable 2.** Complete Search Strategies for Different Databases **eTable 3.** Inclusion and Exclusion Criteria of Included Studies **eTable 4.** Study Characteristics of Ongoing Clinical Trials That Meet Our Eligibility Criteria **eTable 5.** Participant Characteristics and Description of Included Studies **eTable 6.** Dietary Data of Included Studies **eTable 7.** Detailed Risk of Bias Assessment of Included Studies **eTable 8.** GRADE Assessment in People With CVD or People at High Risk of CVD **eTable 9.** GRADE Assessment in People at High Risk of CVD **eTable 10.** GRADE Assessment in People With T2DM **eTable 11.** GRADE Assessment in People With CVD **eTable 12.** Studies Excluded at Stage 2 With Reasons **eTable 13.** Reported Conflicts of Interest (COI) and Funding Sources of Included Studies **eTable 14.** Primary and Secondary Outcomes of Included Studies **eFigure 1.** The Forest Plot for Changes in LDL-C, Grouped by Disease Status Excluding Ornish’s Study **eFigure 2.** The Forest Plot for Changes in LDL-C, Grouped by Vegetarian Diets **eFigure 3.** The Forest Plot for Changes in LDL-C, Grouped by Control Diets **eFigure 4.** The Forest Plot for Changes in LDL-C, Grouped by Energy Restriction **eFigure 5.** The Forest Plot for Changes in LDL-C, Grouped by Physical Activity **eFigure 6.** The Forest Plot for Changes in LDL-C, Grouped by Medication Use **eFigure 7.** The Forest Plot for Changes in LDL-C, Grouped by Analysis Method **eFigure 8.** The Forest Plot for Changes in LDL-C, Grouped by Disease Status Excluding Imputed Data **eFigure 9.** The Forest Plot for Changes in LDL-C, Grouped by Vegetarian Diets Excluding Imputed Data **eFigure 10.** The Forest Plot for Changes in LDL-C, Grouped by Control Diets Excluding Imputed Data **eFigure 11.** The Forest Plot for Changes in LDL-C, Grouped by Energy Restriction Excluding Imputed Data **eFigure 12.** The Forest Plot for Changes in LDL-C, Grouped by Physical Activity Excluding Imputed Data **eFigure 13.** The Forest Plot for Changes in LDL-C, Grouped by Medication Use Excluding Imputed Data **eFigure 14.** The Forest Plot for Changes in LDL-C, Grouped by Analysis Method Excluding Imputed Data **eFigure 15.** Treatment Effect of LDL-C Across Baseline LDL-C **eFigure 16.** Funnel Plot of LDL-C **eFigure 17.** The Forest Plot for Changes in HbA1c, Grouped by Vegetarian Diets **eFigure 18.** The Forest Plot for Changes in HbA1c, Grouped by Control Diets **eFigure 19.** The Forest Plot for Changes in HbA1c, Grouped by Energy Restriction **eFigure 20.** The Forest Plot for Changes in HbA1c, Grouped by Physical Activity **eFigure 21.** The Forest Plot for Changes in HbA1c, Grouped by Medication Use **eFigure 22.** The Forest Plot for Changes in HbA1c, Grouped by Analysis Method **eFigure 23.** The Forest Plot for Changes in HbA1c, Grouped by Disease Status Excluding Imputed Data **eFigure 24.** The Forest Plot for Changes in HbA1c, Grouped by Vegetarian Diets Excluding Imputed Data **eFigure 25.** The Forest Plot for Changes in HbA1c, Grouped by Control Diets Excluding Imputed Data **eFigure 26.** The Forest Plot for Changes in HbA1c, Grouped by Energy Restriction Excluding Imputed Data **eFigure 27.** The Forest Plot for Changes in HbA1c, Grouped by Physical Activity Excluding Imputed Data **eFigure 28.** The Forest Plot for Changes in HbA1c, Grouped by Medication Use Excluding Imputed Data **eFigure 29.** The Forest Plot for Changes in HbA1c, Grouped by Analysis Method Excluding Imputed Data **eFigure 30.** Treatment Effect of HbA1c Across Baseline HbA1c **eFigure 31.** Funnel Plot of HbA1c **eFigure 32.** The Forest Plot for Changes in SBP, Grouped by Vegetarian Diets **eFigure 33.** The Forest Plot for Changes in SBP, Grouped by Control Diets **eFigure 34.** The Forest Plot for Changes in SBP, Grouped by Energy Restriction **eFigure 35.** The Forest Plot for Changes in SBP, Grouped by Physical Activity **eFigure 36.** The Forest Plot for Changes in SBP, Grouped by Medication Use **eFigure 37.** The Forest Plot for Changes in SBP, Grouped by Analysis Method **eFigure 38.** The Forest Plot for Changes in SBP, Grouped by Disease Status Excluding Imputed Data **eFigure 39.** The Forest Plot for Changes in SBP, Grouped by Vegetarian Diets Excluding Imputed Data **eFigure 40.** The Forest Plot for Changes in SBP, Grouped by Control Diets Excluding Imputed Data **eFigure 41.** The Forest Plot for Changes in SBP, Grouped by Energy Restriction Excluding Imputed Data **eFigure 42.** The Forest Plot for Changes in SBP, Grouped by Physical Activity Excluding Imputed Data **eFigure 43.** The Forest Plot for Changes in SBP, Grouped by Medication Use Excluding Imputed Data **eFigure 44.** The Forest Plot for Changes in SBP, Grouped by Analysis Method Excluding Imputed Data **eFigure 45.** Treatment Effect of SBP Across Baseline SBP **eFigure 46.** Funnel Plot of SBP **eFigure 47.** The Forest Plot for Changes in Body Weight, Grouped by Disease Status **eFigure 48.** The Forest Plot for Changes in Body Weight, Grouped by Energy Restriction **eFigure 49.** The Forest Plot for Changes in Body Weight, Grouped by Control Diets **eFigure 50.** The Forest Plot for Changes in Body Weight, Grouped by Disease Status Excluding Imputed Data **eFigure 51.** The Forest Plot for Changes in Weight, Grouped by Energy Restriction Excluding Imputed Data **eFigure 52.** The Forest Plot for Changes in Weight, Grouped by Control Diets Excluding Imputed Data **eFigure 53.** Treatment Effect of Body Weight Across Baseline Body Weight **eFigure 54.** Funnel Plot of Body Weight **eFigure 55.** The Forest Plot for Changes in Energy Intake in Studies Without Energy Restriction, Grouped by Control Diets **eFigure 56.** The Forest Plot for Changes in Energy Intake in Studies Without Energy Restriction, Grouped by Control Diets Excluding Imputed Data **eFigure 57.** Funnel Plot of Energy Intake in Studies Without Energy Restriction **eFigure 58.** Risk of Bias Assessment of Included Randomized Controlled Trials

Supplement 2. **eAppendix.** Data Set (Separate File)

Supplement 3.
Data Sharing Statement
