# Supplementary material for: Vegetarian Dietary Patterns and Cardiometabolic Risk in People With or at High Risk of Cardiovascular Disease: A Systematic Review and Meta-analysis
Source: JAMA Netw Open. 2023 Jul 25;6(7):e2325658. doi: 10.1001/jamanetworkopen.2023.25658 (PMC10369207; doi:10.1001/jamanetworkopen.2023.25658)
Supplement: Supplement 1. — eMethods. eTable 1. Description of PICOS Criteria eTable 2. Complete Search Strategies for Different Databases eTable 3. Inclusion and Exclusion Criteria of Included Studies eTable 4. Study Characteristics of Ongoing Clinical Trials That Meet Our Eligibility Criteria eTable 5. Participant Characteristics and Description of Included Studies eTable 6. Dietary Data of Included Studies eTable 7. Detailed Risk of Bias Assessment of Included Studies eTable 8. GRADE Assessment in People With CVD or People at High Risk of CVD eTable 9. GRADE Assessment in People at High Risk of CVD eTable 10. GRADE Assessment in People With T2DM eTable 11. GRADE Assessment in People With CVD eTable 12. Studies Excluded at Stage 2 With Reasons eTable 13. Reported Conflicts of Interest (COI) and Funding Sources of Included Studies eTable 14. Primary and Secondary Outcomes of Included Studies eFigure 1. The Forest Plot for Changes in LDL-C, Grouped by Disease Status Excluding Ornish’s Study eFigure 2. The Forest Plot for Changes in LDL-C, Grouped by Vegetarian Diets eFigure 3. The Forest Plot for Changes in LDL-C, Grouped by Control Diets eFigure 4. The Forest Plot for Changes in LDL-C, Grouped by Energy Restriction eFigure 5. The Forest Plot for Changes in LDL-C, Grouped by Physical Activity eFigure 6. The Forest Plot for Changes in LDL-C, Grouped by Medication Use eFigure 7. The Forest Plot for Changes in LDL-C, Grouped by Analysis Method eFigure 8. The Forest Plot for Changes in LDL-C, Grouped by Disease Status Excluding Imputed Data eFigure 9. The Forest Plot for Changes in LDL-C, Grouped by Vegetarian Diets Excluding Imputed Data eFigure 10. The Forest Plot for Changes in LDL-C, Grouped by Control Diets Excluding Imputed Data eFigure 11. The Forest Plot for Changes in LDL-C, Grouped by Energy Restriction Excluding Imputed Data eFigure 12. The Forest Plot for Changes in LDL-C, Grouped by Physical Activity Excluding Imputed Data eFigure 13. The Forest Plot for Changes in LDL-C, Grouped by M [file jamanetwopen-e2325658-s001.pdf]

Supplemental Online Content

Wang T, Kroeger CM, Cassidy S, et al. Vegetarian dietary patterns and cardiometabolic risk in people with or at high risk of cardiovascular disease: a systematic review and meta-analysis. *JAMA Netw Open.*
2023;6(7):e2325658. doi:10.1001/jamanetworkopen.2023.25658

eMethods.

eTable 1. Description of PICOS Criteria

eTable 2. Complete Search Strategies for Different Databases

eTable 3. Inclusion and Exclusion Criteria of Included Studies

eTable 4. Study Characteristics of Ongoing Clinical Trials That Meet Our Eligibility Criteria

eTable 5. Participant Characteristics and Description of Included Studies

eTable 6. Dietary Data of Included Studies

eTable 7. Detailed Risk of Bias Assessment of Included Studies

eTable 8. GRADE Assessment in People With CVD or People at High Risk of CVD

eTable 9. GRADE Assessment in People at High Risk of CVD

eTable 10. GRADE Assessment in People With T2DM

eTable 11. GRADE Assessment in People With CVD

eTable 12. Studies Excluded at Stage 2 With Reasons

eTable 13. Reported Conflicts of Interest (COI) and Funding Sources of Included Studies

eTable 14. Primary and Secondary Outcomes of Included Studies

eFigure 1. The Forest Plot for Changes in LDL-C, Grouped by Disease Status Excluding Ornish's Study

eFigure 2. The Forest Plot for Changes in LDL-C, Grouped by Vegetarian Diets

eFigure 3. The Forest Plot for Changes in LDL-C, Grouped by Control Diets

eFigure 4. The Forest Plot for Changes in LDL-C, Grouped by Energy Restriction

eFigure 5. The Forest Plot for Changes in LDL-C, Grouped by Physical Activity

eFigure 6. The Forest Plot for Changes in LDL-C, Grouped by Medication Use

eFigure 7. The Forest Plot for Changes in LDL-C, Grouped by Analysis Method

© 2023 Wang T et al. *JAMA Network Open.*

eFigure 8. The Forest Plot for Changes in LDL-C, Grouped by Disease Status Excluding Imputed Data

eFigure 9. The Forest Plot for Changes in LDL-C, Grouped by Vegetarian Diets Excluding Imputed Data

eFigure 10. The Forest Plot for Changes in LDL-C, Grouped by Control Diets Excluding Imputed Data

eFigure 11. The Forest Plot for Changes in LDL-C, Grouped by Energy Restriction Excluding Imputed Data

eFigure 12. The Forest Plot for Changes in LDL-C, Grouped by Physical Activity Excluding Imputed Data

eFigure 13. The Forest Plot for Changes in LDL-C, Grouped by Medication Use Excluding Imputed Data

eFigure 14. The Forest Plot for Changes in LDL-C, Grouped by Analysis Method Excluding Imputed Data

eFigure 15. Treatment Effect of LDL-C Across Baseline LDL-C

eFigure 16. Funnel Plot of LDL-C

eFigure 17. The Forest Plot for Changes in HbA1c, Grouped by Vegetarian Diets

eFigure 18. The Forest Plot for Changes in HbA1c, Grouped by Control Diets

eFigure 19. The Forest Plot for Changes in HbA1c, Grouped by Energy Restriction

eFigure 20. The Forest Plot for Changes in HbA1c, Grouped by Physical Activity

eFigure 21. The Forest Plot for Changes in HbA1c, Grouped by Medication Use

eFigure 22. The Forest Plot for Changes in HbA1c, Grouped by Analysis Method

eFigure 23. The Forest Plot for Changes in HbA1c, Grouped by Disease Status Excluding Imputed Data

eFigure 24. The Forest Plot for Changes in HbA1c, Grouped by Vegetarian Diets Excluding Imputed Data

eFigure 25. The Forest Plot for Changes in HbA1c, Grouped by Control Diets Excluding Imputed Data

eFigure 26. The Forest Plot for Changes in HbA1c, Grouped by Energy Restriction Excluding Imputed Data

eFigure 27. The Forest Plot for Changes in HbA1c, Grouped by Physical Activity Excluding Imputed Data

eFigure 28. The Forest Plot for Changes in HbA1c, Grouped by Medication Use Excluding Imputed Data

© 2023 Wang T et al. *JAMA Network Open.*

eFigure 29. The Forest Plot for Changes in HbA1c, Grouped by Analysis Method Excluding Imputed Data

eFigure 30. Treatment Effect of HbA1c Across Baseline HbA1c

eFigure 31. Funnel Plot of HbA1c

eFigure 32. The Forest Plot for Changes in SBP, Grouped by Vegetarian Diets

eFigure 33. The Forest Plot for Changes in SBP, Grouped by Control Diets

eFigure 34. The Forest Plot for Changes in SBP, Grouped by Energy Restriction

eFigure 35. The Forest Plot for Changes in SBP, Grouped by Physical Activity

eFigure 36. The Forest Plot for Changes in SBP, Grouped by Medication Use

eFigure 37. The Forest Plot for Changes in SBP, Grouped by Analysis Method

eFigure 38. The Forest Plot for Changes in SBP, Grouped by Disease Status Excluding Imputed Data

eFigure 39. The Forest Plot for Changes in SBP, Grouped by Vegetarian Diets Excluding Imputed Data

eFigure 40. The Forest Plot for Changes in SBP, Grouped by Control Diets Excluding Imputed Data

eFigure 41. The Forest Plot for Changes in SBP, Grouped by Energy Restriction Excluding Imputed Data

eFigure 42. The Forest Plot for Changes in SBP, Grouped by Physical Activity Excluding Imputed Data

eFigure 43. The Forest Plot for Changes in SBP, Grouped by Medication Use Excluding Imputed Data

eFigure 44. The Forest Plot for Changes in SBP, Grouped by Analysis Method Excluding Imputed Data

eFigure 45. Treatment Effect of SBP Across Baseline SBP

eFigure 46. Funnel Plot of SBP

eFigure 47. The Forest Plot for Changes in Body Weight, Grouped by Disease Status

eFigure 48. The Forest Plot for Changes in Body Weight, Grouped by Energy Restriction

eFigure 49. The Forest Plot for Changes in Body Weight, Grouped by Control Diets

eFigure 50. The Forest Plot for Changes in Body Weight, Grouped by Disease Status Excluding Imputed Data

eFigure 51. The Forest Plot for Changes in Weight, Grouped by Energy Restriction Excluding Imputed Data

© 2023 Wang T et al. *JAMA Network Open.*

eFigure 52. The Forest Plot for Changes in Weight, Grouped by Control Diets Excluding Imputed Data

eFigure 53. Treatment Effect of Body Weight Across Baseline Body Weight

eFigure 54. Funnel Plot of Body Weight

eFigure 55. The Forest Plot for Changes in Energy Intake in Studies Without Energy Restriction, Grouped by Control Diets

eFigure 56. The Forest Plot for Changes in Energy Intake in Studies Without Energy Restriction, Grouped by Control Diets Excluding Imputed Data

eFigure 57. Funnel Plot of Energy Intake in Studies Without Energy Restriction

eFigure 58. Risk of Bias Assessment of Included Randomized Controlled Trials

This supplemental material has been provided by the authors to give readers additional information about their work.

Methods

Search strategy and selection criteria

This systematic review and meta-analysis has been registered with PROSPERO (CRD 42021218348). We followed the PRISMA guidelines[1] and the AMSTAR-2 checklist[2] for this study.

The PICOS criteria is described in eTable 1, and studies needed to be published in English or Chinese to be included. We searched EMBASE, MEDLINE, CINAHL and CENTRAL (Cochrane Central Register of Controlled Trials), from inception until 31st July 2021 (eTable 2 shows full search strategies). Hand searches of reference lists of reviews, protocols, abstracts, and grey literature (e.g., websites mentioning relevant studies) were performed to supplement searches. The authors of the ongoing trials and abstracts were contacted at least three times to retrieve preliminary findings and full manuscripts.

Retrieved articles were uploaded to the Covidence review platform, and duplicates were removed. The screening process was standardized, and training was provided to all reviewers by the principal investigator (TW). All titles, abstracts, full-texts, and data extraction were independently screened or extracted by the principal investigator (TW) and one other investigator (SC, CK, SM, RR). Discrepancies were resolved by a third investigator.

We followed the Cochrane Handbook[3] and extracted these data: study identifiers, participants' characteristics, inclusion and exclusion criteria, dietary intervention, exercise and medication instructions, primary and secondary outcomes, funding sources and conflict of interest statement, and pre-, post-intervention values and changes in weight, body mass index (BMI), SBP, LDL-C, HbA1c, dietary intake and medication use. If this information could not be found from the published sources, the corresponding author was contacted with at least three attempts, with a waiting period of one month to extract data and clarify information as needed.

Data analysis

The primary outcomes are the mean differences between groups in changes (pre vs post intervention) in LDL-C, HbA1c and SBP. Following the recommendations of the Cochrane Handbook [3], we selected a maximum of three primary outcomes; these biomarkers are primary pharmacological targets for the prevention and treatment of CVD[4,5]. The secondary outcomes are changes in body weight, and energy intake.

To examine the effect of interventions on primary outcomes, we obtained the mean and standard deviation (SD) of changes (post minus pre) in intervention and comparison groups. For studies reporting the median (interquartile range), we converted results into the mean (SD) following the method proposed by Wan *et al.*[6] when values could not be obtained from authors. The Cochrane guidelines[3] were used for SDs of change imputation using data available from similar studies (e.g. studies focused on the same population, i.e. people with CVD or T2DM or at high risk of CVD) when they could not be obtained from authors. The Cochrane guideline[3] were also followed for data extraction from cross-over design studies and only data from the first period was extracted if the carry-over effect was considered significant.

The units of LDL-C, HbA1c, and energy were standardized to mg/dL[7] (from mmol/L to mg/dL, multiplied by 38.67), % (requested from authors), and kcal (from kJ to kcal, divided by 4.18), respectively. We considered results to be clinically significant if LDL-C, HbA1c, and SBP changed by 10mg/dL, 0·5%, and 5mmHg, respectively[8]. The "meta" package of R (version 1.4.1717)[9] was used to perform meta-analysis and meta-regression. The R script and dataset are available in the appendix. A random-effects model was employed, implemented using the 'metacont' function for mean differences. We estimated the overall pooled effect size based on inverse-variance weighting using a restricted maximum likelihood estimator (REML) for the among-study heterogeneity [10]. Confidence intervals (CIs) are at the 95% level and estimated based on a standard-normal distribution (i.e., default method in the meta package). The total heterogeneity was quantified as τ^2 (i.e., variance among effect sizes not attributable to sampling). The statistical significance for heterogeneity was assessed by a modified Q test, which used the Farebrother method to obtain the distribution of Q values, as recommended for mean differences by Kulinskaya *et al.*[11]. Studies were grouped according to patients' conditions (CVD, T2DM or people with ≥2 CVD risk factors) and type of vegetarian diets in the meta-analysis. Exploratory sub-group analyses assessed the effect of: a) different control diets; b) energy restriction; c) physical activity; d) intervention with and without medication changes; e) analyses performed following intention-to-treat versus completers approaches. We performed additional exploratory meta-analysis to examine the impact of interventions on weight with and without energy restriction. In studies that did not prescribe an energy-restricted vegetarian diet, we evaluated their effects on energy intake compared to different control diets. We carried out mixed-effects meta-regression ('metareg' function) with bubble plots to explore if baseline LDL-C, HbA1c, SBP, and body weight could explain heterogeneity between studies.

Publication bias was evaluated by visual inspection of the asymmetry of funnel plots. Results were considered statistically significant if the resulting p-values were <0·05. We used the Cochrane Risk of Bias Assessment Tool 2.0[12] and

the GRADE[13] (Grading of Recommendations, Assessment, Development, and Evaluation) tool to assess the risk of bias and evaluate the overall quality of evidence for primary outcomes and body weight, respectively. Two independent reviewers appraised each study (TW and either SC, CK, SM, RR or AM), and a third investigator was involved in resolving discrepancies. We constructed summary tables using GRADEpro[14] and assessed these domains following the GRADE guidelines[13,15]: risk of bias, inconsistency between studies, indirectness and imprecision of the outcomes, and potential publication bias. We built four tables for the GRADE assessment: overall quality, people with high risk of CVDs, people with T2DM, and people with CVDs. We did not perform the GRADE assessment if there were less than three studies for that outcome.

Ethical review of study and informed consent of study participants

Given the nature of secondary data extraction and analysis, ethical review and informed consent of participants are not required in this study.

© 2023 Wang T et al. *JAMA Network Open.*

eTable 1. Description of PICOS criteria

PICOS criteria	Description
**P**opulation	Adults living in the community setting aged ≥18 years with: a). CVDs; or b). T2DM; or c) at least two risk factors* of CVDs<br>Risk factors are defined as [16-19]:<br>• Overweight: BMI ⩾ 25<br>• High blood pressure (systolic ⩾130mmHg; diastolic ⩾ 80mmHg) [20]<br>• Blood lipid [21]: TC ⩾ 200mg/dL; or LDL ⩾ 100mg/dL; or TG ⩾ 150mg/dL<br>• HbA1c ⩾ 5·7% [22]<br>• Metabolic syndrome (if ⩾ 3 met) [23]:<br>◦ Waist circumference: > 40 inches (men) or > 35 inches (women)<br>◦ Blood pressure over 130/85 mmHg<br>◦ TG ⩾ 150 mg/dL<br>◦ Fasting HDL < 40 mg/dL (men) or 50 mg/dL (women)<br>◦ Fasting blood sugar ⩾ 100 mg/dL
**I**ntervention	The vegetarian diet, in which animal products are not allowed except for fish and seafood (the pesco-vegetarian diet), eggs (the ovo-vegetarian diet or the lacto-ovo vegetarian diet), and dairy products (the lacto-vegetarian diet or the lacto-ovo vegetarian diet)
**C**omparator	The non-vegetarian diet
**O**utcome	Changes in SBP, LDL or HbA1c and dietary intake, from pre- to post-intervention
**S**tudy design	Randomised controlled trials ≥ 8 weeks, given its power to assess the effect of a vegetarian diet

BMI, body mass index; CVD, cardiovascular disease; HbA1c, hemoglobin A1c; HDL, high-density lipoprotein cholesterol; LDL, low-density lipoprotein cholesterol; SBP, systolic blood pressure; T2DM, type 2 diabetes mellitus; TC, total cholesterol; TG, triglyceride

© 2023 Wang T et al. *JAMA Network Open.*

eTable 2. Complete search strategies for different databases

Databases	MEDLINE	EMBASE	CINAHL	Cochrane Central Register of Controlled Trials (CENTRAL)
**#**	**Searches (Results)**	**Searches (Results)**	**Searches (Results)**	**Searches (Results)**
1	coronary disease/ or coronary artery disease/ (191771)	exp vegetarian diet/ (4472)	(MH "CoronaryDisease+") OR (MH"CardiovascularDiseases+") OR (MH"Vascular Diseases+")OR (MH "MyocardialInfarction+") (629531)	exp Coronary Disease/ (13685)
2	coronary heart disease*.mp. (51418)	plant based diet*.mp. (1124)	(MH "Heart Diseases+") (295938)	exp Coronary Artery Disease/ (6496)
3	cardiovascular diseases/ or cardiovascular abnormalities/ or heart diseases/ or vascular diseases/ (260016)	exp vegan diet/ or exp vegetarian/ or exp vegan/ (3251)	"coronary heart disease*" (33834)	coronary heart disease*.mp. (7755)
4	heart disease*.mp. (244319)	vegan*.mp. (2091)	(MH "CardiovascularAbnormalities+") (34518)	exp cardiovascular diseases/ or exp cardiovascular abnormalities/ or exp heart diseases/ or exp vascular diseases/ (107566)
5	exp Stroke/ (138850)	vegetarian*.mp. (7788)	(MH "VascularDiseases+") (427307)	heart disease*.mp. (23472)
6	cerebrovascular accident.mp. (4668)	semi-vegetarian*.mp. (106)	(MH "Stroke+") (74183)	exp Stroke/ (9836)
7	exp Myocardial Infarction/ (177456)	pesco-vegetarian*.mp. (58)	"cerebrovascularaccident" (61456)	cerebrovascular accident.mp. (14685)
8	heart attack*.mp. (5714)	lacto-vegetarian*.mp. (167)	"heart disease*" (93957)	exp Myocardial Infarction/ (10847)
9	exp Death, Sudden, Cardiac/ (15787)	vegan diet*.mp. (984)	"heart attack*" (46219)	heart attack*.mp. (1233)
10	exp Triglycerides/ (77510)	vegetarian diet*.mp. (4791)	(MH "Death, Sudden,Cardiac") (6885)	exp Death, Sudden, Cardiac/ (635)
11	exp Cholesterol/ (161388)	ovo-vegetarian*.mp. (316)	(MH "Triglycerides") (15038)	exp Triglycerides/ (6344)
12	exp Blood Pressure/ (292805)	lacto-ovo vegetarian*.mp. (308)	(MH "Cholesterol+") OR(MH "Lipoproteins, LDL Cholesterol") OR (MH"Lipoproteins, HDLCholesterol") (29137)	exp Cholesterol, LDL/ or exp Cholesterol, VLDL/ or exp Cholesterol/ or exp Cholesterol, HDL/ (10207)
13	exp Blood Glucose/ (166220)	PBD.tw. (2656)	(MH "Blood Pressure+") (53929)	exp Blood Pressure/ (27585)
14	fasting glucose.mp. (17602)	1 or 2 or 3 or 4 or 5 or 6 or 7 or 8 or 9 or 10 or 11 or 12 or 13 (11879)	(MH "Blood Glucose") (39949)	exp Blood Glucose/ or fasting glucose.mp. (19820)
15	exp Glycated Hemoglobin A/ (35762)	coronary disease.mp. or exp coronary artery disease/ (347474)		HbA1c.mp. (18010)
16	HbA1c.mp. (35606)	coronary heart disease*.mp. (73390)		exp Hyperlipidemias/ (6430)

© 2023 Wang T et al. *JAMA Network Open.*

17	stroke.mp. (303219)	exp cardiovascular disease/ (4365472)	“fasting glucose” OR (MH”Blood Glucose”) (43025)	hyperlipid?emia.mp. (5089)
18	CVD.tw. (37308)	cardiovascular disease*.mp. (416927)	(MH “Hemoglobin A,Glycosylated”) OR”HbA1c” (24897)	high blood pressure.mp. or exp Hypertension/ (19890)
19	heart failure*.mp. (213594)	exp heart disease/ (1963261)	(MH “Hyperlipidemia+”) (21760)	exp non insulin dependent diabetes mellitus/ (17877)
20	myocardial infarction.mp. (249631)	heart disease*.mp. (429147)	(MH “Hypertension+”) (85038)	T2DM.tw. (6609)
21	(cardiovascular disease* or cardiovascular abnormalit* or heart disease* or vascular disease*).mp. (566674)	exp vascular disease/ (2637814)	(MH “Diabetes Mellitus,Type 2”) (65156)	NIDDM.tw. (1085)
22	Atrial Fibrillation/ (57164)	vascular disease*.mp. (126351)	“T2DM” (6845)	Non insulin dependent diabetes mellitus.tw. (1421)
23	atrial fibrillation*.mp. (85972)	exp cerebrovascular accident/ (222808)	“NIDDM” (51369)	(Type 2 diabet$ or Type ii diabet$).tw. (37838)
24	Diabetes Mellitus, Type 2/ (136259)	cerebrovascular accident*.mp. (221804)		exp Diabetes Mellitus, Type 2/ (17877)
25	type 2 diabete*.mp. (132954)	exp heart infarction/ (398603)	“non insulin dependent diabetes mellitus” (51319)	stroke.mp. (58522)
26	Cholesterol, HDL/ (28623)	heart attack*.mp. (8473)	“(type 2 or type II) diabet*” (0)	CVD.tw. (5744)
27	Cholesterol TC.mp. (9438)	exp sudden cardiac death/ (16758)	"CVD" (56108)	heart failure*.mp. (31334)
28	cholesterol TG.mp. (465)	exp triacylglycerol/ (208112)	(MH "Heart Failure+") OR "heart failure*" (70016)	myocardial infarction.mp. (31898)
29	Cholesterol, LDL/ (27953)	exp cholesterol/ (328489)	(MH "Atrial Fibrillation")OR "Atrial Fibrillation" (37543)<br>"high blood pressure" (122803)<br>(MH "Obesity+") OR"overweight" (117298)	(cardiovascular disease* or cardiovascular abnormalit* or heart disease* or vascular disease*).mp. (56473)
30	cholesterol*.mp. (294142)	exp blood pressure/ (607283)	S1 OR S2 OR S3 OR S4OR S5 OR S6 OR S7 OR S8 OR S9 OR S10 ORS11 OR S12 OR S13 ORS14 OR S15 OR S16 ORS17 OR S18 OR S19 ORS20 OR S21 OR S22 ORS23 OR S24 OR S25 ORS26 OR S27 OR S28 (168)	Atrial Fibrillation/ (4721)
31	Hypertension/ (237017)	exp glucose blood level/ (268775)		atrial fibrillation*.mp. (13745)
32	high blood pressure*.mp. (15590)	fasting glucose.mp. (30688)		Cholesterol TC.mp. (2055)
33	hypertension*.mp. (499685)	exp hemoglobin A1c/ (111523)		cholesterol TG.mp. (136)
34	exp Overweight/ or exp Obesity/ (227431)	exp hyperlipidemia/ (164725)		cholesterol*.mp. (38668)
35	overweight*.mp. (77136)	hyperlipid?emia.mp. (88786)	"PBD" (214)<br>"veganism" (94)	high blood pressure*.mp. (2418)
36	obesity*.mp. (335888)	high blood pressure.mp. or exp hypertension/ (778660)	"lacto-ovo vegetarian*" (3503)<br>"ovo-vegetarian*" (97)	hypertension*.mp. (64404)
37	1 or 2 or 3 or 4 or 5 or 6 or 7 or 8 or 9 or 10 or 11 or 12 or 13 or 14 or 15 or 16 or 17 or 18 or 19 or 20 or 21 or 22 or 23 or 24 or 25 or 26 or 27 or	exp non insulin dependent diabetes mellitus/ (265260)	"vegetarian diet*" (3826)<br>"vegan diet*" (413)	exp Overweight/ (15844)

© 2023 Wang T et al. *JAMA Network Open.*

	28 or 29 or 30 or 31 or 32 or 33 or 34 or 35 or 36 (2560350)		"lacto-vegetarian*" (3487)	
38	exp Vegans/ (92)	T2DM.tw. (39431)	"pesco-vegetarian*" (30)	exp Obesity/ (14018)
39	exp Vegetarians/ (241)	NIDDM.tw. (8060)		overweight*.mp. (17852)
40	exp Diet, Vegetarian/ (3360)	Non insulin dependent diabetes mellitus.tw. (7808)		obesity*.mp. (39590)
41	exp Diet, Vegan/ (190)	(Type 2 diabet$ or Type ii diabet$).tw. (226030)	"semi-vegetarian*" (50)<br>"vegetarian*" (8282)<br>"vegan*" (2060)<br>"plant-based diet*" (995)	1 or 2 or 3 or 4 or 5 or 6 or 7 or 8 or 9 or 10 or 11 or 12 or 13 or 14 or 15 or 16 or 17 or 18 or 19 or 20 or 21 or 22 or 23 or 24 or 25 or 26 or 27 or 28 or 29 or 30 or 31 or 32 or 33 or 34 or 35 or 36 or 37 or 38 or 39 or 40 (344595)
42	semi-vegetarian*.mp. (72)	stroke.mp. (464494)		exp diet, vegetarian/ (198)
43	pesco-vegetarian*.mp. (38)	CVD.tw. (59012)		plant based diet*.mp. (136)
44	lacto-vegetarian*.mp. (122)	sudden cardiac*.mp. (36202)		plant-based diet*.mp. (136)
			"plant based diet*" (995)	
45	veganism.tw. (75)	heart failure*.mp. (421492)	(MH "Plant-Based Diet")OR (MH "Vegetarianism") (7533)	vegan*.mp. (237)
46	vegetarianism.tw. (430)	myocardial infarction.mp. (302811)		vegetarian*.mp. (632)
47	plant based diet*.mp. (923)	(cardiovascular disease* or cardiovascular abnormalit* or heart disease* or vascular disease*).mp. (897555)	S30 OR S31 OR S32 ORS33 OR S34 OR S35 ORS36 OR S37 OR S38 ORS39 OR S40 OR S41 ORS42 OR S43 (8908)	semi-vegetarian*.mp. (10)
48	vegan*.mp. (1371)	Atrial Fibrillation/ (65837)	S29 AND S44 (1062)	pesco-vegetarian*.mp. (10)
49	vegetarian*.mp. (5362)	atrial fibrillation*.mp. (151907)		lacto-vegetarian*.mp. (20)
50	vegan diet*.mp. (538)	Diabetes Mellitus, Type 2/ (63061)		vegan diet*.mp. (138)
51	vegetarian diet*.mp. (1661)	type 2 diabete*.mp. (205635)		vegetarian diet*.mp. (314)
52	PBD.tw. (1846)	Cholesterol, HDL/ (94811)		lacto-ovo vegetarian*.mp. (46)
53	ovo-vegetarian*.mp. (235)	Cholesterol TC.mp. (14442)		ovo-vegetarian*.mp. (46)
54	lacto-ovo vegetarian*.mp. (232)	cholesterol TG.mp. (753)		veganism.tw. (1)
55	38 or 39 or 40 or 41 or 42 or 43 or 44 or 45 or 46 or 47 or 48 or 49 or 50 or 51 or 52 or 53 or 54 (8201)	Cholesterol, LDL/ (92627)		vegetarianism.tw. (24)
56	37 and 55 (2096)	cholesterol*.mp. (455321)		PBD.tw. (138)
57		hypertension*.mp. (938542)		42 or 43 or 44 or 45 or 46 or 47 or 48 or 49 or 50 or 51 or 52 or 53 or 54 or 55 or 56 (978)
58		exp obesity/ (542882)		41 and 57 (488)
59		overweight*.mp. (111538)		
60		obesity*.mp. (570942)		

© 2023 Wang T et al. *JAMA Network Open.*

61	15 or 16 or 17 or 18 or 19 or 20 or 21 or 22 or 23 or 24 or 25 or 26 or 27 or 28 or 29 or 30 or 31 or 32 or 33 or 34 or 35 or 36 or 37 or 38 or 39 or 40 or 41 or 42 or 43 or 44 or 45 or 46 or 47 or 48 or 49 or 50 or 51 or 52 or 53 or 54 or 55 or 56 or 57 or 58 or 59 or 60 (5750388)
62	14 and 61 (4119)

© 2023 Wang T et al. *JAMA Network Open.*

eTable 3. Inclusion and exclusion criteria of included studies

Author, year	Inclusion criteria	Exclusion criteria
Aldana *et al.*, 2006 [24]	Participants were included if they had ≥ 2 of the following criteria: chest pain lasting ≥ 30 mins continuously; electrocardiogram changes typical of an evolving MI (ST segment elevation or depression, evolving Q waves, or symmetric inversion of T waves); elevated levels of cardiac enzymes.<br><br>Patients were also accepted if they demonstrated any of the following: MI within the past 12 months; coronary artery bypass graft surgery; stent placement; rotoblator within the prior 12 months; stable angina; or pacemakers associated with ≥ 1 of the above diagnoses.	Exclusion criteria included history of substance abuse disorder without documentation of at least 1 year of abstinence; history of psychiatric disorder without documentation of at least 1 year of stability; impaired cognitive function such as dementia or delirium; participation in other lipid-lowering or lifestyle modification trials; and others which were previously documented.
Barnard *et al.*, 2006 [25-28]	Individuals with T2DM, defined by a fasting plasma glucose concentration > 6·9 mmol/l on two occasions or a prior diagnosis of T2DM with the use of hypoglycemic medications for ≥ 6 months.	HbA1c < 6·5 or > 10·5%; use of insulin for > 5 years; current smoking, alcohol or drug abuse; pregnancy; unstable medical status; current use of a LF vegetarian diet.
Barnard *et al.*, 2018 [29]	(1) A diagnosis of T2DM, defined by a fasting plasma glucose concentration ≥ 126 mg/dL on two occasions or a prior physician's diagnosis of T2DM with the use of hypoglycemic medications for ≥ 6 months; (2) HbA1c: 6·5-10·5%; (3) ≥ 18 years; (4) ability and willingness to be assigned to either a LF, vegan, or portion-controlled eating plan and participate in all components of the study; and (5) diabetes medications unchanged for 1 month before volunteering for the study.	(1) BMI > 45 kg/m^2; (2) alcohol consumption of > 2 drinks/d or the equivalent, episodic increased drinking (e.g., > 2 drinks/d on weekends), or a history of alcohol abuse or dependency followed by any current use; (3) use of recreational drugs in the past 6 months; (4) pregnancy; (5) signs or symptoms of acute uncontrolled diabetes, including but not limited to polyuria, polydipsia, blurred vision, or uncontrolled weight loss; (6) unstable medical status; (7) already following a LF, vegetarian eating pattern; and (8) lack of English fluency.
Barnard *et al.*, 2021 [30]	Adults with a BMI between 28 and 40 kg/m^2.	Type 1 diabetes; smoking, alcohol or drug abuse; pregnancy or lactation, and current use of a vegan or Mediterranean diet.
Bunner *et al.*, 2015 [31]	Age 18–65 years, diagnosis of T2DM, and diagnosis or symptoms of painful diabetic neuropathy for ≥ 6 months.	Vitamin B12 deficiency, alcohol consumption of > 2 drinks/d, use of recreational drugs in the past 6 months, pregnancy, unstable medical or psychiatric illness, current adherence to a vegan diet and inability or unwillingness to participate in all components of the study.
Burke *et al.*, 2006 [32-34]	Individuals were eligible if they were 18-55 years of age, had a BMI 27-43 kg/m^2, were willing to be randomized to one of the two treatment preference groups and one of the two dietary groups, and successfully completed a 5-day food diary.	Individuals who had a serious illness or unstable condition for which physician supervision of diet and exercise prescription was needed; had a cardiovascular or orthopedic condition that would require physician clearance before participation; had limitations precluding ability to exercise; were pregnant or intended to become pregnant in the next 18 months; were being treated for a psychological disorder; reported alcohol intake of ≥ 4 drinks/d; were participating or had recently participated in a weight loss treatment program or used weight loss medication; reported no regular intake of meat, fish, and fowl; had a serious binge eating problem; and did not at least moderately prefer one of the dietary treatments (SBT or SBT+LOV) over the other.

© 2023 Wang T et al. *JAMA Network Open.*

Author, year	Inclusion criteria	Exclusion criteria
Garousi *et al.*, 2021 [35]	Have been diagnosed with NAFLD, to be motivated to participate in the study, aged 20-55 years, overweight (BMI > 25 kg/m^2), and non-consumers of alcohol (< 40 g/week). NAFLD was diagnosed by an ultrasonography scan and associated with a persistently elevated ALT concentration > 19 U/L for women and > 30 U/L for men.	Participants were excluded if they were following a special diet, pregnant, breastfeeding, or suffering from viral hepatitis, diabetes mellitus, untreated hypothyroidism or other causes of chronic liver disease.
Kahleova *et al.*, 2010 [36]	T2DM, age 30–70 years, HbA1c 6-11% (42–97 mmol ⁄ mol), BMI 25-53 kg ⁄m^2, and willingness to change dietary habits and follow a prescribed exercise programme.	HbA1c < 6% (< 42 mmol ⁄ mol) or > 11% (> 97 mmol ⁄mol), use of insulin, abuse of alcohol or drugs, pregnancy, lactation, or current use of a vegetarian diet.
Kahleova *et al.*, 2020 [37]	Adults aged 25-75 years with a BMI of 28-40 kg/m^2.	Diabetes, smoking, alcohol or drug use, pregnancy or lactation, and current use of a vegan diet. The additional exclusion criteria for the subset of participants undergoing the proton magnetic resonance spectroscopy were the presence of anymetal implant, claustrophobia, BMI > 38 kg/m^2, and waist circumference > 102 cm.
Lee *et al.*, 2016 [38]	Age 30–70 years; use of hypoglycemic medications for ≥ 6 months; and HbA1c level of 6·0–11·0%.	Increased dose of hypoglycemic medication or the addition of a new drug in the regimen during the last 2 months; current vegetarian status; pregnancy; or severe complications such as chronic renal failure.
Liao *et al.*, 2007 [39]	20–60 y of age, have a BMI > 26 kg/m^2, and have no history of chronic disease, including CVD, kidney disease, and diabetes mellitus.	Female subjects could not be pregnant or breastfeeding.
Mahon *et al.*, 2007 [40]	NR. Assume postmenopausal women who do not meet exclusion criteria.	Male, age < 50 y or > 80 y, < 2 y postmenopausal, BMI < 25 and > 34 kg/m^2 at the time of screening, a smoker and clinically abnormal kidney, liver, or heart function. Subjects could not be individuals with diabetes or unstable thyroid disease, in abnormal protein or hematological status, nor receiving insulin replacement therapy or anti-inflammatory steroid medications.
Mishra *et al.*, 2013 [41,42]	>18 years of age with a BMI ≥ 25 kg/m^2 and/or a previous diagnosis of T2DM.	Current alcohol or drug abuse, pregnancy, history of severe mental illness, unstable medical status, current adherence to a LF, vegetarian diet, participation in the previous GEICO two-site study and inability to attend weekly meetings
Nicholson *et al.*, 1999 [43]	T2DM, age > 25 years, willingness to attend all components of the study, and residence within commuting distance of Georgetown University.	Smoking, regular alcohol use, current or past drug abuse, pregnancy, psychiatric illness, and medical instability.
Ornish *et al.*, 1990 [44,45]	Patients with angiographically documented CHD. Age 35-75 years; residence in the greater San Francisco area; no other life-threatening illnesses; no MI during the preceding 6 weeks, and no history of receiving streptokinase or alteplase; not currently receiving lipid-lowering drugs; one, two, or three vessel CHD (defined as any measurable coronary atherosclerosis in a non-dilated or non-bypassed coronary artery); left ventricular ejection fraction > 25%; not scheduled to have coronary artery bypass grafting; and permission granted by patient's cardiologist and primary care physician.	NR. Assume people who do not meet the inclusion criteria.

© 2023 Wang T et al. *JAMA Network Open.*

Author, year	Inclusion criteria	Exclusion criteria
Shah *et al.*, 2018 [46,47]	Age ≥ 18 years; any patient with angiographically defined CHD (≥50% lesion in an artery with ≥ 2 mm caliber); speak English or Spanish.<br><br>Participants have to be ≥ 3 months post-myocardial infarction, coronary artery bypass graft surgery, or infection to minimize confounders of the primary endpoint, hs-CRP concentration.	1. History of an eating disorder; 2. On a vegetarian or vegan diet; 3. Use of steroids or non-steroidal anti-inflammatory medications other than aspirin within 5 half-lives of baseline visit and during the study period; 4. History of a MI or coronary artery bypass graft surgery within the preceding 3 months; 5· Presence of infection within the preceding 3 months; 6. Have a planned staged coronary revascularization or other surgical procedure during study period; 7. History of colon cleansing (for stool microbiome analysis); 8. Use of probiotics or over the counter supplements other than standard vitamins (for stool microbiome analysis); 9. Pregnant; 10. Participating in a competing study; 11. Have any condition (e.g., psychiatric illness) or situation that, in the investigator's opinion, may confound the study results, or may interfere significantly with the patient's ability to adhere with study procedures; 12. Score of > 4 to any of the amotivational items or if the relative autonomy index (defined as average of answers for the 6 autonomous items – average of answers for the 6 controlled items) is ≤ 0 on the Treatment Self-Regulation Questionnaire
Sofi *et al.*, 2018 [48,49]	Being overweight (BMI ≥25 kg/m^2) and the simultaneous presence of ≥ 1 of the following criteria defined by the guidelines for CVD prevention of the European Society of Cardiology: TC levels >190 mg/dL, LDL cholesterol levels >115 mg/dL, TG levels >150 mg/dL, and glucose levels >110 but <126 mg/dL.	People taking medications for any reason, had a serious illness or an unstable condition, were pregnant or nursing, were participating or had participated in a weight loss treatment program in the last 6 months, or were following or had followed a food profile that, to a certain extent, excluded meat, poultry, or fish in the last 6 months.
Tang *et al.*, 2013 [50]	(1) Male ≥ 21 years; (2) BMI 25·0-39·9 kg/m^2; (3) weight stable: <4·5 kg weight change within last 6 months; (4) nonsmoking; (5) constant habitual activity patterns within last 3 months; (6) clinically normal blood profiles (specifically, normal liver and kidney functions; fasting blood glucose <110 mg/dL).	(1) Age: < 21 years; (2) BMI: outside of the 25·0-39·9 kg/m^2 range; (3) Gained or lost > 4·5 kg (10 lbs) within the last 6 months; (4) Body fat <25% as assessed by plethysmography; (5) Smoker (currently or within the last 6 months); (6) Intermittently been involved in a diet and/or exercise program within the last 3 months; (7) Clinically abnormal blood profiles; (8) Taking medications (currently or within the last 3 months) known to influence appetite or metabolism; (9) Clinically diagnosed as diabetic; (10) Allergies to eggs; (11) Lactose intolerance; (12) Clinically diagnosed with diverticulosis; (13) Clinically diagnosed with diverticulitis.
Toobert *et al.*, 2000 [51]	Being a postmenopausal female and having documented CHD defined as atherosclerosis, MI, percutaneous transluminal coronary angioplasty, and/or coronary bypass graft surgery.	Having other life-threatening illnesses, infarction during the preceding 6 weeks; receiving streptokinase or alteplase; or being scheduled for bypass surgery.
Wright *et al.*, 2017 [52]	Age 35-70 and overweight (BMI ≥25 kg/m^2), with a diagnosis of: T2DM, CHD, or the CVD risk factors of hypertension or hypercholesterolaemia.	Diagnoses of life-threatening comorbidities; thyroid disease; coronary artery bypass grafting within 6 weeks; MI within 1 month; angioplasty within 6 months; 450% stenosis of the left main coronary artery; unresponsive congestive heart failure; malignant uncontrolled arrhythmias; homozygous hypercholesterolaemia; severe mental health disorders; current alcohol or drug misuse; currently smoking; currently pregnant or breastfeeding women, prior bariatric surgery, other conditions that directly affect weight (e.g. lead toxicity, malignancy).

ALT, aminotransferase; BMI, body mass index; CVD, cardiovascualr disease; CHD, coronary heart disease; HbA1c, hemoglobin A1c; HDL, high-density lipoprotein cholesterol; LDL, low-density lipoprotein cholesterol; LF, low-fat; MI, myocardial infarction; NAFLD, non-alcoholic fatty liver disease; NR, not reported; SBP, systolic blood pressure; T2DM, type 2 diabetes mellitus; TC, total cholesterol; TG, triglyceride

© 2023 Wang T et al. *JAMA Network Open.*

eTable 4. Study characteristics of ongoing clinical trials that meet our eligibility criteria

Author, year	Country	Trial registration number	Recruitment status	Study arms (target sample size)	Population condition	Diet description		Study design; Intervention duration and follow-up
						Intervention	Comparison	
Fontana *et al.*, 2020 [53]	Australia	ACTRN12620001151921	Not yet recruiting	(1) Intensive lifestyle program (75)<br>(2) AHA diet (75)	CHD	The 5:2 pesco-vegetarian diet: no animal foods other than fish, egg and dairy products; 2 non-consecutive fasting days per week	The AHA diet: general dietary guidelines from the AHA with no energy restriction	1 year, randomized, controlled trial, parallel arm
Kahleova *et al.*, 2019 [54]	USA	NCT04088981	Not yet recruiting	(1) The LF vegan diet (30)<br>(2) The portion-controlled diet (30)	T2DM and BMI 26-40 kg/m^2	The LF, vegan diet: no animal products and added oils; no restriction on energy intake	The portion-controlled diet: participants with an individualized diet plans that reduce daily energy intake by 500 kcal for overweight participants	22 weeks, randomized, controlled trial, crossover design;
Michalsen *et al.*, 2019 [55]	Germany	NCT03901183	Active, not recruiting	(1) The plant-based diet (35)<br>(2) Waiting list (35)	Hypertension and central obesity (WC > 94cm for men and > 80cm for women)	The plant-based diet: detailed diet description not reported	Waiting list: offer equal intervention after the end of study	8 weeks randomized, controlled trial, parallel arm; 8 weeks follow up
Sangeetha *et al.*, 2018	India	CTRI/2018/10/015896	No information	(1) Vegetarian diet<br>(2) Mixed diet	T2DM	Diabetic on Vegetarian diet: T2DM consume the prescribed vegan, pesco-vegetarian and ovo-lacto-vegetarian diet	Diabetics on mixed diet: the prescribed non-vegetarian diet	No information
Turner-Mcgrievy *et al.*, 2017 [56]	USA	NCT03354377	Active, not recruiting	(1) The vegan diet (65)<br>(2) The LF omnivorous diet (65)	BMI 25-49·9 kg/m^2	The vegan diet: a diet consists of whole grains, fruits, vegetables, and legumes; supplemented by the Oldways African Heritage and Health program	The LF omnivorous diet: supplemented by the Oldways African Heritage and Health program, which includes a food pyramid guide	2 years randomized, controlled trial, parallel arm
Wright *et al.*, 2017 [57]	New Zealand	ACTRN12617000541303	Results not published yet	(1) Whole foods plant-based diet (24)<br>(2) Usual care (24)	Obese (BMI ≥ 30 kg/m^2) and T2DM/prediabetes	Whole foods plant-based approach: avoid animal products, including meat, dairy and eggs; no energy restriction; daily 50mcg Vit B12 supplement	Usual care	10 weeks randomized, controlled trial, parallel arm; 42 weeks follow up

AHA, American Heart Associations; BMI, body mass index; CHD, coronary heart disease; HbA1c, hemoglobin A1c; HDL, high-density lipoprotein cholesterol; LDL, low-density lipoprotein cholesterol; LF, low-fat; MED, Mediterranean diet; T2DM, type 2 diabetes mellitus; TC, total cholesterol; TG, triglyceride; WC, waist circumference.

© 2023 Wang T et al. *JAMA Network Open.*

eTable 5. Participant characteristics and study description of included studies

Author, year	Race/Ethnicity	Physical activity description	Medication		
			General description	Baseline	EOI
Aldana *et al.*, 2006 [24]	NR	(1) An exercise physiologist prescribed an individualized exercise program.<br>**Stage 1** (intensive 12-week): participants attended two 4-hour sessions/week, including supervised exercise, stress management, a meal, lifestyle-related lecture, and group support.<br>**Stage 2:** one 4-hour session/week with similar content.<br>**Stage 3:** an alumni-based community of previous program participants.<br>(2) The five main components of traditional cardiac rehabilitation: medical evaluation, prescribed exercise, cardiac risk factor modification, education, counselling.	NI	NI	There were no differences in medication use between groups across time.
Barnard *et al.*, 2006 [25-28]	(1)<br>Black, non-Hispanic: 22<br>White, non-Hispanic: 21<br>White, Hispanic: 4<br>Asian, non-Hispanic: 2<br>(2)<br>Black, non-Hispanic: 22<br>White, non-Hispanic: 22<br>White, Hispanic: 2<br>Asian, non-Hispanic: 4<br><br>p-value: 0·71	Participants were asked not to alter their exercise habits during the intervention period.	Continue preexisting medication, except when hypoglycemia*	Insulin:<br>(1) 11 (2) 5 p=0·09<br>Metformin:<br>(1) 34 (2) 39 p=0·33<br>Sulfonylurea:<br>(1) 25 (2) 29 p=0·49<br>Thiazolidinedione:<br>(1) 16 (2) 15 p=0·78<br>Other diabetes medications:<br>(1) 1 (2) 2 p=0·57<br>Blood pressure medications:<br>(1) 31 (2) 38 p=0·17<br>Lipid-lowering medications:<br>(1) 27 (2) 27 p=0·88	↓ diabetes medication, mainly as necessitated by hypoglycemia:<br>(1) 43%<br>(2) 26%<br><br>↑ medications without investigators' authorization:<br>(1) 8%<br>(2) 8%
Barnard *et al.*, 2018 [29]	(1)<br>White non-Hispanic: 13<br>Black non-Hispanic: 7<br>White, Hispanic: 1<br>Black, Hispanic: 0<br>Asian, non-Hispanic: 1<br>(2)<br>White, non-Hispanic: 7<br>Black, non-Hispanic: 13<br>White, Hispanic: 1<br>Black, Hispanic: 1<br>Asian, non-Hispanic: 1	All participants were asked not to alter their exercise patterns.	All participants were asked not to change their use of medications or nutritional supplements, except as recommended by their personal physicians.	NR	On glycemic control medication:<br>(1) 4 ↓, 2 mixed (changes in opposite directions)<br>(2) 6 ↓, 3 ↑, 4 mixed<br><br>On lipid-lowering medications: reported "several participants altered".

© 2023 Wang T et al. *JAMA Network Open.*

Author, year	Race/Ethnicity	Physical activity description	Medication		
			General description	Baseline	EOI
Barnard *et al.*, 2021 [30]	(1)<br>White: 16<br>Black: 14<br>Asian, Pacific Islander: 0<br>American Indian, Eskimo, Aleut: 0<br>Did not disclose:0<br>(2)<br>White: 15<br>Black: 16<br>Asian, Pacific Islander: 0<br>American Indian, Eskimo, Aleut: 1<br>Did not disclose:0<br><br>p-value: 0·90	All study participants were asked not to alter their exercise habits, except as modified by ther personal physicians.	All study participants were asked to continue their preexisting medication regimens for the study duration, except as modified by their personal physicians.	Lipid-lowering therapy:<br>(1) 11<br>(2) 12<br>p-value: 0·95<br><br>Antihypertensive therapy:<br>(1) 14<br>(2) 16<br>p-value: 0·79<br><br>Thyroid medications:<br>(1) 1<br>(2) 3<br>p-value: 0·61	Lipid-lowering therapy:<br>(1) 7 ↓ or discontinued<br>(2) 2 ↓ or discontinued, 1 ↑<br><br>Antihypertensive therapy:<br>(1) 7 ↓ or discontinued, 1 ↑<br>(2) 6 ↓ or discontinued, 1 ↑
Bunner *et al.*, 2015 [31]	(1)<br>Black: 5<br>Non-black: 12<br>(2)<br>Black: 11<br>Non-black: 6<br><br>p-value: 0·09	NI	Participants were asked to keep their diabetes medications constant when possible, but to follow the advice of their personal physicians regarding medication use.	Medications for diabetic neuropathy symptoms:<br>(1) 5<br>(2) 5<br><br>Metformin:<br>(1) 12<br>(2) 12<br>p-value: 0·99<br><br>Insulin:<br>(1) 6<br>(2) 9<br>p-value: 0·30<br><br>On other agents:<br>(1) 10<br>(2) 7<br>p-value: 0·31	Medications for diabetic neuropathy symptoms:<br>(1) 2 began, 2 eliminated<br>(2) 1 began and then stopped taking pregblin<br><br>Glucose-lowering medications:<br>(1) 10 ↓, 2 ↑<br>(2) 1 ↓, 2 ↑<br><br>Lipid-lowering medications:<br>(1) 4 ↓, 1 ↑<br>(2) 0 ↓, 3 ↑<br><br>Blood pressure medications:<br>(1) 2 ↓, 4 ↑<br>(2) 0 ↓, 1 ↑

© 2023 Wang T et al. *JAMA Network Open.*

Author, year	Race/Ethnicity	Physical activity description	Medication		
			General description	Baseline	EOI
Burke *et al.*, 2006 [32-34]	White: n (%)<br>(1) 59 (70·2)<br>(2) 69 (70·4)<br><br>p-value: 0·98	Instruct to engage in exercise (walking ≥ 150 min/wk). Walking was recommended as the primary form of structured activity and participants were encouraged to increase daily activities. Four group sessions focused on exercise, e.g., aerobic training, weights. Study personnel monitored and provided feedback to subjects regarding their weekly recorded activity.	NI	Lipid-lowering drugs:<br>(1) SBT-LOV: 8<br>(2) SBT: 2	3 subjects of the LOV group and 1 of the SBT group no longer on drugs; 1 in each group initiated drugs for hyperlipidemia.
Garousi *et al.*, 2021 [35]	NR	Patients were instructed to follow their routine physical activity regime.	Instruct not to consume supplements and/or medication that affect outcomes of interest.	NI	NI
Kahleova *et al.*, 2010 [36]	Caucasian-White	Participants were asked not to alter their exercise habits during the first 12 weeks.<br><br>During weeks 13–24, they were prescribed an individualized exercise programme. Participants exercised at 60% of their maximal heart rate twice a week for 1 h under professional supervision, plus once a week at home or at the sports centre with the same intensity; they were given a sport-tester Polar FT4 (Polar, Kempele, Finnland) and a pedometer (Omron HJ-113, Omron, Kyoto, Japan) for individual physical activities and were repeatedly instructed on how to use them.	Ask to continue pre-existing medication regimens, except when hypoglycaemia* occurred repeatedly	Oral hypoglycaemic agents:<br>- Metformin:<br>(1) 29 (2) 28<br>- Sulphonylurea:<br>(1) 20 (2) 13<br>- Thiazolidinedione:<br>(1) 7 (2) 5<br>- Other:<br>(1) 8 (2) 3<br>Lipid-lowering therapy:<br>(1) 22 (2) 16<br>Antihypertensive therapy:<br>(1) 25 (2) 22	Diabetes medication:<br>(1) 43% ↓ (2) 5% ↓<br><br>p-value < 0·001, difference between groups: 38% (95% CI 17, 58).
Kahleova *et al.*, 2020 [37]	(1) White: 57; Black: 60; Asian, Pacific Islander: 1; American Indian, Eskimo, Aleut: 2; Did not disclose: 2<br>(2) White: 60; Black: 53; Asian, Pacific Islander: 7; American Indian, Eskimo, Aleut: 0; Did not disclose: 2<br><br>p-value: 0·06	All participants were asked to maintain their customary exercise habits and medications unless modified by their personal physicians.	All participants were asked to maintain their customary medications unless modified by their personal physicians.	Lipid-lowering therapy:<br>(1) 22<br>(2) 21<br><br>Antihypertensive therapy:<br>(1) 33<br>(2) 31<br><br>Thyroid medications:<br>(1) 16<br>(2) 12<br>p-value: 0·42	Minimal medication changed (reported by corresponding author)

© 2023 Wang T et al. *JAMA Network Open.*

Author, year	Race/Ethnicity	Physical activity description	Medication		
			General description	Baseline	EOI
Lee *et al.*, 2016 [38]	Korean	The participants were asked to maintain the usual level of physical activity, and to not modify their exercise habits during the intervention period.	Ask to maintain current medication, without any control of the dose or type of medication for 12 weeks; however, dose reduction was permitted when it was necessary according to a physician's judgment.	Oral hypoglycaemic agents:<br>- Insulin:<br>(1) 7 (2) 8<br>p-value = 0·813<br>- Metformin:<br>(1) 34 (2) 36<br>p-value = 0·764<br>- Sulphonylurea:<br>(1) 17 (2) 21<br>p-value = 0·449<br>- Other:<br>(1) 14 (2) 19<br>p-value = 0·314<br><br>Lipid-lowering therapy:<br>(1) 23 (2) 26<br>p-value = 0·608<br><br>Antihypertensive therapy:<br>(1) 18 (2) 22<br>p-value = 0·455	None of the participants changed their medication dose over the 12 weeks.
Liao *et al.*, 2007 [39]	NR	NI	NI	NI	NI
Mahon *et al.*, 2007 [40]	All caucasian	Subjects were instructed to maintain their habitual activities and to not begin any type of structured exercise program during the study.	Instruct to stop all nutritional supplements (except calcium), nonprescription medication and alcohol from 3 weeks before till the end of study.	NI	NI
Mishra *et al.*, 2013 [41,42]	(1) White: 88; Black: 34; Asian: 10; Other: 10<br>(2) White: 98; Black: 41; Asian: 5; Other: 6<br><br>p-value: 0·3	All participants were asked not to alter their exercise patterns during the study period.	Ask to continue pre-existing medication regimens unless modified by their personal physicians.	NI	NI

© 2023 Wang T et al. *JAMA Network Open.*

Author, year	Race/Ethnicity	Physical activity description	Medication		
			General description	Baseline	EOI
Nicholson *et al.*, 1999 [43]	NR	No exercise recommendation were made.	Subjects met with the medical director or nurse-project coordinator every 2 weeks. Medication needs were assessed at these visits and as needed between visits, according to an established protocol.	Oral hypoglycaemic agents:<br>(1) 6 (2) 4<br><br>Insulin:<br>(1) 2 (2) 0<br><br>Antihypertensive therapy:<br>(1) 5 (2) 4<br><br>Lipid-lowering medications:<br>(1) 3 (2) 1	Oral hypoglycaemic agents:<br>(1) 1 stopped, 3↓<br>(2) 4 remain same dosage<br><br>Insulin:<br>(1) 2 ↓ (2) 0<br><br>Antihypertensive therapy:<br>(1) 2 discontinued<br>(2) 1 stopped<br><br>No changes in lipid-lowering drugs.
Ornish *et al.*, 1990 [44,45]	NR	Individually prescribed exercise (typically walking) according to baseline treadmill test results. Ask to exercise for ≥ 3 h/week and to spend ≥ 30 min/session within target heart rates.	NR	NR	NR
Shah *et al.*, 2018 [46,47]	(1)<br>White: 46<br>Black: 1<br>Asian: 3<br>Other: 0<br>(2)<br>White: 40<br>Black: 6<br>Asian: 3<br>Other: 1<br><br>p-value: 0·17	No instruction was given on exercise.	Participants were randomized at least 7 days after invasive coronary angiography so that medication regimens would be as stable as possible during the study period. All participants are encouraged and expected to continue to take any medication his or her physician has prescribed.	Aspirin:<br>(1) 46 (2) 48 p=0·68<br>Any P2Y12 inhibitor:<br>(1) 43 (2) 42 p=0·99<br>Statin:<br>(1) 47 (2) 48 p=0·99<br>beta-Blocker:<br>(1) 33 (2) 31 p=0·84<br>Calcium channel blocker:<br>(1) 10 (2) 8 p=0·80<br>Angiotensin-converting enzyme inhibitor or angiotensin receptor blocker:<br>(1) 28 (2) 26 p=0·84<br>Long-acting nitrate:<br>(1) 2 (2) 2 p=0·99<br>Ranolazine:<br>(1) 1 (2) 3 p=0·62	NI

© 2023 Wang T et al. *JAMA Network Open.*

Author, year	Race/Ethnicity	Physical activity description	Medication		
			General description	Baseline	EOI
Sofi *et al.*, 2018 [48,49]	NR	Participants were instructed not to alter their lifestyle and exercise habits during the study, and no weight loss goal was given.	Participants were excluded if they were taking medications.	NA	NA
Tang *et al.*, 2013 [50]	42 Caucasian (98%)<br>1 African American (2%)	NI	NI	NI	NI
Toobert *et al.*, 2000 [51]	White, not Latino:<br>(1) 13<br>(2) 10<br>Lafino:<br>(1) 0<br>(2) 1<br>Native American or Alaskan:<br>(1) 1<br>(2) 0<br><br>p-value: 0·57	Daily group physical activity sessions included warm-up, walking or aerobics, and a cool-down led by an American College of Sports Medicine certified exercise physiologist. Participants were individually prescribed exercise intensity based on their treadmill exercise test performance. Following the retreat, the intervention exercise program required participants to engage in a 1-hour session per day at least 3 days each week.	NR	Lipid-lowering (n)<br>(1) 4 (2) 5<br>p-value: 0·65<br><br>Lipid-lowering (mg)<br>(1) 95 (318)<br>(2) 175 (539)<br><br>Blood pressure-lowering (n)<br>(1) 10 (2) 9<br>p-value: 0·73<br><br>Antihypertensive (mg)<br>(1) 97 (121)<br>(2) 120 (115)<br><br>Estrogen replacement therapy (n)<br>(1) 5 (2) 5<br>p-value: 0·64	Lipid-lowering (mg)<br>(1) 101 (340)<br>(2) 126 (357)<br>p-value group x time: 0·140<br><br>Antihypertensive (mg)<br>(1) 92 (207)<br>(2) 262 (354)<br>p-value group x time: 0·083
Wright *et al.*, 2017 [52]	NR	No instruction was given on exercise.	NA	(1) 94<br>(2) 74	(1) 74, 21% ↓<br>(2) 80, 8% ↑

*Hypoglycemia: fasting plasma glucose < 4·4 mmol/l or hypoglycemic symptoms accompanied by a capillary glucose reading < 3·5 mmol/l. In such cases, medications were reduced by a study endocrinologist following the medication protocol.
LF, low-fat diet; NA, not applicable; NI, no information; NR, not reported; PN, probably no.

© 2023 Wang T et al. *JAMA Network Open.*

eTable 6. Dietary data of included studies

Author, year	Dietary assessment method	Energy intake	Saturated fat intake	Trans fatty acid intake	Total cholesterol intake	Dietary fibre intake	Alcohol intake
Aldana *et al.*, 2006 [24]	Self-reported, no detailed information	NR	NR	NR	NR	NR	NR
Barnard *et al.*, 2006 [25-28]	3-d WFR (2 weekdays + 1 weekend day, using a food scale, after participants Had completed a 3-day practice record)	Mean change (kcal):<br>(1) -313.2 ± 411.6<br>(2) -385.9 ± 498.5<br><br>Change between group:<br>72.8 (95%CI: -109.9, 255.3)<br><br>p-value: 0·43	Mean change (g):<br>(1) -16.7 ± 10.9<br>(2) -6.9 ± 10.8<br><br>Change between group:<br>-9.8 (95%CI: -14.1, -5.5)<br><br>p-value < 0.0001	Mean change (g):<br>(1) -2.9 ± 2.9<br>(2) -1.3 ± 2.5<br><br>Change between group:<br>-1.5 (95%CI: -2.6, -0.4)<br><br>p-value: 0.007	Mean change (mg):<br>(1) -250.3 ± 221.9<br>(2) -107.6 ± 167.5<br><br>Change between group:<br>-142.7 (95%CI: -221.0, -64.4)<br><br>p-value: 0.001	Mean change (g):<br>(1) 16.4 ± 12.4<br>(2) -0.4 ± 7.4<br><br>Change between group:<br>16.9 (95%CI: 12.8, 20.9)<br><br>p-value < 0.0001	NR
Barnard *et al.*, 2018 [29]	3-d food record (instructed portion sizes estimation using food models, along with a sample record)	Mean change (kcal):<br>(1) -204 ± 95<br>(2) -306 ± 100<br><br>p-value between group: 0·4612	Mean change (g):<br>(1) -14 ± 2<br>(2) -7 ± 2<br><br>p-value: 0.0341	NR	Mean change (mg):<br>(1) -169 ± 16<br>(2) -33 ± 34<br><br>p-value: 0.0012	NR	NR
Barnard *et al.*, 2021 [30]	3-d food record, collected and analyzed by an RD or a staff member certified in Nutrition Data System for Research version 2018	Mean change (kcal):<br>(1) -500 ± 327.99<br>(2) 79 ± 511.92<br><br>Change between group:<br>-579 (95%CI: -801, -357)<br><br>p-value <0.001	Mean change (% of energy):<br>(1) -8<br>(95%CI: −9, −6)<br>(2) -1<br>(95%CI: −2, 0)<br><br>Change between group:<br>-6 (95%CI: -8, -5)<br><br>p-value < 0.001	NR	Mean change (mg):<br>(1) -273 (95%CI: -317, -230)<br>(2) -25 (95%CI: -93, 43)<br><br>Change between group:<br>-248 (95%CI: -331, -166)<br><br>p-value < 0.001	Mean change (g):<br>(1) 10 (95%CI: 8, 13)<br>(2) 5 (95%CI: 2, 7)<br><br>Change between group:<br>6 (95%CI: 2, 9)<br><br>p-value < 0.001	Mean change (g):<br>(1) -2 (95%CI: -4, 0)<br>(2) 0 (95%CI: -3, 3)<br><br>Change between group:<br>-2 (95%CI: -5, 2)<br><br>p-value: 0.31
Bunner *et al.*, 2015 [31]	2-d dietary record	NR	NR	NR	NR	NR	NR

(1), vegetarian diet intervention group; (2), non-vegetarian diet comparison group; EOI, end of intervention; LF, low-fat diet; FHQ, Food Habits Questionnaire; NA, not applicable; NI, no information; NR, not reported; PN, probably no; RD, Registered Dietitian; SFA, saturated fatty acid; TC, total cholesterol; TFA, saturated fatty acid; WFR, weighed food records

© 2023 Wang T et al. *JAMA Network Open.*

eTable 6. Continued

Author, year	Dietary assessment method	Energy intake	Saturated fat intake	Trans fatty acid intake	Total cholesterol intake	Dietary fibre intake	Alcohol intake
Burke *et al.*, 2006 [32-34]	3-d food record (2 work days + one leisure day, reviewed by trained research staff for completeness and clarity)	Mean change (kcal):<br>(1) -535.98 ± 511.81<br>(2) -519.80 ± 533.15<br><br>p-value between group: 0.836	Mean change (% of energy):<br>(1) -3.76 ± 3.75<br>(2) -3.12 ± 3.86<br><br>p-value between group: 0.17	NR	NR	NR	NR
Garousi *et al.*, 2021 [35]	3-d WFR (2 weekdays and 1 weekend day; food models and national food composition tables were used)	EOI Energy intake (kcal):<br>(1) 1911.02 ± 290.04<br>(2) 2075.54 ± 314.68<br><br>p-value between group: 0.061	EOI SFA intake (g):<br>(1) 18.38 ± 3.11<br>(2) 21.69 ± 4.46<br><br>p-value between group: 0.004	NR	EOI TC intake (mg):<br>(1) 298 ± 133<br>(2) 264 ± 122<br><br>p-value between group: 0.397	EOI Dietary fibre intake (g):<br>(1) 42.32 ± 7.02<br>(2) 27.56 ± 6.31<br><br>p-value between group < 0.001	NR
Kahleova *et al.*, 2010 [36]	3-d food record (2 weekdays and 1 weekend day)	Mean change (kcal):<br>(1) -137 ± 477<br>(2) -128 ± 641	NR	NR	Mean change (mg):<br>(1) -322 ± 224<br>(2) -55.6 ± 187	Mean change (mg):<br>(1) 6.02 ± 9.58<br>(2) 1.73 ± 9.89	NR
Kahleova *et al.*, 2020 [37]	3-d food record (collected and analyzed by staff members certified in the Nutrition Data System for Research)	Mean change (kcal):<br>(1) -490.7 ± 646.8<br>(2) -135.8 ± 603.8<br><br>Change between group:<br>-354.9 (95%CI: -519.0, -190.8)<br><br>p-value <0.001	Mean change (g):<br>(1) -18.6 (95%CI: -20.7, -16.5)<br>(2) -2.4 (95%CI: -4.9, 0.1)<br><br>Change between group:<br>-16.2 (95%CI: -19.4, -13.0)<br><br>p-value <0.001	NR	Mean change (mg):<br>(1) -233.1 (95%CI: -259.4, -206.8)<br>(2) -14 (95%CI: -51.7, 23.7)<br><br>Change between group:<br>-219.1 (95%CI: -264.9, -173.3)<br><br>p-value <0.001	Mean change (g):<br>(1) 10.6 (95%CI: 7.8, 13.3)<br>(2) -0.56 (95%CI: -2.6, 1.5)<br><br>Change between group:<br>11.1 (95%CI: 7.8, 14.5)<br><br>p-value <0.001	NR
Lee *et al.*, 2016 [38]	A dietitian conducted 24-h recalls (4 times/ month, including 3 weekdays + once on a weekend); baseline not assessed.	EOI Energy intake (kcal):<br>(1) 1496.2 ± 104.8<br>(2) 1559.7 ± 181.6<br><br>p-value between group: 0.042	EOI SFA intake (g):<br>(1) 3.2±1.5<br>(2) 6.7±2.7<br><br>p-value between group <0.001	NR	EOI TC intake (mg):<br>(1) 70.3±57.4<br>(2) 240.7±74.7<br><br>p-value between group <0.001	EOI Dietary fibre intake (g):<br>(1) 33.7 ± 4.8<br>(2) 24.9 ± 4.5<br><br>p-value between group <0.001	NR

(1), vegetarian diet intervention group; (2), non-vegetarian diet comparison group; EOI, end of intervention; LF, low-fat diet; FHQ, Food Habits Questionnaire; NA, not applicable; NI, no information; NR, not reported; PN, probably no; RD, Registered Dietitian; SFA, saturated fatty acid; TC, total cholesterol; TFA, saturated fatty acid; WFR, weighed food records

© 2023 Wang T et al. *JAMA Network Open.*

eTable 6. Continued

Author, year	Dietary assessment method	Energy intake	Saturated fat intake	Trans fatty acid intake	Total cholesterol intake	Dietary fibre intake	Alcohol intake
Liao *et al.*, 2007 [39]	Daily diet record	EOI Energy intake (kcal):<br>(1) 1156 ± 165<br>(2) 1229 ± 15	NR	NR	NR	EOI Dietary fibre intake (g):<br>(1) 6.6±0.9<br>(2) 7.6±1.1	NR
Mahon *et al.*, 2007 [40]	3-d food record	EOI Energy intake (kcal):<br>(1) 1158 ± 341<br>(2) 1570 ± 633<br><br>p-value <0.05	NR	NR	NR	EOI Dietary fibre intake (g):<br>(1) 18 ± 8<br>(2) 14 ± 8	NR
Mishra *et al.*, 2013 [41,42]	24-h recall over two 24-h periods, using an online program (ASA24: Automated Self-Administered 24-hour Recall)	Mean change (kcal):<br>(1) -264.6 ± 65.6<br>(2) -36.8 ± 58<br><br>Change between group:<br>-130 (95%CI: -281, 20.4)<br><br>p-value: 0.09	Mean change (% of energy):<br>(1) -2.8 ± 0.4<br>(2) -0.36 ± 0.45	NR	Mean change (mg):<br>(1) -107.7 ± 21.9<br>(2) -13.9 ± 19.6<br><br>Change between group:<br>-92 (95%CI: -141.5, -42.6)<br><br>p-value <0.001	Mean change (g):<br>(1) 3.5 ± 1.3<br>(2) 0.74 ± 0.72	NR
Nicholson *et al.*, 1999 [43]	3-d food record (2 weekdays and 1 weekend day)	Mean change (kcal):<br>(1) -274 ± 464.1<br>(2) 96 ± 339.4	EOI SFA intake (% of energy):<br>(1) 3 ± 2.0<br>(2) 8 ± 1.8	NR	EOI TC intake (mg):<br>(1) 4.4 ± 7.4<br>(2) 122 ± 31	EOI Dietary fibre intake (g):<br>(1) 26±8.2<br>(2) 20±2.7	NR
Ornish *et al.*, 1990 [44,45]	3-d food record	Energy intake after intervention (MJ):<br>(1) 7.6 ± 2.1<br>(2) 7.1 ± 1.9<br><br>p-value: 0.5082	NR	NR	TC intake after intervention (mg):<br>(1) 12.4 ± 45.8<br>(2) 190 ± 99<br><br>p-value < 0.0001	NR	NR
Shah *et al.*, 2018 [46,47]	4-day WFR (with measuring cups and spoons); RD calls the patient 2/week on random days to obtain 24-h recalls	EOI Energy intake (kcal: Median [IQR]):<br>(1) 1715 [1482–2052]<br>(2) 1531 [1197–1817]<br><br>p-value: 0.01	EOI SFA intake (g: Median [IQR]):<br>(1) 9 [6–12]<br>(2) 10 [8–15]<br><br>p-value: 0.03	EOI TFA intake (g: Median [IQR]):<br>(1) 0.06 [0.00–0.74]<br>(2) 0.14 [0.05–0.43]<br><br>p-value: 0.4	EOI TC intake (mg: Median [IQR]):<br>(1) 0 [0–2]<br>(2) 142 [110–240]<br><br>p-value < 0.001	EOI Dietary fibre intake (g: Median [IQR]):<br>(1) 36 [30–43]<br>(2) 25 [20–30]<br><br>p-value < 0.001	NR

(1), vegetarian diet intervention group; (2), non-vegetarian diet comparison group; EOI, end of intervention; LF, low-fat diet; FHQ, Food Habits Questionnaire; NA, not applicable; NI, no information; NR, not reported; PN, probably no; RD, Registered Dietitian; SFA, saturated fatty acid; TC, total cholesterol; TFA, saturated fatty acid; WFR, weighed food records

© 2023 Wang T et al. *JAMA Network Open.*

eTable 6. Continued

Author, year	Dietary assessment method	Energy intake	Saturated fat intake	Trans fatty acid intake	Total cholesterol intake	Dietary fibre intake	Alcohol intake
Sofi *et al.*, 2018 [48,49]	3-d food record (2 weekdays and 1 weekend day)	Mean change (kcal):<br>(1) -586.3 ± 486.9<br>(2) 496.7 ± 539.8<br><br>p-value between group: 0.18	Mean change (% of energy):<br>(1) -0.31 ± 2.2<br>(2) -1.0 ± 3.3<br><br>p-value between group: 0.41	NR	Mean change (% of energy):<br>(1) -105.6 ± 91.5<br>(2) -49.7 ± 121.8<br><br>p-value between group: 0.001	NR	NR
Tang *et al.*, 2013 [50]	Daily food check-offs noting any foods not consumed and extra foods consumed	EOI Energy intake (kcal):<br>(1) 2273 ± 78<br>(2) 2254 ± 75	NR	NR	NR	NR	NR
Toobert *et al.*, 2000 [51]	The Kristal FHQ, a 4-d food record, and a paper-and-pencil dietary fat intake screener	EOI Energy intake (kcal):<br>(1) 1349 ± 284<br>(2) 1394 ± 477<br><br>p-value: 0·164	EOI SFA (% of energy):<br>(1) 3.1 ± 2.4<br>(2) 8.7 ± 3.0<br><br>p-value between group < 0.000	NR	NR	NR	NR
Wright *et al.*, 2017 [52]	3-d diet recall to track dietary indiscretions	NR	NR	NR	NR	NR	NR

(1), vegetarian diet intervention group; (2), non-vegetarian diet comparison group; EOI, end of intervention; LF, low-fat diet; FHQ, Food Habits Questionnaire; NA, not applicable; NI, no information; NR, not reported; PN, probably no; RD, Registered Dietitian; SFA, saturated fatty acid; TC, total cholesterol; TFA, saturated fatty acid; WFR, weighed food records

eTable 7. Detailed risk of bias assessment of included studies

**Author, year**	Aldana, 2006 [24]	Barnard, 2006 [25]	Barnard, 2018 [29]	Barnard, 2021 [30]	Bunner, 2015 [31]	Burke, 2006 [32]	Garousi, 2021 [35]	Kahleova, 2010 [36]	Kahleova, 2020 [37]	Lee, 2016 [38]	Liao, 2007 [39]	Mahon, 2007 [40]	Mishra, 2013 [41]	Nicholson, 1999 [43]	Ornish, 1990 [44]	Shah, 2018 [46]	Sofi, 2018 [48]	Tang, 2013 [50]	Toobert, 2000 [51]	Wright, 2017 [52]
**Domain 1: Bias arising from the randomisation process**																				
**1.1**	NI	Y	Y	Y	Y	PN	PY	Y	Y	Y	NI	Y	Y	Y	NI	Y	Y	NI	NI	Y
**1.2**	NI	PY	Y	Y	Y	NI	NI	Y	Y	NI	NI	NI	NI	Y	Y	Y	Y	NI	NI	PY
**1.3**	N	N	N	N	PY	N	Y	PN	PN	N	PN	PN	PN	Y	PY	N	N	N	N	PN
**ROB**	SC	L	L	L	SC	SC	H	L	L	SC	SC	SC	SC	SC	SC	L	L	SC	SC	L
**Domain 2: Bias due to deviations from intended intervention**																				
**2.1**	PY	Y	PY	Y	Y	Y	PY	Y	Y	Y	PY	Y	PY	PY	Y	PY	Y	PY	Y	Y
**2.2**	PY	Y	PY	Y	Y	PY	PY	PY	Y	PY	PY	PY	PY	PY	PY	PY	Y	PY	PY	PY
**2.3**	NI	PN	PN	PN	N	NI	NI	NI	N	NI	PN	NI	PN	NI	PN	NI	NI	NI	NI	PN
**2.4**	NA	NA	NA	PN	NA	NA	NA	NA	NA	NA	NA	NA	NA	NA	NA	NA	NA	NA	NA	NA
**2.5**	NA	NA	NA	NA	NA	NA	NA	NA	NA	NA	NA	NA	NA	NA	NA	NA	NA	NA	NA	NA
**2.6**	Y	Y	PN	Y	Y	Y	N	Y	Y	Y	Y	PN	Y	Y	Y	Y	Y	PN	Y	PY
**2.7**	NA	NA	PY	NA	NA	NA	N	NA	NA	NA	NA	PN	NA	NA	NA	NA	NA	PN	NA	NA
**ROB**	SC	L	H	L	L	SC	SC	SC	L	SC	L	SC	L	SC	L	SC	SC	SC	SC	L
**Domain 3: Bias due to missing outcome data**																				
**3.1**	N	Y	N	N	Y	N	Y	N	N	N	Y	PN	Y	N	N	Y	Y	Y	N	Y
**3.2**	No	NA	N	N	NA	Y	NA	Y	Y	Y	NA	N	NA	N	PN	NA	NA	NA	PN	NA
**3.3**	Y	NA	PN	PY	NA	NA	NA	NA	NA	NA	NA	Y	NA	PY	PN	NA	NA	NA	PY	NA
**3.4**	PY	NA	NA	PY	NA	NA	NA	NA	NA	NA	NA	NI	NA	PY	NA	NA	NA	NA	PY	NA
**ROB**	H	L	L	H	L	L	L	L	L	L	L	H	L	SC	L	L	L	L	H	L

H, high; L, low; N, no; NA, not applicable; NI, no information; PN, probably no; PY, probably yes; Ref, references; ROB, risk of bias; SC, some concerns; Y, yes

© 2023 Wang T et al. *JAMA Network Open.*

**Author, year**	Aldana, 2006 [24]	Barnard, 2006 [25]	Barnard, 2018 [29]	Barnard, 2021 [30]	Bunner, 2015 [31]	Burke, 2006 [32]	Garousi, 2021 [35]	Kahleova, 2010 [36]	Kahleova, 2020 [37]	Lee, 2016 [38]	Liao, 2007 [39]	Mahon, 2007 [40]	Mishra, 2013 [41]	Nicholson, 1999 [43]	Ornish, 1990 [44]	Shah, 2018 [46]	Sofi, 2018 [48]	Tang, 2013 [50]	Toobert, 2000 [51]	Wright, 2017 [52]
**Domain 4: Bias in measurement of the outcome**																				
**4.1**	NI	N	N	PN	N	N	N	N	N	N	N	N	N	N	N	N	N	N	N	N
**4.2**	PN	N	N	N	PN	N	PN	N	PN	PN	PN	N	N	PN	N	N	N	N	PN	N
**4.3**	NI	N	N	NI	NI	NI	NI	N	PN	PN	NI	NI	NI	NI	N	N	N	NI	NI	Y
**4.4**	PN	NA	NA	PN	PN	PN	PN	NA	NA	NA	PN	PN	PN	PN	NA	NA	NA	PN	PN	PN
**4.5**	NA	NA	NA	NA	NA	NA	NA	NA	NA	NA	NA	NA	NA	NA	NA	NA	NA	NA	NA	NA
**ROB**	L	L	L	L	L	L	L	L	L	L	L	L	L	L	L	L	L	L	L	L
**Domain 5: Bias in selection of the reported result**																				
**5.1**	PN	Y	PY	N	NI	PN	PY	PY	PY	PY	PY	PN	PY	PY	PY	Y	Y	NI	PY	PY
**5.2**	PN	N	N	N	N	Y	PY	N	N	N	PN	N	N	Y	N	Y	N	PN	PN	N
**5.3**	PY	N	PY	PN	N	N	PN	N	PN	PN	PN	PN	N	PN	N	PN	Y	PN	PN	N
**ROB**	H	L	H	SC	SC	H	H	L	L	L	L	SC	L	H	L	H	H	SC	L	L
**Over-all ROB**	H	L	H	H	SC	H	H	L	SC	SC	SC	H	SC	H	SC	H	H	H	H	L

H, high; L, low; N, no; NA, not applicable; NI, no information; PN, probably no; PY, probably yes; Ref, references; ROB, risk of bias; SC, some concerns; Y, yes

© 2023 Wang T et al. *JAMA Network Open.*

eTable 8. GRADE assessment of the certainty of evidence in people with CVD or people at high risk of CVD

Certainty assessment							№ of patients		Effect		
№ of studies	Study design	Risk of bias	Inconsistency	Indirectness	Imprecision	Other considerations	Vegetarian dietary patterns	Other intervention diets or the usual diet	MD (95% CI)	Certainty	Importance
**Low-density lipoprotein cholesterol (follow-up: range 8 weeks to 2 years)**											
19	RCTs	not serious[a]	serious[b]	not serious	not serious	none	827	834	**-6·6 mg/dL** (-10·1, -3.1)	⨁⨁⨁◯ Moderate	Important
**Hemoglobin A1c (follow-up: range 8 weeks to 6 months)**											
10	RCTs	not serious[c]	serious[d]	not serious	not serious	none	393	385	**-0·24 %** (-0·40, -0·07)	⨁⨁⨁◯ Moderate	Important
**Systolic blood pressure (follow-up: range 8 weeks to 2 years)**											
14	RCTs	not serious[e]	serious[f]	not serious	serious[g]	none	472	483	**-0·1 mmHg** (-2.8, 2·6)	⨁⨁◯◯ Low	Important
**Body weight (follow-up: range 8 weeks to 1 years)**											
16	RCTs	not serious[h]	very serious[i]	not serious	not serious	none	695	700	**-3·4 kg** (-4·9, -2·0)	⨁⨁◯◯ Low	Important

CI: confidence interval; CVD: cardiovascular disease; MD: mean difference; RCT: randomised controlled trial

a. All included studies lack blinding from patients and probably from caregivers, given it is impossible to blind people from a dietary intervention; nine out of 11 included studies did not provide enough information to judge allocation concealment; four out of 19 included studies did not follow intention-to-treat principle in data analysis; six out of 19 included studies were at risk of selective reporting.
b. I^2=67% and tau-square=37·59 indicate a substantial heterogeneity.
c. All included studies lack blinding from patients and probably from caregivers, given it's impossible to blind people from a dietary intervention; two out of ten included studies did not provide enough information to judge allocation concealment; all studies employed intention-to-treat principle in data analysis; two out of ten included studies were at risk of selective reporting.
d. I^2=88% and tau-square=0·058 indicate a considerable heterogeneity.
e. All included studies lack blinding from patients and probably from caregivers, given it's impossible to blind people from a dietary intervention; seven out of 14 included studies did not provide enough information to judge allocation concealment; three out of 14 included studies did not follow intention-to-treat principle in data analysis; four out of 14 included studies were at risk of selective reporting.
f. I^2=65% and tau-square=14·25 indicate a substantial heterogeneity.
g. The 95% CI of the mean difference includes no effect.
h. All included studies lack blinding from patients and probably from caregivers, given it's impossible to blind people from a dietary intervention; seven out of sixteen included studies did not provide enough information to judge allocation concealment; four out of sixteen included studies did not follow intention-to-treat principle in data analysis; six out of sixteen included studies were at risk of selective reporting.
i. I^2=92% and tau-square=4·73 indicate a considerable heterogeneity.

© 2023 Wang T et al. *JAMA Network Open.*

eTable 9. GRADE assessment of the certainty of evidence in people at high risk of CVD

Certainty assessment							№ of patients		Effect		
№ of studies	Study design	Risk of bias	Inconsistency	Indirectness	Imprecision	Other considerations	Vegetarian dietary patterns	Other intervention diets or the usual diet	MD (95% CI)	Certainty	Importance
**Low-density lipoprotein cholesterol (follow-up: range 8 to 52 weeks)**											
9	RCTs	serious[a]	not serious	not serious	not serious	strong association	391	395	**-9·1 mg/dL** (-12·7, -5·5)	⊕⊕⊕⊕ High	Critical
**Hemoglobin A1c (follow-up: range 16 weeks to 6 months)**											
3	RCTs	not serious[b]	very serious[c]	not serious	very serious[d]	none	166	158	**-0·19 %** (-0·50, 0·11)	⊕◯◯◯ Very low	Critical
**Systolic blood pressure (follow-up: range 8 weeks to 6 months)**											
5	RCTs	not serious[e]	serious[f]	not serious	very serious[d]	none	122	127	**-2·5 mmHg** (-7·2, 2·3)	⊕◯◯◯ Very low	Critical
**Body Weight (follow-up: range 8 to 52 weeks)**											
9	RCTs	serious[a]	very serious[g]	not serious	not serious	none	392	394	**-3·6 kg** (-5·8, -1·4)	⊕◯◯◯ Very low	Important

CI: confidence interval; CVD: cardiovascular disease; MD: mean difference; RCT: randomized controlled trial

a. All included studies lack blinding from patients and probably from caregivers, given it is impossible to blind people from a dietary intervention; five out of nine included studies did not provide enough information to judge allocation concealment; three out of nine included studies did not follow intention-to-treat principle in data analysis; three out of nine included studies were at risk of selective reporting.
b. All included studies lack blinding from patients and probably from caregivers, given it's impossible to blind people from a dietary intervention; all included studies performed allocation concealment properly; all included studies follow intention-to-treat principle in data analysis; one out of three included studies was at risk of selective reporting.
c. I2=97% and tau-square=0·078 indicate a considerable heterogeneity.
d. The sample size is smaller than 400 and 95%CI of the mean difference include 0.
e. All included studies lack blinding from patients and probably from caregivers, given it's impossible to blind people from a dietary intervention; three out of five included studies did not provide enough information to judge allocation concealment; two out of five included studies did not follow intention-to-treat principle in data analysis; one out of five included studies were at risk of selective reporting.
f. I2=66% and tau-square=21·07 indicate substantial heterogeneity.
g. I2=95% and tau-square=8·67 indicate a considerable heterogeneity.

© 2023 Wang T et al. *JAMA Network Open.*

eTable 10. GRADE assessment of the certainty of evidence in people with T2DM

Certainty assessment							№ of patients		Effect		
№ of studies	Study design	Risk of bias	Inconsistency	Indirectness	Imprecision	Other considerations	Vegetarian dietary patterns	Other intervention diets or the usual diet	MD (95% CI)	Certainty	Importance
**Low-density lipoprotein cholesterol (follow-up: range 12 weeks to 24 weeks)**											
6	RCTs	not serious[a]	serious[b]	not serious	serious[c]	none	305	313	**-2·2 mg/dL** (-6.5, 2·1)	⨁⨁◯◯ Low	Important
**Hemoglobin A1c (follow-up: range 12 weeks to 24 weeks)**											
6	RCTs	not serious[d]	not serious	not serious	serious[e]	none	177	177	**-0·36 %** (-0·53, -0·18)	⨁⨁⨁◯ Moderate	Critical
**Systolic blood pressure (follow-up: range 12 weeks to 22 weeks)**											
6	RCTs	not serious[f]	not serious	not serious	serious[c]	none	269	280	**2·2 mmHg** (-0·6, 5)	⨁⨁⨁◯ Moderate	Important
**Body weight (follow-up: range 12 weeks to 22 weeks)**											
5	RCTs	serious[g]	serious[h]	not serious	not serious	none	235	240	**-2·8 kg** (-4·2, -1·4)	⨁⨁◯◯ Low	Important

CI: confidence interval; MD: mean difference; RCT: randomised controlled trial; T2DM: type 2 diabetes mellitus

a. All studies lack blinding from patients and probably from caregivers, given it is impossible to blind people from a dietary intervention; 2 out of 6 studies did not provide enough information to judge allocation concealment; 1 out of 6 studies did not follow intention-to-treat principle in data analysis; 1 out of 6 studies were at high risk of selective reporting and another presented some concerns of selective reporting.
b. I^2=63% and tau-square=17·80 indicate a substantial heterogeneity.
c. The 95% CI of the mean difference includes no effect, therefore rating down for imprecision.
d. All included studies lack blinding from patients and probably from caregivers, given it's impossible to blind people from a dietary intervention; two out of six included studies did not provide enough information to judge allocation concealment; all studies follow intention-to-treat principle in data analysis; two out of six included studies were at risk of selective reporting.
e. The sample size is lower than 400, therefore rating down for imprecision.
f. All included studies lack blinding from patients and probably from caregivers, given it's impossible to blind people from a dietary intervention; two out of six included studies did not provide enough information to judge allocation concealment; one out of six included studies did not follow intention-to-treat principle in data analysis; two out of six included studies were at risk of selective reporting.
g. All included studies lack blinding from patients and probably from caregivers, given it's impossible to blind people from a dietary intervention; one out of five included studies did not provide enough information to judge allocation concealment; one out of five included studies did not follow intention-to-treat principle in data analysis; two out of five included studies were at risk of selective reporting.
h. I^2=67% and tau-square=0·83 indicate a substantial heterogeneity.

© 2023 Wang T et al. *JAMA Network Open.*

eTable 11. GRADE assessment of the certainty of evidence in people with CVD

Certainty assessment							№ of patients		Effect	Certainty	Importance
№ of studies	Study design	MD (95% CI)	Inconsistency	Indirectness	Imprecision	Other considerations	Vegetarian dietary patterns	Other intervention diets or the usual diet	Absolute (95% CI)		
**Low-density lipoprotein cholesterol (follow-up: range 8 weeks to 2 years)**											
4	RCTs	not serious[a]	serious[b]	not serious	serious[c]	none	131	126	**-13·2 mg/dL** (-33·0, 6·6)	⊕⊕◯◯ Low	Important
**Systolic blood pressure (follow-up: range 1 years to 2 years)**											
3	RCTs	not serious[d]	not serious	not serious	very serious[e]	none	81	76	**-0·1 mmHg** (-6·1, 5·9)	⊕⊕◯◯ Low	Important

CI: confidence interval; CVD: cardiovascular disease; MD: mean difference; RCT: randomised controlled trial

a. All included studies lack blinding from patients and probably from caregivers, given it is impossible to blind people from a dietary intervention; two out of four included studies did not provide enough information to judge allocation concealment; all included studies follow intention-to-treat principle in data analysis; two out of four included studies were at risk of selective reporting.
b. I^2=80% and tau-sqaure=194·24 indicate a considerable heteorgeneity.
c. The sample size is lower than 400 and 95%CI of the mean difference includes no effect, therefore rating down for imprecision.
d. All included studies lack blinding from patients and probably from caregivers, given it's impossible to blind people from a dietary intervention; two out of three included studies did not provide enough information to judge allocation concealment; all included studies follow intention-to-treat principle in data analysis; one out of three included studies were at risk of selective reporting.
e. The sample size is lower than 200 and 95%CI include no effect, therefore rating down for imprecision.

© 2023 Wang T et al. *JAMA Network Open.*

eTable 12. Studies excluded at stage 2 with reasons

Author, year	Journal	Volume (issue):pages	Title	Exclusion reason
Acharya *et al.* 2009 [58]	Patient preference & adherence	3:151-60	Adherence to a behavioral weight loss treatment program enhances weight loss and improvements in biomarkers	No additional data to extract
Acharya *et al.* 2013 [59]	Journal of the American College of Nutrition	32(4):264-71	Weight loss is more important than the diet type in improving adiponectin levels among overweight/obese adults	No additional data to extract
Adebawo *et al.* 2006 [60]	Lipids in Health & Disease	5:14	Fruits and vegetables moderate lipid cardiovascular risk factor in hypertensive patients	Wrong study design
Agarwal *et al.* 2013 [61]	Diabetes	62:A195	A plant-based diet reduces depression and anxiety and improves work productivity: The GEICO multicenter trial	Not a manuscript
Agarwal *et al.* 2015 [62]	American Journal of Health Promotion	29(4):245-54	A multicenter randomized controlled trial of a nutrition intervention program in a multiethnic adult population in the corporate setting reduces depression and anxiety and improves quality of life: the GEICO study	Not relevant outcomes
Agren *et al.* 2001 [63]	British Journal of Nutrition	85(2):137-9	Divergent changes in serum sterols during a strict uncooked vegan diet in patients with rheumatoid arthritis	Wrong patient population
Ahc 2018 [64]	Clinical Cardiology Alert	37(6)	Vegetarian Diet vs. Mediterranean Diet to Reduce Cardiovascular Risk	Not a manuscript
Allen *et al.* 2015 [65]	Cardiology	131:91	The effects of plant-based, mediterranean, paleolithic, and dash diets on cardiovascular disease risk	Not a manuscript
Anderson 1983 [66]	Annals of Internal Medicine	98:842-6	Plant fiber and blood pressure	Wrong study design
Anderson 2018 [67]	Circulation	137(11):1114-1116	Dietary Patterns to Reduce Weight and Optimize Cardiovascular Health: Persuasive Evidence for Promoting Multiple, Healthful Approaches	Not a manuscript
Andrews 2017 [68]	Communicating Food for Health	2017:8	Vegetarian Diets & Heart Disease	Wrong study design
Anonymous 2002 [69]	Pharmaceutical Journal	269(7227):799	Vegetarian diet cuts cholesterol	Not a manuscript
Anonymous 2003 [70]	Pharmaceutical Journal	271(7260):140	Special cholesterol-lowering diet can achieve same effects as statin therapy	Not a manuscript
Arntzenius 1986 [71]	Drugs	31(SUPPL. 1):61-65	Diet, lipoproteins and the progression of coronary atherosclerosis. The Leiden Intervention Trial	Wrong study design
Barnard *et al.* 1992 [72]	Journal of Cardiopulmonary Rehabilitation	12(6):423-431	Adherence and acceptability of a low-fat, vegetarian diet among patients with cardiac disease	No additional data to extract
Barnard *et al.* 2000 [73]	American Journal of Cardiology	85(8):969-72	Effectiveness of a low-fat vegetarian diet in altering serum lipids in healthy premenopausal women	Wrong patient population
Barnard *et al.* 2004 [74]	Journal of Cardiopulmonary Rehabilitation	24(4):229-35	Acceptability of a low-fat vegan diet compares favorably to a step II diet in a randomized, controlled trial	Wrong patient population
Barnard *et al.* 2005 [75]	American Journal of Medicine	118(9):991-7	The effects of a low-fat, plant-based dietary intervention on body weight, metabolism, and insulin sensitivity	Wrong patient population

© 2023 Wang T et al. *JAMA Network Open.*

Author, year	Journal	Volume (issue):pages	Title	Exclusion reason
Barnard *et al.* 2009 [76]	Nutrition	25(1):58-65	D2 dopamine receptor Taq1A polymorphism, body weight, and dietary intake in type 2 diabetes	Not relevant outcomes
Barnard *et al.* 2020 [77]	Journal of the Academy of Nutrition & Dietetics	10:10	Blood Type Is Not Associated with Changes in Cardiometabolic Outcomes in Response to a Plant-Based Dietary Intervention	Wrong patient population
Beilin 1993 [78]	Annals of the New York Academy of Sciences	676:83-91	Vegetarian diets, alcohol consumption, and hypertension	Wrong study design
Bennett *et al.* 1990 [79]	American Journal of Clinical Nutrition	52(5):808-12	Diet and female sex hormone concentrations: an intervention study for the type of fat consumed	Wrong patient population
Bhardwaj 2017 [80]	Heliyon	3(12):e00472	A randomized controlled trial to evaluate the effects of high Protein Complete (lActo) VEgetaRian (PACER) diet in non-diabetic obese Asian Indians in North India	Wrong comparator
Brestrich 1996 [81]	Zeitschrift fur Kardiologie	85(6):418-427	Lactovegetarian diet: Influence on weight, lipids, fibrinogen and lipoprotein (a) of heart-patients in the course of their clinical rehabilitation. [German]	Other languages
Brom 2009 [82]	South African Family Practice	51(3):196-197	Integrative approach to management of heart disease is heart disease reversible?	Not a manuscript
Bunner 2014 [83]	Diabetes	63:A578	Nutrition intervention for diabetic neuropathy	Not a manuscript
Burke 2007 [84]	American Journal of Clinical Nutrition	86(3):588-96	Effects of a vegetarian diet and treatment preference on biochemical and dietary variables in overweight and obese adults: a randomized clinical trial	Wrong patient population
Campbell 2017 [85]	Journal of Geriatric Cardiology	14(5):321-326	A plant-based diet and stroke	Wrong study design
Campbell 1999 [86]	American Journal of Clinical Nutrition	70(6):1032-1039	Effects of an omnivorous diet compared with a lactoovovegetarian diet on resistance-training-induced changes in body composition and skeletal muscle in older men	Wrong study design
Campbell 2010 [87]	Journals of Gerontology Series A-Biological Sciences & Medical Sciences	65(10):1115-22	Protein intake, weight loss, and bone mineral density in postmenopausal women	Not relevant outcomes
Cesari 2019 [88]	Nutrition Metabolism & Cardiovascular Diseases	29(6):604-610	Mediterranean, but not lacto-ovo-vegetarian, diet positively influence circulating progenitor cells for cardiovascular prevention: The CARDIVEG study	Not relevant outcomes
Chamorro 2020 [89]	Lipids	55(6):639-648	Diet, Plasma, Erythrocytes, and Spermatozoa Fatty Acid Composition Changes in Young Vegan Men	Wrong study design
Crimarco 2020 [90]	American Journal of Clinical Nutrition	112(5):1188-1199	A randomized crossover trial on the effect of plant-based compared with animal-based meat on trimethylamine-N-oxide and cardiovascular disease risk factors in generally healthy adults: Study With Appetizing Plantfood-Meat Eating Alternative Trial (SWAP-MEAT)	Wrong intervention
Dewell 2008 [91]	Journal of the American Dietetic Association	108(2):347-56	A very-low-fat vegan diet increases intake of protective dietary factors and decreases intake of pathogenic dietary factors	Wrong intervention

© 2023 Wang T et al. *JAMA Network Open.*

Author, year	Journal	Volume (issue):pages	Title	Exclusion reason
Dinu 2020 [92]	International Journal of Food Sciences & Nutrition	71(3):362-369	Effects of a dietary intervention with Mediterranean and vegetarian diets on hormones that influence energy balance: CARDIVEG study	Not relevant outcomes
Dinu 2019 [93]	British Journal of Nutrition	121(7):756-762	Effects of a 3-month dietary intervention with a lacto-ovo-vegetarian diet on vitamin B12 levels in a group of omnivores: results from the CARDIVEG (Cardiovascular Prevention with Vegetarian Diet) study	Not relevant outcomes
Dinu 2019 [94]	Nutrition, Metabolism and Cardiovascular Diseases	29(8):882-883	Effects of a 3-months' dietary intervention with lacto-ovo-vegetarian diet on vitamin B12 levels: results of the CARDIVEG study	Wrong study design
Dinu 2019 [95]	Nutrition, Metabolism and Cardiovascular Diseases	29(8):881	Dietary intervention with vegetarian and mediterranean diets for cardiovascular prevention: Effects on hormones involved in the energy balance	Not relevant outcomes
Dogra 2020 [96]	Circulation	142(SUPPL 3)	Long-term Dietary and Weight Changes Following a Short-term Dietary Intervention Study: 4-year Follow-up of the Evade Cad Trial	Not a manuscript
Elkan 2008 [97]	Arthritis Research & Therapy	10(2):R34	Gluten-free vegan diet induces decreased LDL and oxidized LDL levels and raised atheroprotective natural antibodies against phosphorylcholine in patients with rheumatoid arthritis: a randomized study	Wrong patient population
Elkoustaf 2017 [98]	Journal of the American College of Cardiology	69 (11 Supplement 1):48	Diet and ischemic burden: A comparative analysis of a plant based regimen versus a mediterranean regimen in patients with coronary artery disease	Not a manuscript
Elkoustaf 2019 [99]	The Permanente journal	23:18.196	Lifestyle Interventions and Carotid Plaque Burden: A Comparative Analysis of Two Lifestyle Intervention Programs in Patients with Coronary Artery Disease	Wrong intervention
Fallah Noroozinejad 2015 [100]	Iranian Journal of Endocrinology and Metabolism	16(5)	Effect of legumes intake on the oxidative stress markers in type 2 diabetes patients: A randomized cross-over study. [Persian]	Other languages
Feifer 2003 [101]	Californian Journal of Health Promotion	1(3):7-13	Lifestyle change for weight loss in the inner-city	Wrong study design
Fenton 2019 [102]	Critical Reviews in Food Science & Nutrition	59(7):1044-1045	Plant-based diets do not prevent most chronic diseases	Wrong study design
Ferdowsian 2010 [103]	American Journal of Health Promotion	24(6):384-7	A multicomponent intervention reduces body weight and cardiovascular risk at a GEICO corporate site	Wrong study design
Flynn 2010 [104]	Journal of Women's Health	19(6):1155-1161	Comparing an olive oil-enriched diet to a standard lower-fat diet for weight loss in breast cancer survivors: a pilot study	Wrong intervention
Frassetto 2019 [105]	Journal of Evolution and Health	3	The VA Beach Diet Study: A comparison of the effects of Plant-based, Mediterranean, Paleolithic, and DASH Diets on cardiovascular disease risk	Not a manuscript
Gage 2011 [106]	Topics in Geriatric Rehabilitation	27(2):162-166	Growing old in a circle of friends: Sangha and the lifestyle heart trial	Wrong study design
Gardner 2007 [107]	JAMA	297(9):969-977	Comparison of the Atkins, Zone, Ornish, and LEARN Diets for Change in Weight and Related Risk Factors Among Overweight Premenopausal WomenThe A TO Z Weight Loss Study: A Randomized Trial	Wrong intervention

© 2023 Wang T et al. *JAMA Network Open.*

Author, year	Journal	Volume (issue):pages	Title	Exclusion reason
Gorder 1986 [108]	Journal of the American Dietetic Association	86(6):744-751	Dietary intake in the Multiple Risk Factor Intervention Trial (MRFIT): Nutrient and food group changes over 6 years	Wrong intervention
Gould 1992 [109]	American Journal of Cardiology	69(9):845-53	Improved stenosis geometry by quantitative coronary arteriography after vigorous risk factor modification	Not relevant outcomes
Gould 1995 [110]	JAMA	274(11):894-901	Changes in myocardial perfusion abnormalities by positron emission tomography after long-term, intense risk factor modification	Not relevant outcomes
Hakala 1989 [111]	European Journal of Clinical Nutrition	43(6):421-30	Weight reduction on lactovegetarian and mixed diets. Changes in weight, nutrient intake, skinfold thicknesses and blood pressure	Wrong patient population
Harsha 1999 [112]	Journal of the American Dietetic Association	99(8 SUPPL.):S35-S39	Dietary Approaches to Stop Hypertension: A summary of study results	Wrong intervention
Harvey 2020 [113]	Heart, Lung & Circulation	29:S375-S375	The Effect of a Plant-Based Diet on Cardiovascular Risk Factors in Patients With Ischaemic Heart Disease	Wrong study design
Haub 2005 [114]	Metabolism: Clinical & Experimental	54(6):769-74	Beef and soy-based food supplements differentially affect serum lipoprotein-lipid profiles because of changes in carbohydrate intake and novel nutrient intake ratios in older men who resistive-train	Wrong patient population
Hosseinpour-Niazi 2015 [115]	European Journal of Clinical Nutrition	69(5):592-597	Substitution of red meat with legumes in the therapeutic lifestyle change diet based on dietary advice improves cardiometabolic risk factors in overweight type 2 diabetes patients: a cross-over randomized clinical trial	Wrong intervention
Hunt 1998 [116]	American Journal of Clinical Nutrition	67(3):421-30	Zinc absorption, mineral balance, and blood lipids in women consuming controlled lactoovovegetarian and omnivorous diets for 8 wk	Wrong patient population
Jenkins 2010 [117]	CMAJ : Canadian Medical Association journal	182(18):1961-1967	Adding monounsaturated fatty acids to a dietary portfolio of cholesterol-lowering foods in hypercholesterolemia	Wrong intervention
Jibani 1991 [118]	Diabetic Medicine	8(10):949-53	Predominantly vegetarian diet in patients with incipient and early clinical diabetic nephropathy: effects on albumin excretion rate and nutritional status	Wrong study design
Kahleova 2018 [119]	Nutrition & Diabetes	8(1):58	A plant-based diet in overweight individuals in a 16-week randomized clinical trial: metabolic benefits of plant protein	No additional data to extract
Kahleova 2014 [120]	Cor et Vasa	56(2):e140-e144	Vegetarian vs. conventional diabetic diet - A 1-year follow-up	Other languages
Kahleova 2017 [121]	Journal of the American College of Nutrition	36(5):364-369	The Effect of a Vegetarian vs Conventional Hypocaloric Diabetic Diet on Thigh Adipose Tissue Distribution in Subjects with Type 2 Diabetes: A Randomized Study	Not relevant outcomes
Kahleova 2017 [122]	Diabetes	66 (Supplement 1):A202	The effect of a vegetarian vs. Conventional hypocaloric diabetic diet on thigh adipose tissue distribution in subjects with type 2 diabetes	Not relevant outcomes
Kahleova 2011 [123]	Diabetes	60:A217	Vegetarian diet increases resting energy expenditure more than conventional diet in subjects with type 2 diabetes after aerobic exercise	Not a manuscript
Kahleova 2011 [124]	Diabetologia	54:S359-S360	The effect of vegetarian diet on fatty acid composition of serum phopholipids and the association with insulin sensitivity and visceral fat in subjects with type 2 diabetes	Not a manuscript

© 2023 Wang T et al. *JAMA Network Open.*

Author, year	Journal	Volume (issue):pages	Title	Exclusion reason
Kahleova 2010 [125]	Diabetologia	53:S380	Vegetarian diet improves plasma concentrations of adipokines and oxidative stress markers more than conventional diabetic diet in subjects with type 2 diabetes	Not a manuscript
Kahleova 2020 [126]	Diabetologia	63(SUPPL 1):S16-S17	A dietary intervention to alter insulin sensitivity, intramyocellular and hepatocellular lipids, postprandial metabolism, and body weight: A 16-week randomised trial	Not a manuscript
Kahleova 2020 [127]	Nutrients	12(10):24	Effects of a Low-Fat Vegan Diet on Gut Microbiota in Overweight Individuals and Relationships with Body Weight, Body Composition, and Insulin Sensitivity. A Randomized Clinical Trial	Wrong patient population
Kahleova 2021 [128]	Clinical Nutrition ESPEN	41:126-128	Effect of a diet intervention on cardiometabolic outcomes: Does race matter? A randomized clinical trial	Wrong patient population
Kahleova 2016 [129]	Nutrition Metabolism & Cardiovascular Diseases	26(5):430-8	The effect of a vegetarian versus conventional hypocaloric diet on serum concentrations of persistent organic pollutants in patients with type 2 diabetes	No additional data to extract
Kahleova 2018 [130]	Nutrients	10(2):09	A Plant-Based Dietary Intervention Improves Beta-Cell Function and Insulin Resistance in Overweight Adults: A 16-Week Randomized Clinical Trial	No additional data to extract
Kahleova 2018 [131]	Nutrients	10(9):1302	A Plant-Based High-Carbohydrate, Low-Fat Diet in Overweight Individuals in a 16-Week Randomized Clinical Trial: The Role of Carbohydrates	Not relevant outcomes
Kahleova 2019 [132]	Nutrients	11(3):615-615	Fat Quantity and Quality, as Part of a Low-Fat, Vegan Diet, Are Associated with Changes in Body Composition, Insulin Resistance, and Insulin Secretion. A 16-Week Randomized Controlled Trial	No additional data to extract
Kahleova 2021 [133]	Clinical Nutrition ESPEN	NA	A plant-based diet in overweight adults in a 16-week randomized clinical trial: The role of dietary acid load	No additional data to extract
Kalinina 1958 [134]	Terapevticheskii Arkhiv	30(7):72-6	[Changes of arterial pressure, pulse, circulation rate and electrocardiographic indices in hypertension following meat and vegetable diets]	Other languages
Katcher 2010 [135]	Annals of Nutrition & Metabolism	56(4):245-52	A worksite vegan nutrition program is well-accepted and improves health-related quality of life and work productivity	Wrong study design
Kestin 1989 [136]	American Journal of Clinical Nutrition	50(2):280-7	Cardiovascular disease risk factors in free-living men: comparison of two prudent diets, one based on lactoovovegetarianism and the other allowing lean meat	Study duration < 8 weeks
Kjeldsen-Kragh 1991 [137]	The Lancet	338(8772):899-902	Controlled trial of fasting and one-year vegetarian diet in rheumatoid arthritis	Not relevant outcomes
Kochan 2019 [138]	Clinical Nutrition	38 (Supplement 1):S233	N-3 Pufa-Enriched Semi-Vegetarian Diet Lowers Ldl-Cholesterol and Uric Acid Levels in Patients with Familial Hypercholesterolemia	Wrong study design
Koertge 2003 [139]	American Journal of Cardiology	91(11):1316-1322	Improvement in medical risk factors and quality of life in women and men with coronary artery disease in the Multicenter Lifestyle Demonstration Project	Wrong study design
Krishnamoorthy 1999 [140]	Indian Heart Journal	51(3):268-74	Diet and coronary artery disease	Wrong study design

© 2023 Wang T et al. *JAMA Network Open.*

Author, year	Journal	Volume (issue):pages	Title	Exclusion reason
Lasserre 2017 [141]	Annals of Nutrition & Metabolism	71(895)	Effectiveness of a dietotherapeutic intervention on the lipids profile and the nutritional status in breast cancer women	Not a manuscript
Levin 2010 [142]	Public Health Nutrition	13(10):1629-35	A worksite programme significantly alters nutrient intakes	Wrong study design
Mangels 2019 [143]	Vegetarian Journal	38(2):13-13	People with Type 2 Diabetes Benefit from Vegan and "Plant-Based" Diets	Wrong study design
Marniemi 1990 [144]	International Journal of Obesity	14(2):113-25	Long-term effects on lipid metabolism of weight reduction on lactovegetarian and mixed diet	Wrong patient population
Masarei 1984 [145]	American Journal of Clinical Nutrition	40(3):468-478	Effects of a lacto-ovo vegetarian diet on serum concentrations of cholesterol, triglyceride, HDL-C, HDL2-C, HDL3-C, apoprotein-B, and Lp(a)	Study duration < 8 weeks
Medkova 2000 [146]	Klinicheskaia Meditsina	78(1):21-24	Blood lipids and intensity of free radical oxidant processes in elderly patients with ischemic heart disease on antiatherogenic vegetarian diet. [Russian]	Other languages
Medkova 2006 [147]	Voprosy Pitaniia	75(5):49-52	Clinicohemadynamic and biochemical the effect of patients with coronary heart disease use combinated lactoovovegetarian diets and simvastatin. [Russian]	Other languages
Medkova 2002 [148]	Voprosy Pitaniia	71(4):17-19	Estimation of action of lactoovovegetarian and vegan diets on blood level of atherogenic lipoproteins in healthy people. [Russian]	Other languages
Medkova 1997 [149]	Klinicheskaia Meditsina	75(1):28-31	[Balanced vegetarian diet in combined rehabilitation of patients suffering from ischemic heart disease]	Other languages
Mishra 2012 [150]	Diabetes	61:A192	A plant-based diet reduces body weight and cardiovascular risk: The geico multicenter trial	Not a manuscript
Misquitta 2020 [151]	Journal of Alternative and Complementary Medicine	26(11):A9	Preliminary evaluation of the health achieved through lifestyle transformation (HALT) program, a kaiser permanente lifestyle intervention	Not a manuscript
Monica Dinu 2017 [152]	European Journal of Preventive Cardiology	24 (1 Supplement 1):S136	Comparison between Mediterranean and Vegetarian diets for cardiovascular prevention: The CARDIVEG study	Not a manuscript
Moore 2015 [153]	Eating Behaviors	19:33-8	Dietary adherence and acceptability of five different diets, including vegan and vegetarian diets, for weight loss: The New DIETs study	Wrong patient population
Navas-Carretero 2009 [154]	British Journal of Nutrition	102(4):546-553	An oily fish diet increases insulin sensitivity compared to a red meat diet in young iron-deficient women	Wrong intervention
Nichols 2014 [155]	Clinical Advisor	17(8):24-38	Vegetarianism in the fight against CVD	Wrong study design
Nicholson 1999 [156]	American Journal of Clinical Nutrition	70(35):624S-625S	Effect of a low-fat, unrefined, vegan diet on type 2 diabetes	Not a manuscript
Nomura 1989 [157]	Nippon Rinsho - Japanese Journal of Clinical Medicine	47(9):2074-2078	Non-pharmacological treatment of hypertension. [Japanese]	Other languages
Pagliai 2016 [158]	European Heart Journal	37(340)	Randomized controlled dietary intervention trial comparing mediterranean and vegetarian diets for cardiovascular prevention: preliminary results	Not a manuscript

© 2023 Wang T et al. *JAMA Network Open.*

Author, year	Journal	Volume (issue):pages	Title	Exclusion reason
Pagliai 2017 [159]	Nutrition, Metabolism and Cardiovascular Diseases	27 (1):e30-e31	Comparison between mediterranean and vegetarian diets for cardiovascular prevention: The cardiveg study	Not a manuscript
Pagliai 2019 [160]	Nutrition, Metabolism and Cardiovascular Diseases	29(8):879	Impact of mediterranean vs vegetarian diets on gut microbiota and short chain fatty acids: the CARDIVEG study	Not a manuscript
Pagliai 2020 [161]	European Journal of Nutrition	59(5):2011-2024	Influence of a 3-month low-calorie Mediterranean diet compared to the vegetarian diet on human gut microbiota and SCFA: the CARDIVEG Study	Not relevant outcomes
Phillips 1999 [162]	Journal of the American Dietetic Association	99(8 SUPPL.):S60-S68	Validation of diet composition for the dietary approaches to stop hypertension trial	Wrong intervention
Prescott 1987 [163]	Clinical & Experimental Pharmacology & Physiology	14(3):159-62	Controlled study of the effects of dietary protein on blood pressure in normotensive humans	Wrong patient population
Prescott 1988 [164]	Clinical Science	74(6):665-72	A randomized controlled trial of the effect on blood pressure of dietary non-meat protein versus meat protein in normotensive omnivores	Wrong patient population
Rajaram 2000 [165]	Nutrition	16(7-8):531-3	Health benefits of a vegetarian diet	Wrong study design
Rodriguez-Rodriguez 2007 [166]	Nutrition Research	27(6):313-320	Restricted-energy diets rich in vegetables or cereals improve cardiovascular risk factors in overweight/obese women	Study duration < 8 weeks
Rouse 1984 [167]	Journal of Hypertension	2(3):231-40	Vegetarian diet and blood pressure	Wrong study design
Rouse 1986 [168]	Journal of Hypertension	4(2):241-50	Nutrient intake, blood pressure, serum and urinary prostaglandins and serum thromboxane B2 in a controlled trial with a lacto-ovo-vegetarian diet	Study duration < 8 weeks
Rugulies 1996 [169]	Zeitschrift fur Gesundheitswissenschaften	4(3):234-247	Comprehensive lifestyle changes among patients with CHD and the integration of inpatient and outpatient cardiac rehabilitation: First results of a secondary prevention intervention trial. [German]	Other languages
Sanchez 2019 [170]	Cogent Medicine	6(1)	Multiple lifestyle interventions reverses hypertension	Wrong study design
Sanders 1977 [171]	Proceedings of the Nutrition Society	36(1):43A	Serum cholesterol and triglycerides concentrations in vegans	Wrong study design
SarveshKumar 2016 [172]	International Journal of Pharmaceutical Sciences Review and Research	39(2):81-84	Effect of aerobics exercise and dietary habits on blood pressure in hypertensives	Wrong study design
Schmidt 1997 [173]	Acta Physiologica Scandinavica	161(640):158-162	Changes in cardiovascular risk factors and hormones during a comprehensive residential three month kriya yoga training and vegetarian nutrition	Wrong study design
Sethna 1960 [174]	Indian Journal of Medical Research	225-230	Cholesterol and phospholipids in aged groups	Wrong study design
Singh 1992 [175]	American Journal of Cardiology	70(9):869-874	Effect of fat-modified and fruit- and vegetable-enriched diets on blood lipids in the Indian Diet Heart Study	Wrong intervention

© 2023 Wang T et al. *JAMA Network Open.*

Author, year	Journal	Volume (issue):pages	Title	Exclusion reason
Sinha 2012 [176]	Indian Journal of Physiology and Pharmacology	1:122	Study of cardiovascular autonomic function in relation to the duration of menopause and vegetarian diet	No full text
Soare 2014 [177]	Nutrition & Metabolism	11(1):39	The effect of the macrobiotic Ma-Pi 2 diet vs. the recommended diet in the management of type 2 diabetes: the randomized controlled MADIAB trial	Study duration < 8 weeks
Spence 2020 [178]	Neurology	94(11):463-464	Lower risk of stroke with a vegetarian diet	Wrong study design
Talreja 2021 [179]	Catheterization and Cardiovascular Interventions	97(SUPPL 1):S109	An investigation of plant-based, mediterranean, paleolithic, and dash diets study (the Va Beach Diet Study)	Not a manuscript
Talreja 2021 [180]	Journal of the American College of Cardiology	77(18 Supplement 1):1478	THE VA-BEACH DIET STUDY: A COMPARISON OF PLANT-BASED, MEDITERRANEAN, PALEOLITHIC AND DASH DIETS ON LIPID AND BIOMETRIC PARAMETERS	Not a manuscript
Talreja 2020 [181]	Circulation	142(SUPPL 3)	Prospective Comparison of Lipid Responses in Plant-based, Mediterranean, Paleolithic an Dash Diets	Wrong study design
Thedford 2011 [182]	Journal of the American Dietetic Association	111(6):816-8	A vegetarian diet for weight management	Wrong study design
Thomson 2005 [183]	European Journal of Nutrition	44(1):18-25	Longitudinal changes in body weight and body composition among women previously treated for breast cancer consuming a high-vegetable, fruit and fiber, low-fat diet	Wrong intervention
Tjiong 1952 [184]	Nederlands Tijdschrift voor Geneeskunde	96(8):472-474	Influence of diet and of some other factors on the blood cholesterol. [Dutch]	Other languages
Toobert 1998 [185]	Patient Education & Counseling	35(3):177-88	Behavioral and psychosocial effects of intensive lifestyle management for women with coronary heart disease	Not relevant outcomes
Turner-McGrievy 2007 [186]	Obesity	15(9):2276-81	A two-year randomized weight loss trial comparing a vegan diet to a more moderate low-fat diet	Wrong patient population
Turner-McGrievy 2004 [187]	Nutrition	20(9):738-46	Effects of a low-fat vegan diet and a Step II diet on macro- and micronutrient intakes in overweight postmenopausal women	Wrong patient population
Turner-McGrievy 2014 [188]	Nutrition Research	34(6):552-8	Low glycemic index vegan or low-calorie weight loss diets for women with polycystic ovary syndrome: a randomized controlled feasibility study	Not relevant outcomes
Turner-McGrievy 2015 [189]	Nutrition	31(2):350-8	Comparative effectiveness of plant-based diets for weight loss: a randomized controlled trial of five different diets	Wrong patient population
Turner-McGrievy 2016 [190]	Journal of Hunger and Environmental Nutrition	11(3):382-395	Differences in Environmental Impact and Food Expenditures of Four Different Plant-based Diets and an Omnivorous Diet: Results of a Randomized, Controlled Intervention	Wrong patient population
Turner-McGrievy 2015 [191]	Nutrition Research	35(2):97-106	Randomization to plant-based dietary approaches leads to larger short-term improvements in Dietary Inflammatory Index scores and macronutrient intake compared with diets that contain meat	Wrong patient population

© 2023 Wang T et al. *JAMA Network Open.*

Author, year	Journal	Volume (issue):pages	Title	Exclusion reason
Veleba 2016 [192]	Nutrients	8(11):26	"A Vegetarian vs. Conventional Hypocaloric Diet: The Effect on Physical Fitness in Response to Aerobic Exercise in Patients with Type 2 Diabetes." A Parallel Randomized Study	No additional data to extract
Whitten 1995 [193]	Topics in Clinical Nutrition	10(2):27-33	Vegetarian diets and ischemic heart disease	Wrong study design
Wirths 1988 [194]	Zeitschrift fur Ernahrungswissenschaft	27(2):84-100	[Effect of an egg-milk-vegetarian diet on nutritional and blood status. II. Findings of a study on circulation, blood status; discussion]	Other languages
Yarnell 1992 [195]	Annals of Internal Medicine	117(SUPPL. 3):76	Cardioprotective diet after recent myocardial infarction	Not a manuscript
Zeis 2008 [196]	Schweizerische Zeitschrift fur GanzheitsMedizin	20(3):144-148	Health effect of a fruit and vegetable rich diet. Part 1: Cardiovascular diseases. [German]	Other languages
Zemel 1997 [197]	Nutrition Reviews	55(8):303-305	Dietary pattern and hypertension: The DASH study	Wrong intervention
2006 [198]	Environmental Nutrition	29(10):1-1	Vegan diet helps treat type 2 diabetes	Not a manuscript
2019 [199]	Nephrology News & Issues	33(6):34-34	Plant-based diets can lower CV mortality risk	Wrong study design
2019 [200]	Clinical Nutrition ESPEN	30:42-51	Impact of a 12-month Inflammation Management Intervention on the Dietary Inflammatory Index, inflammation, and lipids	Wrong study design
2018 [201]	Gazzetta Medica Italiana Archivio per le Scienze Mediche	177(9):468-474	Effects of the lifestyle modification program to reduce serum lipoprotein(a) and other cardiovascular risk factors in Korean college women	Study duration < 8 weeks

© 2023 Wang T et al. *JAMA Network Open.*

eTable 13. Reported Conflicts of Interest (COI) and funding sources of included studies

Author, year	COI statement	Funding (e.g. industry/non-industry)	Funding details	COI exists
Aldana *et al.*, 2006 [24]	NI.	Non-industry.	Supported by the Excellence in Academic Medicine Act of the State of Illinois.	NI.
Barnard *et al.*, 2006 [25-28]	NDB is president of the Physicians Committee for Responsible Medicine and the Cancer Project, organizations that promote the use of low-fat, plant-based diets, and writes books and gives lectures ahout therapeutic diets, including vegan diets. He is the author of Dr. Neal Barnard's Program for Reversing Diabetes and receives royalties from its sales. None of the other authors had any personal or financial conflict of interest.	Non-industry.	The study was supported by grant R01 DK059362-01A2 from the National Institute of Diabetes and Digestive and Kidney Diseases and by the Diabetes Action Research and Education Foundation.	Suspicious.
Barnard *et al.*, 2018 [29]	NDB writes books and articles and gives lectures related to nutrition and health, and has received royalties and honoraria from these sources. NDB, SML, and RF are affiliated with the Physicians Committee for Responsible Medicine, which promotes the use of low-fat, plant-based diets and discourages the use of animal-derived, fatty, and sugary foods. LG practices medical nutrition therapy in her private practice, Nutrition Coaching, LLC, and at several worksite wellness centers.	Non-industry.	The study was supported by the Physicians Committee for Responsible Medicine.	No.
Barnard *et al.*, 2021 [30]	All authors except for AT and RH work for the Physicians Committee for Responsible Medicine in Washington, DC, a nonprofit organization providing educational, research, and medical services related to nutrition. Dr. Barnard is an Adjunct Professor of Medicine at the George Washington University School of Medicine. He serves without compensation as President of the Physicians Committee for Responsible Medicine and the Barnard Medical Center in Washington, DC. He writes books and articles and gives lectures related to nutrition and health and has received royalties and honoraria from these sources.	Non-industry.	Funded by the Physicians Committee for Responsible Medicine.	No.
Bunner *et al.*, 2015 [31]	The authors declare no conflict of interest.	Industry	We gratefully acknowledge Impeto Medical for the Sudoscan device. No other funding support was reported.	Unclear.
Burke *et al.*, 2006 [32-34]	NR.	Non-industry.	Funded by the National Institute of Diabetes and Digestive and Kidney Diseases (Grant RO1-DK58631); the Data Management Core of Center for Research in Chronic Disorders, National Institute of Nursing Research (Grant P30-NR03924); the Obesity and Nutrition Research Center of the National Institute of Diabetes and Digestive and Kidney Diseases (Grant DK-046204); the National Center for Research Resources/General Clinical Research Center (Grant 5MO1-RR00056) at the University of Pittsburgh.	NI.

© 2023 Wang T et al. *JAMA Network Open.*

Author, year	COI statement	Funding (e.g. industry/non-industry)	Funding details	COI exists
Garousi *et al.*, 2021 [35]	The authors declare no conflict of interest.	NA	Received no specific grant from any funding agency in the public, commercial, or not-for-profit sectors.	No.
Kahleova *et al.*, 2010 [36]	Nothing to declare.	Non-industry	Supported by grant IGA MZCR NS ⁄ 10534-3 from Ministry of Health, Prague, Czech Republic.	No.
Kahleova *et al.*, 2020 [37]	Dr Kahleova reported being director of clinical research at the Physicians Committee, a nonprofit organization that provides nutrition education and research. Dr Rembert reported compensation from the Physicians Committee for Responsible Medicine outside the submitted work. Dr Holubkov reported receiving personal fees from the Physicians Committee for Responsible Medicine during the conduct of the study. Dr Barnard reported to serving as president of the Physicians Committee for Responsible Medicine and Barnard Medical Center; receiving royalties from Hachette Book Group, Penguin Random House, Rodale, and Da Capo publishers; and receiving honoraria from Yale, Rush, GeorgeWashington, Loma Linda, Rockford Universities, Montefiore Medical Center, the Mayo Clinic, Northwell Health, Christiana Care, Oticon, and the National Organization of Professional Athletes. No other disclosures were reported.	Non-industry.	Funded by the Physicians Committee for Responsible Medicine and grants P30 DK-045735 and R01 DK-113984 from the Yale Diabetes Center (Drs Shulman and Petersen). The Yale Diabetes Center had no role in the design and conduct of the study; collection, management, analysis, and interpretation of the data; preparation, review, or approval of the manuscript; and decision to submit the manuscript for publication.	No.
Lee *et al.*, 2016 [38]	The authors have declared that no competing interests exist.	Industry and non-industry	This research was supported by the Korea Health Industry Development Institute, funded by the Ministry of Health & Welfare (A111716-1202-0000100), as well as the Korean Health Technology R&D Project, funded by the Ministry of Health and Welfare, Republic of Korea (HI13C0715 and HI11C1300).	No.
Liao *et al.*, 2007 [39]	NR	Industry	The American Soy Association provided funding for this project.	Probably yes.
Mahon *et al.*, 2007 [40]	NI	Industry and non-industry.	Cattlemen's Beef Board and the National Cattlemen's Beef Association (Centennial, CO), Agriculture Research Program & Lynn Fellowships at Purdue University, and NIH R29 AG13409	Suspicious
Mishra *et al.*, 2013 [41,42]	Dr Neal Barnard gives lectures and writes books on the subject of plant-based diets and receives occasional honoraria and royalties therefrom. The remaining authors declare no conflict of interest.	Non-industry.	This research is supported by Physicians Committee for Responsible Medicine.	No.
Nicholson *et al.*, 1999 [43]	Reported by corresponding author: Two of the investigators were employees of the Physicians Committee for Responsible Medicine, a nonprofit organization providing educational and research services related to nutrition. In addition, Dr. Barnard writes books and gives lectures on health and nutrition and receives royalties and honoraria from these sources.	Non-industry	Supported by a grant from the Diabetes Action Research and Education Foundation, with additional funding from the Physicians Committee for Responsible Medicine.	PN.

© 2023 Wang T et al. *JAMA Network Open.*

Author, year	COI statement	Funding (e.g. industry/non-industry)	Funding details	COI exists
Ornish *et al.*, 1990 [44,45]	NR	Industry and non-industry.	This study was supported by grants from the National Heart, Lung, and Blood Institute of the National Institutes of Health (ROl HL42554), the Department of Health Services of the State of California (no 1256SC-01), Gerald D. Hines Interests, Houston Endowment Inc, the Henry J. Kaiser Family Foundation. the John E. Fetzer Institute, Continental Airlines, the Enron Foundation, the Nathan Cummings Foundation, the Pritzker Foundation, the First Boston Corporation, Quaker Oats Co., Texas Commerce Bank, Corrine and David Gould, Pacific Presbyterian Medical Center Foundation, General Growth Companies, Arthur Andersen and Co., and others.	NI
Shah *et al.*, 2018 [46,47]	None.	Probably industry_Private charitable trust	The Purjes Foundation (Salt Lake City, UT) was the primary sponsor of the trial and did not contribute to the study design or data analysis.	PN.
Sofi *et al.*, 2018 [48,49]	None.	None.	NA	No.
Tang *et al.*, 2013 [50]	The authors have no conflict of interest.	Industry and non-industry.	National Pork Board; American Egg Board-Egg Nutrition Center; Purdue Ingestive Behavior Research Center.	PN.
Toobert *et al.*, 2000 [51]	NI.	Non-industry	Study was supported in part by grant R29 HL50181 from the National Heart, Lung, and Blood Institute.	NI.
Wright *et al.*, 2017 [52]	NW is employed by the Royal NZ college of GPs, which is a position funded by Health Workforce New Zealand. MS and NW report being directors/shareholders in Plant Based Lifestyles Ltd, which was initiated after the completion of the BROAD study. BD, NW, MS and PMH report being trustees of the Plant Based New Zealand Health Charitable Trust. PMH reports he is a trustee on the Tairāwhiti Traditional and Complementary Therapies Research Charitable Trust. LW reports being director/shareholder in Two Zesty Bananas Ltd, which was initiated after the intervention.	Industry.	Tairāwhiti Traditional and Complementary Therapies Research Trust, the Tairāwhiti Community Services Trust and the J N Williams Memorial Trust. One researcher and author (PMH) is a trustee on TTCTRT, otherwise the funders had no role in study design and conduct; data collection and analysis; decision to publish; or preparation, review and approval of the manuscript.	No.

NA, not applicable; NI, no information; NR, not reported; PN, probably no.

© 2023 Wang T et al. *JAMA Network Open.*

eTable 14. Primary and secondary outcomes of included studies

Author, year	Primary outcomes	Secondary outcomes
Aldana *et al.*, 2006 [24]	Carotid Ultrasound Measures: Intimamedia thickness measures	Weight, BMI, blood lipids, blood glucose, CRP, ferritin, fibrinogen, medications, diet composition, program adherence, quality of life, and angina
Barnard *et al.*, 2006 [25-28]	HbA1c	Body weight, waist and hip circumference, plasma glucose, plasma cholesterol and triglyceride concentrations, HDL, LDL, urinary albumin, blood pressure, physical activity, dietary intake, dietary acceptability
Barnard *et al.*, 2018 [29]	Body weight, HbA1c, plasma glucose, plasma lipids, urinary albumin, and blood pressure, indices of renal function (serum creatinine, microalbumin)	Dietary intake, physical activity, medication use
Barnard *et al.*, 2021 [30]	Body weight, plasma lipids, insulin sensitivity, metabolism, blood pressure, and body composition (dual X-ray absorptiometry)	Oral glucose insulin sensitivity, and predicted insulin sensitivity indices, dietary intake
Bunner *et al.*, 2015 [31]	Painful symptoms of diabetic neuropathy: pain and sensory symptoms as measured by visual analog 'worst pain' scale, global impression scale, Short Form McGill Pain Questionnaire, Michigan Neuropathy Screening Instrument questionnaire, Neuropathy Total Symptom Score, a weekly pain diary, and Norfolk Quality of Life Questionnaire; disease activity; HbA1c; mood and depression were measured by the Beck Depression Inventory and the Center for Epidemiological Studies Depression revised scale	Body weight, lipid concentrations (TC, LDL-cholesterol, HDL-cholesterol and TG), blood glucose, blood pressure, dietary changes
Burke *et al.*, 2006 [32-34]	Body weight	Lipid profile (TC, HDL-C, LDL-C, and TG), serum glucose, and insulin measurement. Physical measurements included height, weight, waist circumference, and blood pressure. BMI.
Garousi *et al.*, 2021 [35]	A significant decrease in ALT and AST blood levels	Changes in anthropometric values, insulin metabolic markers, lipid profiles, blood pressure, and NAFLD grade
Kahleova *et al.*, 2010 [36]	Insulin sensitivity measured by hyperinsulinaemic isoglycaemic clamp; volume of visceral and subcutaneous fat measured by magnetic resonance imaging; and oxidative stress measured by thiobarbituric acid reactive substances	Weight, waist circumference, blood pressure and heart rate, plasma glucose, HbA1c, plasma immunoreactive insulin and C-peptide concentrations, plasma lipid, plasma concentrations of total adiponectin and resistin, dietary intake, physical activity, quality of life, magnetic resonance images with the calculation of subcutaneous and visceral abdominal fat volume
Kahleova *et al.*, 2020 [37]	Body weight, insulin resistance, postprandial metabolism, and the concentrations of intramyocellular and hepatocellular lipids	Body composition and visceral fat volume, dietary intake, physical activity, medication use, plasma glucose, immunoreactive insulin, and C-peptide concentrations, HbA1c, lipid concentration
Lee *et al.*, 2016 [38]	Change in HbA1c	Dietary intake, body weight, height, waist circumference, blood pressure, fasting blood glucose, blood lipids
Liao *et al.*, 2007 [39]	Weight, waist circumference, body composition, and blood lipid profiles	Blood pressure, biochemical measurements (serum glucose, triacylglycerols, TC, HDL-C, LDL-C, glutamate oxaloacetate transaminase, and glutamate pyruvate transaminase)
Mahon *et al.*, 2007 [40]	Body mass and body composition	Clinical markers of metabolic and cardiovascular diseases: Serum glucose, total cholesterol, HDL cholesterol and triacylglycerol, CRP, plasma insulin, leptin and adiponectin
Mishra *et al.*, 2013 [41,42]	Anthropometric (body weight) and biochemical measures (blood lipids, HbA1c)	Dietary intake, blood pressure

© 2023 Wang T et al. *JAMA Network Open.*

Author, year	Primary outcomes	Secondary outcomes
Nicholson *et al.*, 1999 [43]	Glycemic (fasting serum glucose, HbA1c) and blood lipid control	Body weight, blood pressure, urinary microalbumin, dietary intake, duration of exercise per week
Ornish *et al.*, 1990 [44,45]	Coronary atherosclerosis: coronary arteriography, selective coronary angiography	Body weight, blood lipid, blood pressure, dietary intake, exercise
Shah *et al.*, 2018 [46,47]	Inflammatory (hs-CRP concentration) and glucometabolic profiles	Anthropometric data, other markers of inflammation, lipid parameters, glycemic markers, endothelial function, dietary intake, quality of life data, and assessment of physical activity. Clinical outcomes: Major adverse cardiovascular events (MACE), Individual components of MACE.
Sofi *et al.*, 2018 [48,49]	Total body weight, BMI, and fat mass.	The circulating cardiovascular risk parameters: lipid profile, glycemic profile, oxidative stress profile, and inflammatory profile
Tang *et al.*, 2013 [50]	Changes in weight, body composition, indices of metabolic syndrome (blood pressure, blood lipid), resting energy expenditure, appetite, selected appetite hormones (insulin, leptin, ghrelin), whole body energy expenditure, and body composition (fat mass and fat-free mass) changes.	Serum insulin concentration, insulin resistance (HOMA-IR), kidney disease risk factor (GFR), total protein intake, dietary intake, the effects of meal frequency on appetite by asking the men to frequently rate their appetite on days that they purposefully eat 3 vs. 6 times/day.
Toobert *et al.*, 2000 [51]	Cardiovascular risk factors: blood lipid, blood pressure	Program adherence (the dietary, stress management, and physical activity changes); changes in body mass, hypolipidemic and anti hypertensive medications; angina symptoms and quality of life
Wright *et al.*, 2017 [52]	BMI and cholesterol	Changes in medication usage, quality of life, cardiovascular risk factors, cardiovascular events, or progression to surgery, and transfer to a higher level of care.

ALT, aminotransferase; AST, aspartate aminotransferase; BMI, body mass index; CHD, coronary heart disease; CRP, C-reactive protein; CVD, cardiovascular disease; HbA1c, hemoglobin A1c; HDL, high-density lipoprotein cholesterol; LDL, low-density lipoprotein cholesterol; LF, low-fat; MI, myocardial infarction; NAFLD, non-alcoholic fatty liver disease; NR, not reported; SBP, systolic blood pressure; T2DM, type 2 diabetes mellitus; TC, total cholesterol; TG, triglyceride

© 2023 Wang T et al. *JAMA Network Open.*

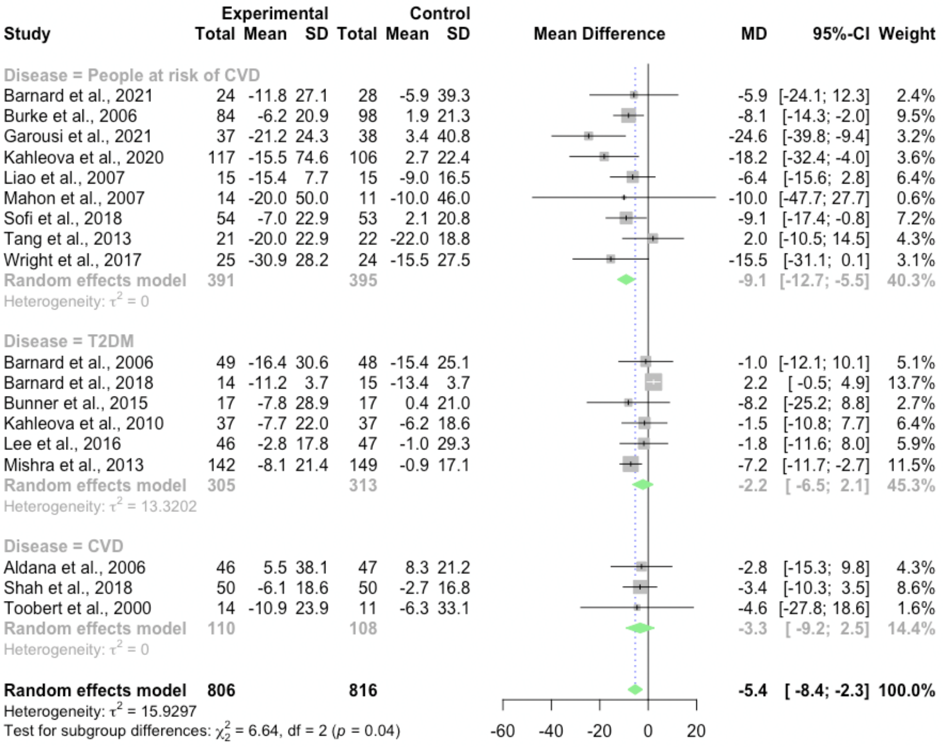

eFigure 1. Random effects model meta-analysis for changes in LDL-cholesterol concentrations comparing vegetarian diets intervention and all comparison diets, grouped by disease status of participants excluding Ornish *et al.*. CI, confidence interval; CVD, cardiovascular disease; MD, mean difference; SD, standard deviation; T2DM, type 2 diabetes mellitus.

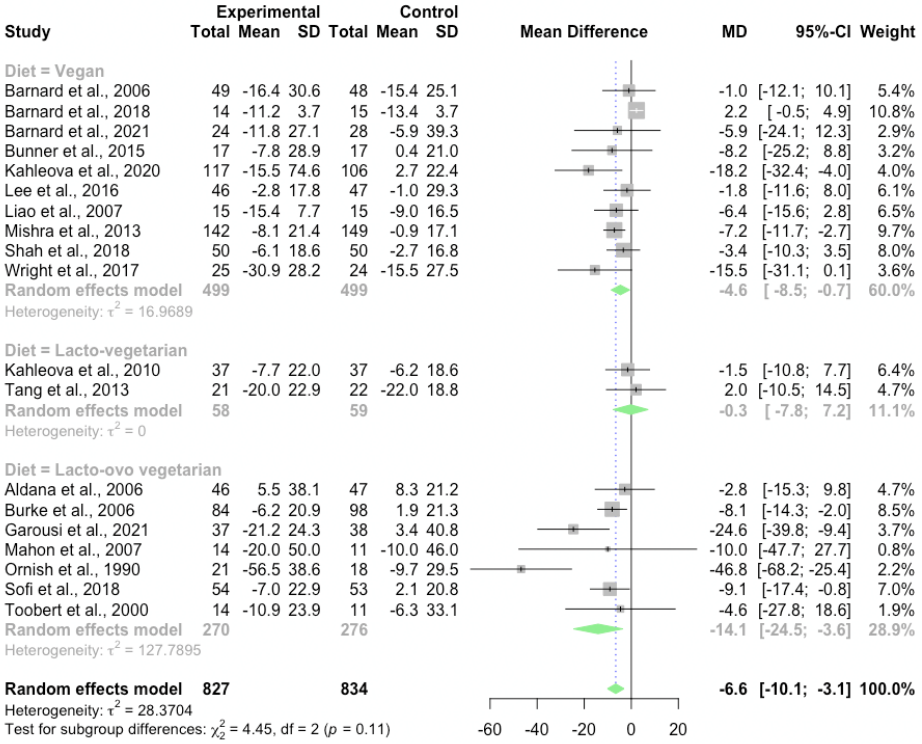

eFigure 2. Random effects model meta-analysis for changes in LDL-cholesterol concentrations comparing vegetarian diets intervention and all comparison diets, grouped by vegetarian diets. CI, confidence interval; MD, mean difference; SD, standard deviation.

© 2023 Wang T et al. *JAMA Network Open.*

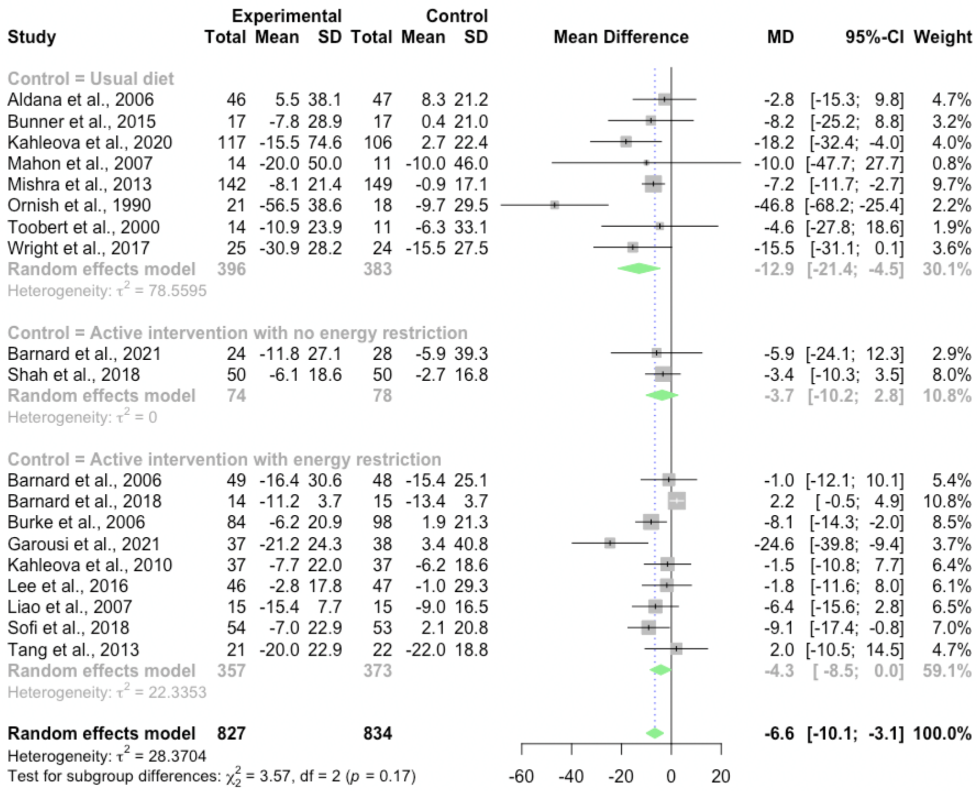

eFigure 3. Random effects model meta-analysis for changes in LDL-cholesterol concentrations comparing vegetarian diets intervention and all comparison diets, grouped by control diets. CI, confidence interval; MD, mean difference; SD, standard deviation.

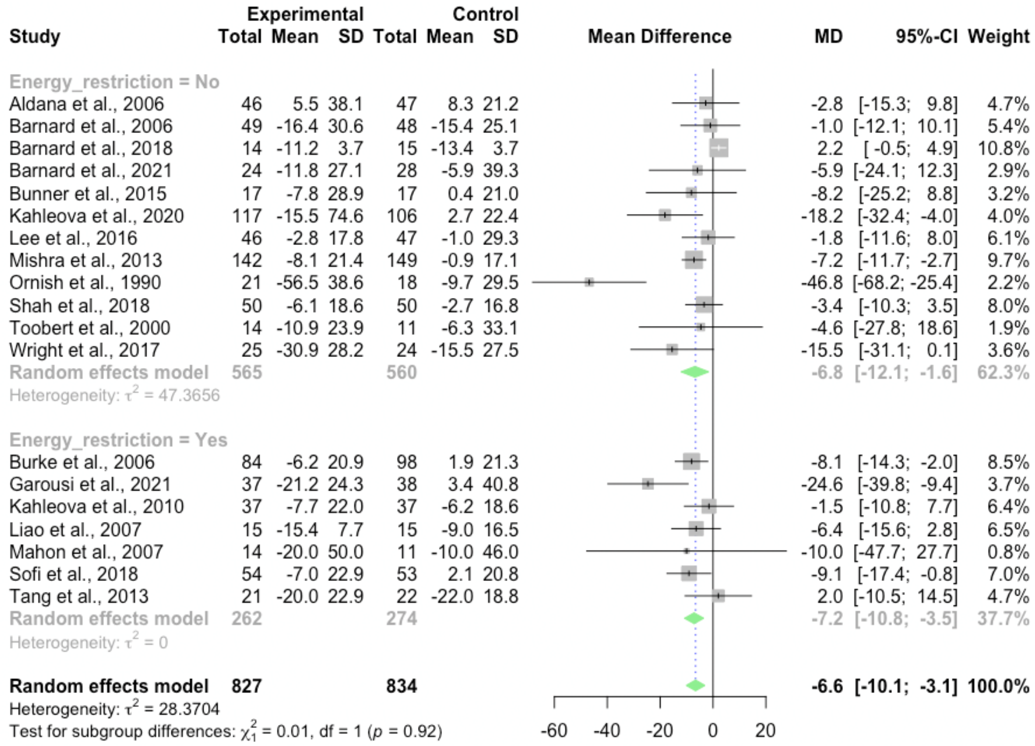

eFigure 4. Random effects model meta-analysis for changes in LDL-cholesterol concentrations comparing vegetarian diets intervention and all comparison diets, grouped by energy restriction. CI, confidence interval; MD, mean difference; SD, standard deviation.

© 2023 Wang T et al. *JAMA Network Open.*

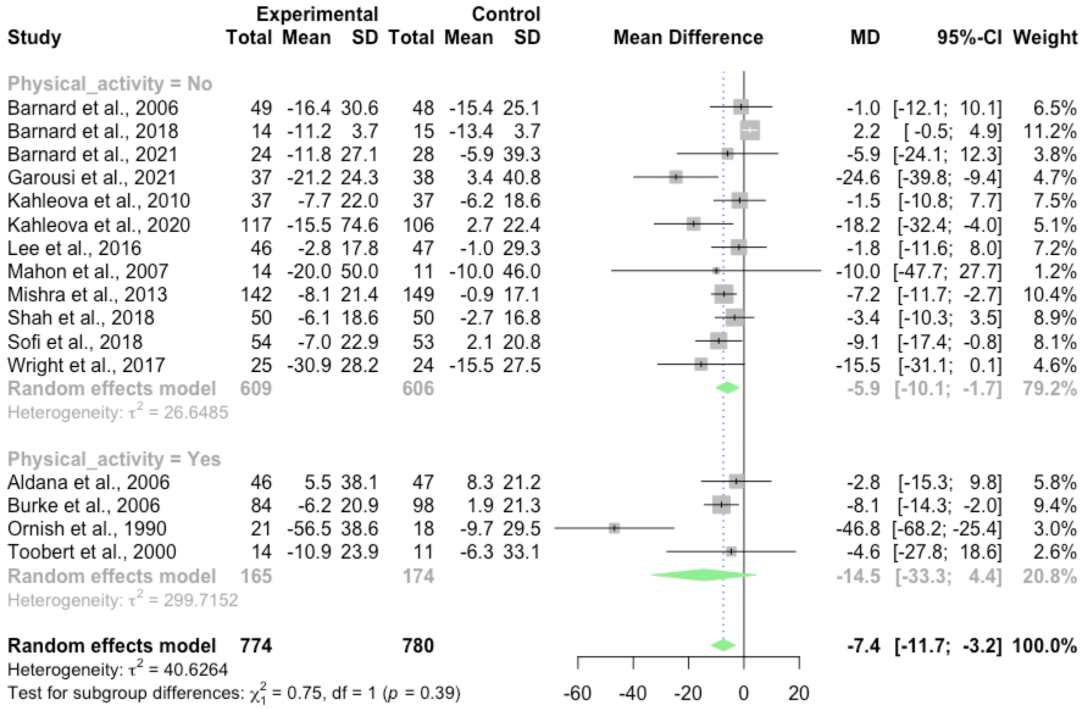

eFigure 5. Random effects model meta-analysis for changes in LDL-cholesterol concentrations comparing vegetarian diets intervention and all comparison diets, grouped by physical activity. CI, confidence interval; MD, mean difference; SD, standard deviation.

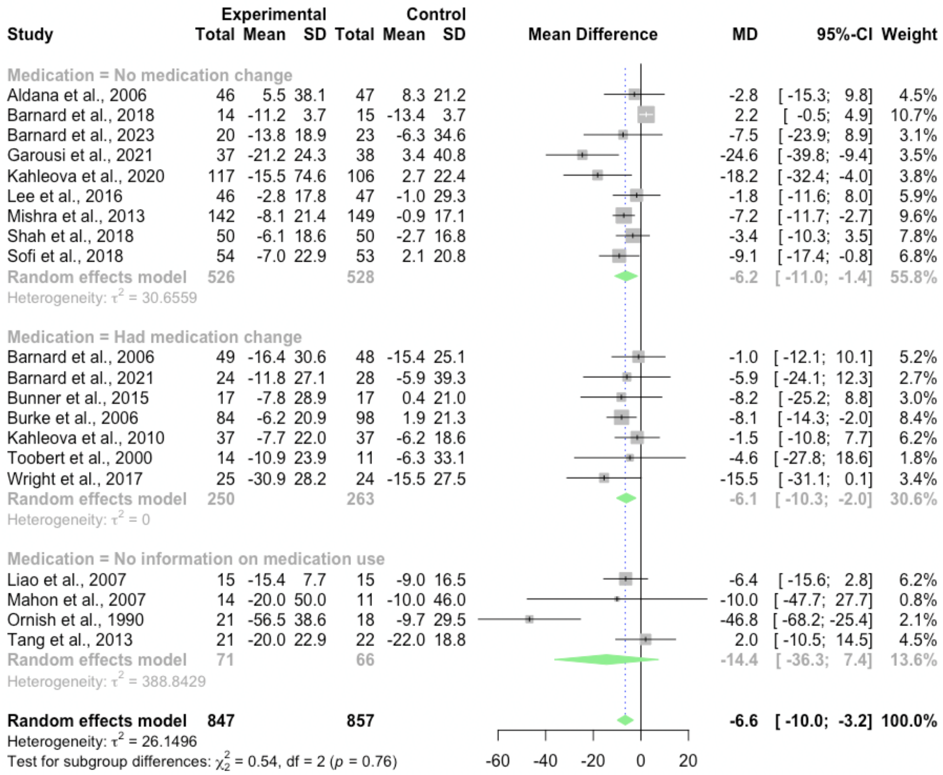

eFigure 6. Random effects model meta-analysis for changes in LDL-cholesterol concentrations comparing vegetarian diets intervention and all comparison diets, grouped by medication use. CI, confidence interval; MD, mean difference; SD, standard deviation.

© 2023 Wang T et al. *JAMA Network Open.*

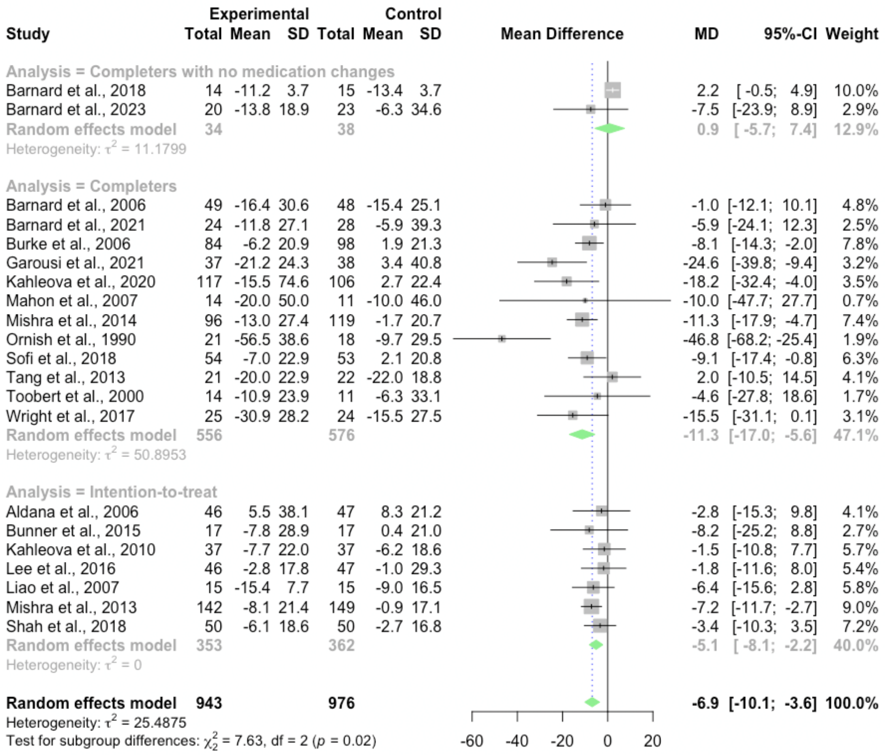

eFigure 7. Random effects model meta-analysis for changes in LDL-cholesterol concentrations comparing vegetarian diets intervention and all comparison diets, grouped by analysis method. CI, confidence interval; MD, mean difference; SD, standard deviation.

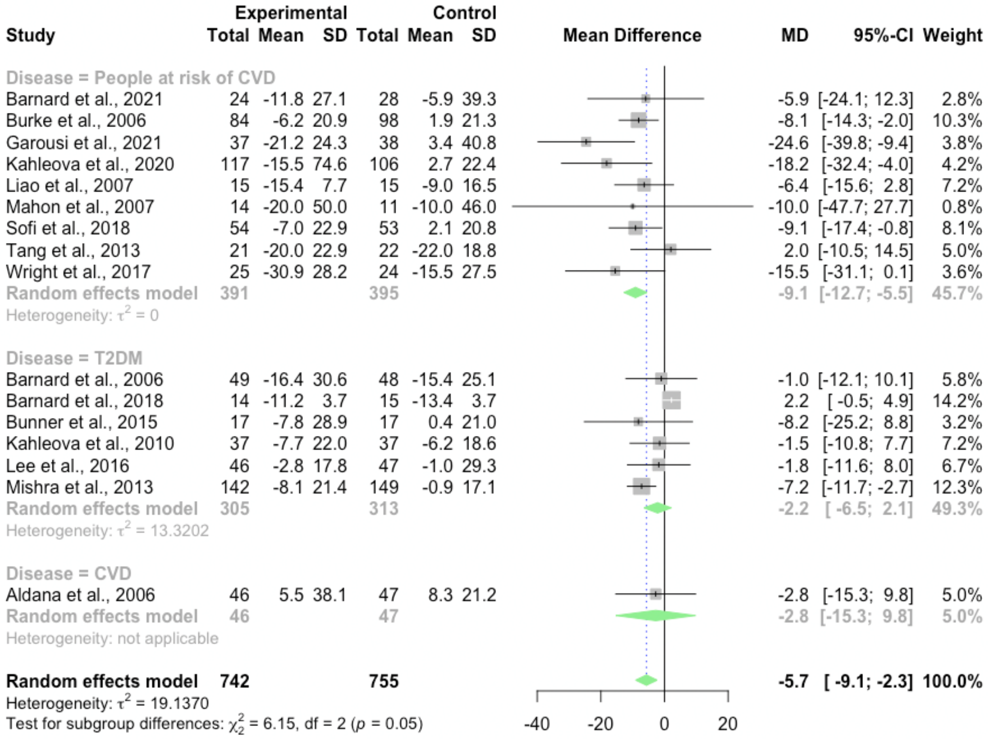

eFigure 8. Random effects model meta-analysis for changes in LDL-cholesterol concentrations comparing vegetarian diets intervention and all comparison diets, grouped by disease status of participants excluding imputed data. CI, confidence interval; CVD, cardiovascular disease; MD, mean difference; SD, standard deviation; T2DM, type 2 diabetes mellitus.

© 2023 Wang T et al. *JAMA Network Open.*

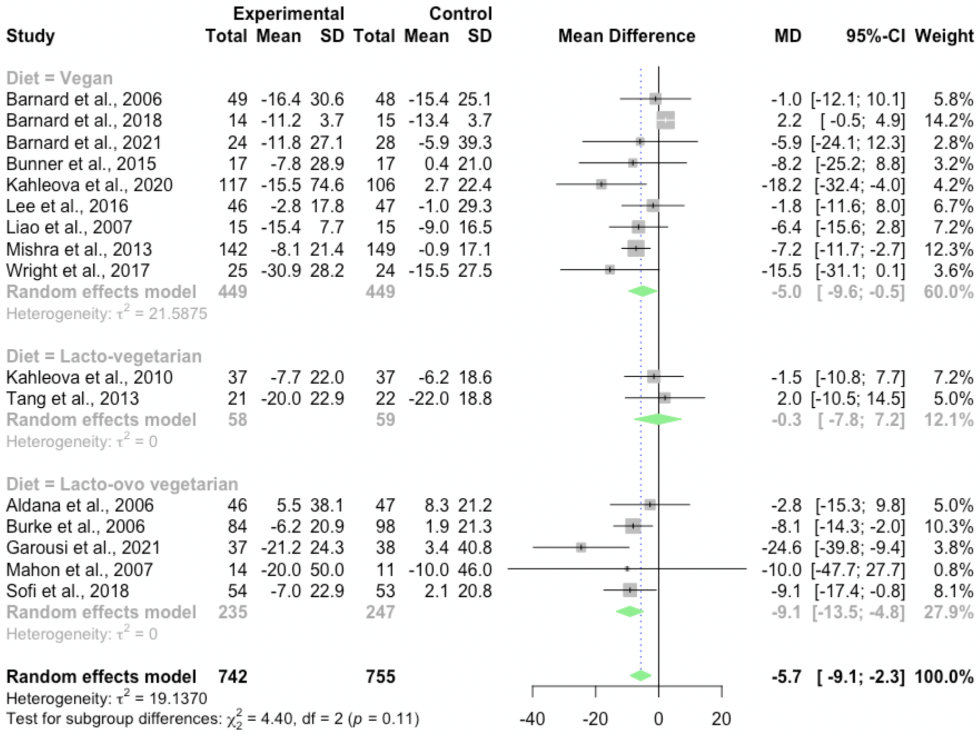

eFigure 9. Random effects model meta-analysis for changes in LDL-cholesterol concentrations comparing vegetarian diets intervention and all comparison diets, grouped by various vegetarian diets excluding imputed data. CI, confidence interval; MD, mean difference; SD, standard deviation.

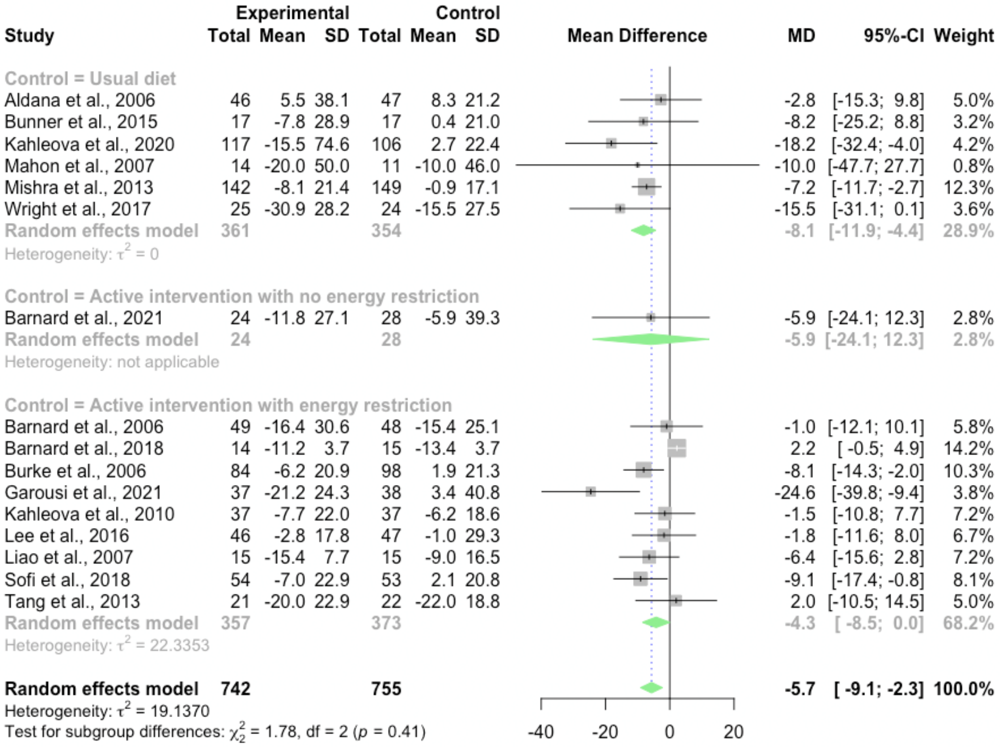

eFigure 10. Random effects model meta-analysis for changes in LDL-cholesterol concentrations comparing vegetarian diets intervention and all comparison diets, grouped by control diets excluding imputed data. CI, confidence interval; MD, mean difference; SD, standard deviation.

© 2023 Wang T et al. *JAMA Network Open.*

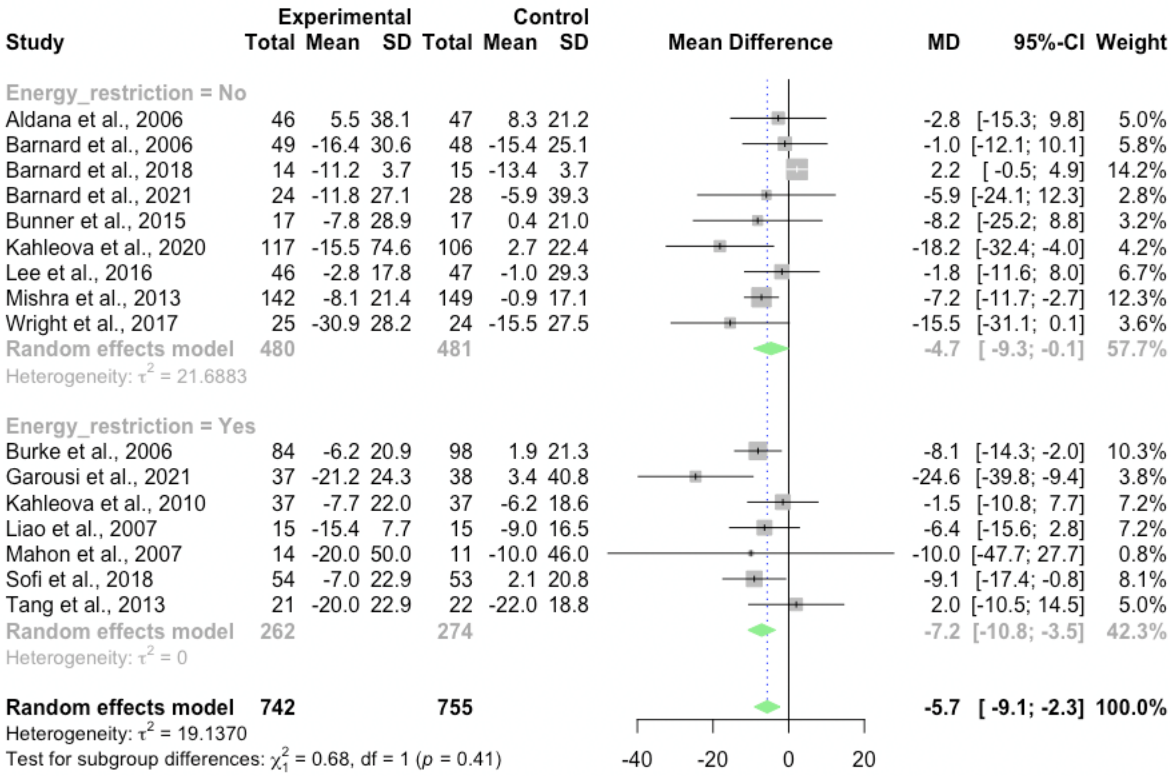

eFigure 11. Random effects model meta-analysis for changes in LDL-cholesterol concentrations comparing vegetarian diets intervention and all comparison diets, grouped by energy restriction excluding imputed data. CI, confidence interval; MD, mean difference; SD, standard deviation.

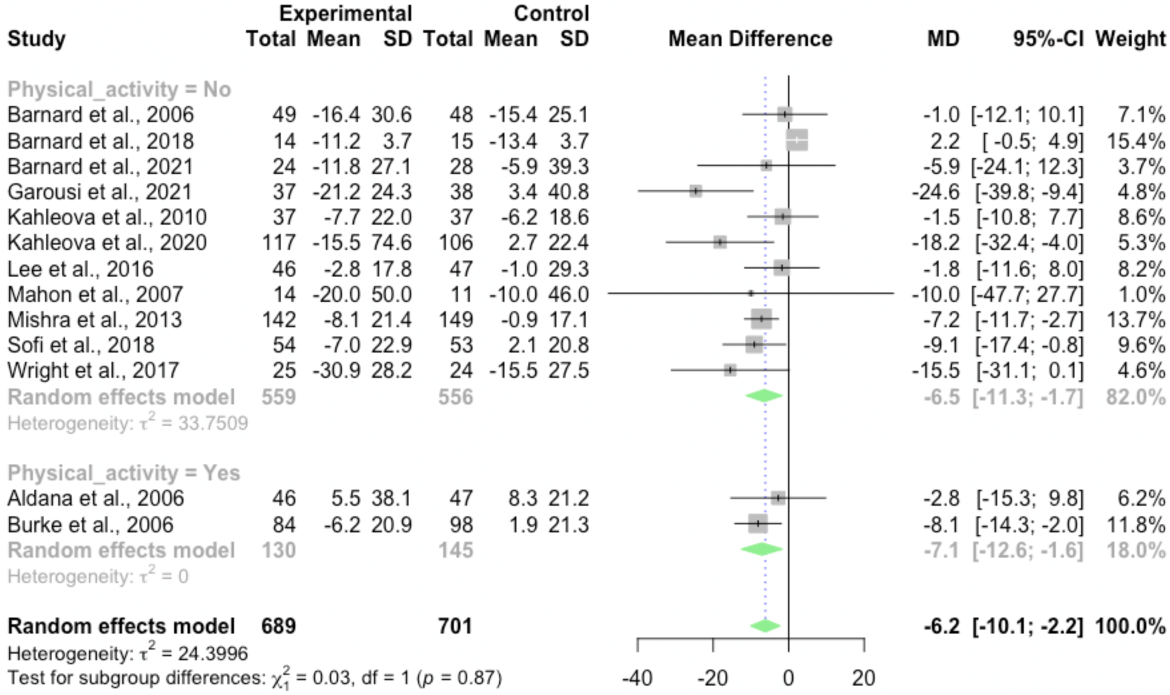

eFigure 12. Random effects model meta-analysis for changes in LDL-cholesterol concentrations comparing vegetarian diets intervention and all comparison diets, grouped by physical activity excluding imputed data. CI, confidence interval; MD, mean difference; SD, standard deviation.

© 2023 Wang T et al. *JAMA Network Open.*

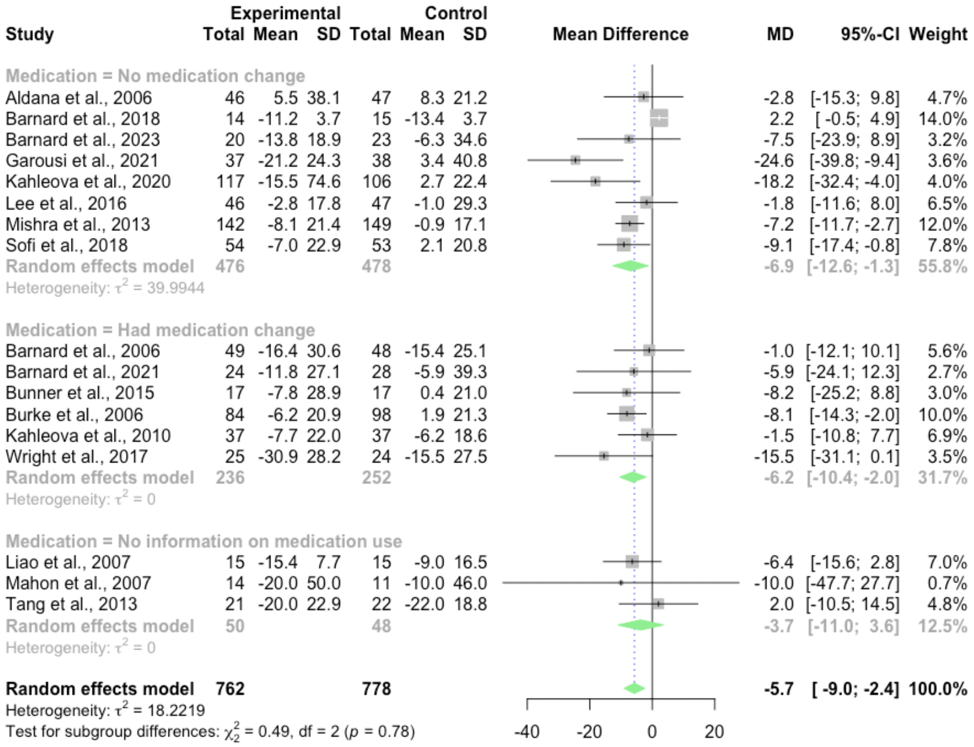

eFigure 13. Random effects model meta-analysis for changes in LDL-cholesterol concentrations comparing vegetarian diets intervention and all comparison diets, grouped by medication use excluding imputed data. CI, confidence interval; MD, mean difference; SD, standard deviation.

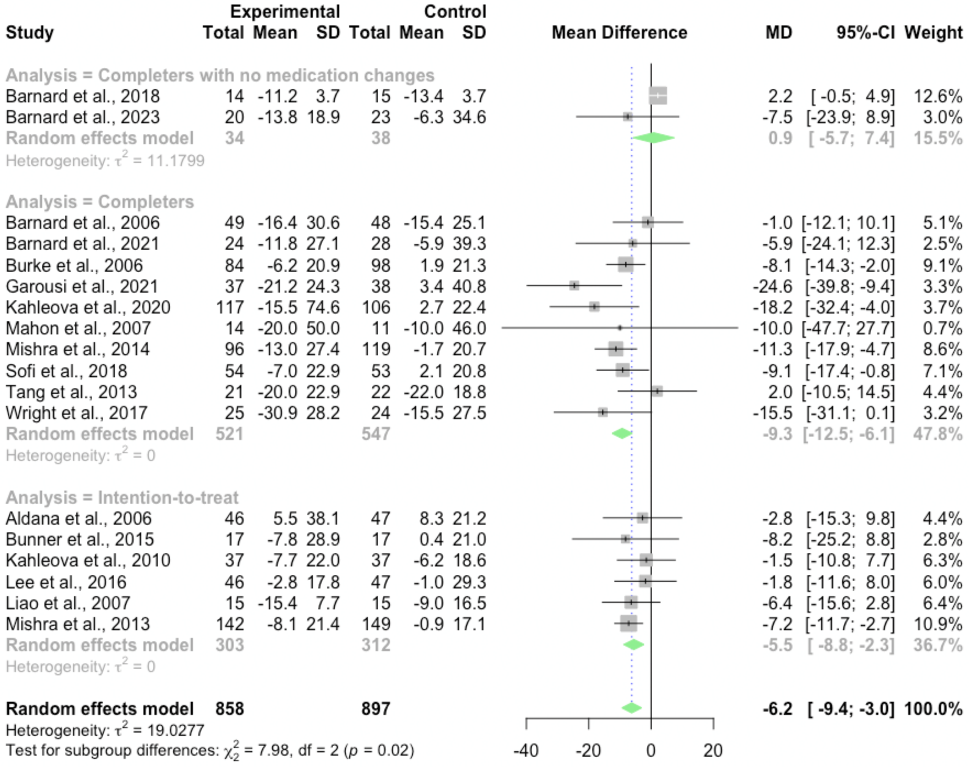

eFigure 14. Random effects model meta-analysis for changes in LDL-cholesterol concentrations comparing vegetarian diets intervention and all comparison diets, grouped by analysis method excluding imputed data. CI, confidence interval; MD, mean difference; SD, standard deviation.

© 2023 Wang T et al. *JAMA Network Open.*

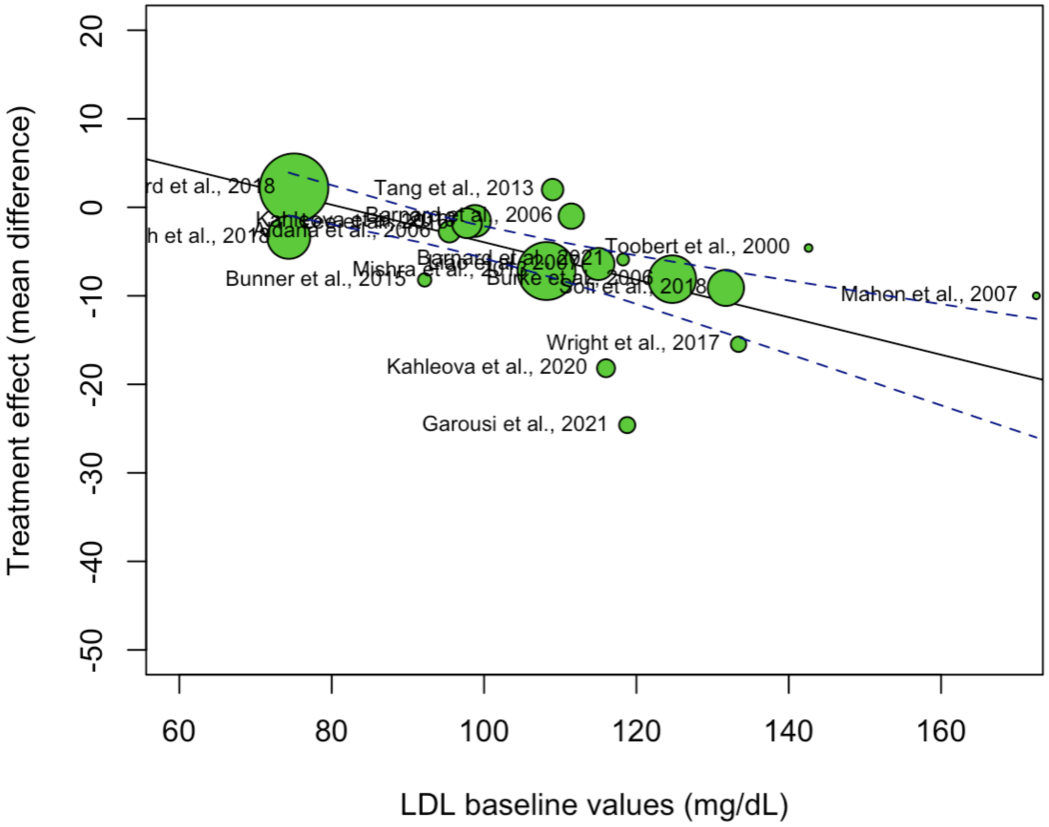

eFigure 15. Treatment effect of LDL-C across increasing levels of baseline LDL-C (mg/dL)

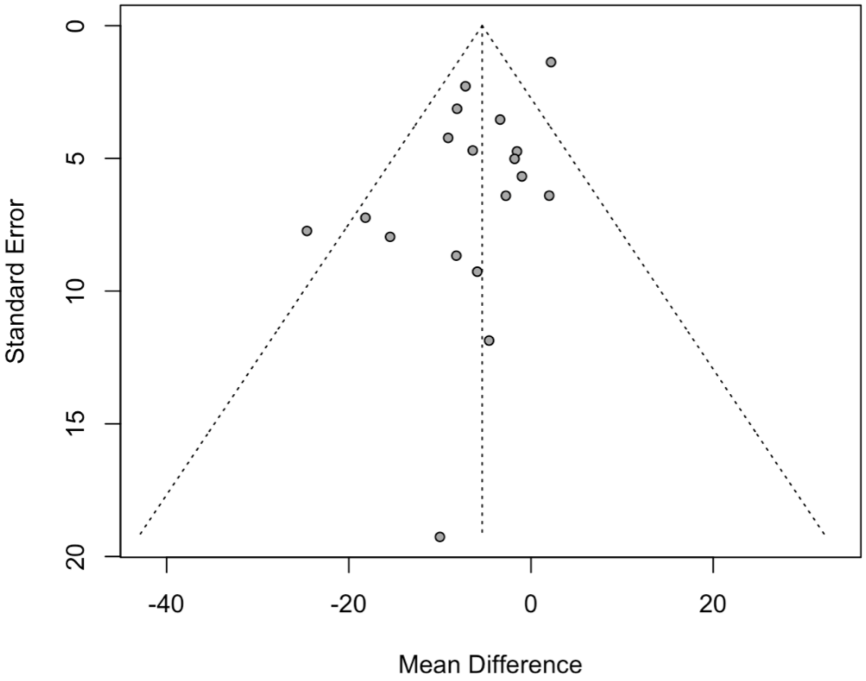

eFigure 16. Funnel plot of LDL-C for risk of publication bias. Mean change in LDL-C plotted against the SE of the mean change. The plot appears fairly symmetrical.

© 2023 Wang T et al. *JAMA Network Open.*

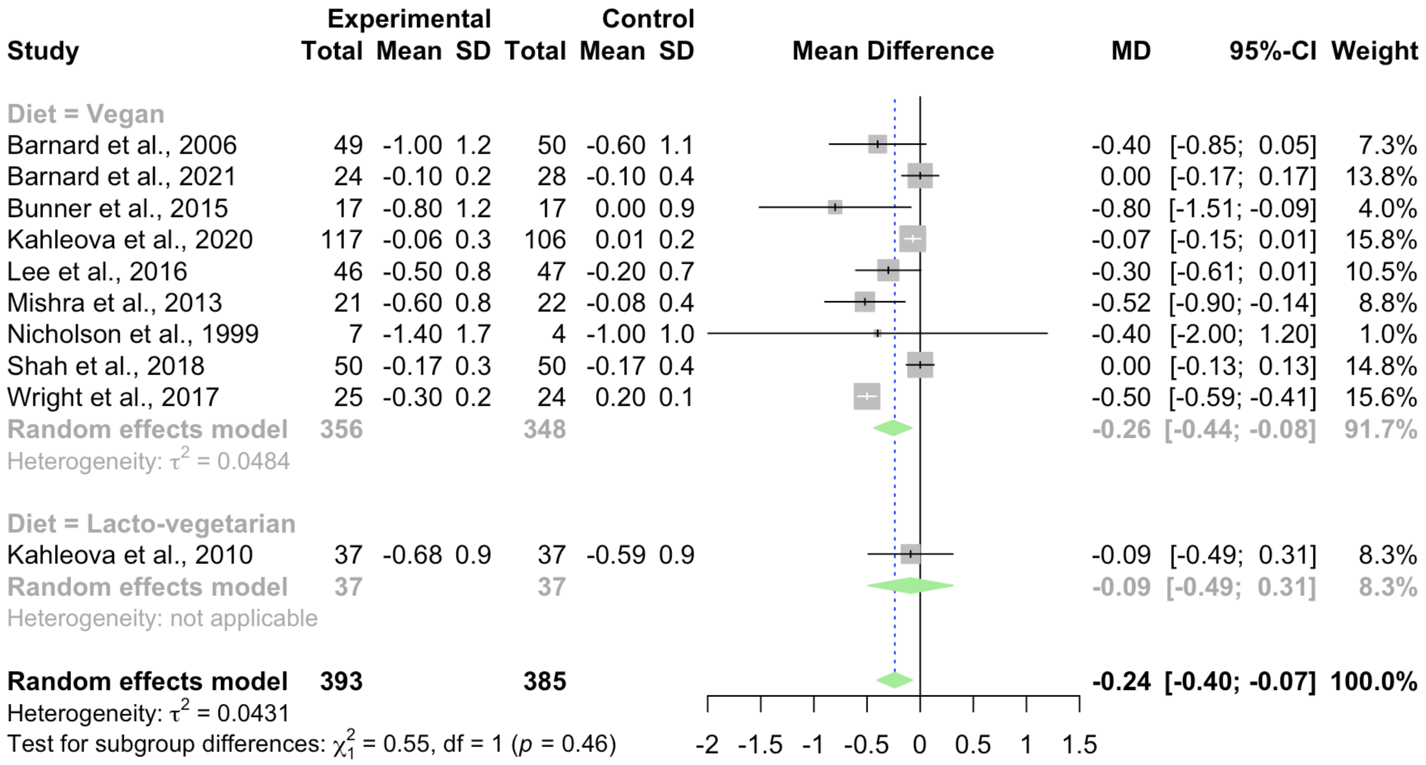

eFigure 17. Random effects model meta-analysis for changes in HbA1c comparing vegetarian diets intervention and all comparison diets, grouped by vegetarian diets. CI, confidence interval; CVD, cardiovascular disease; MD, mean difference; SD, standard deviation; T2DM, type 2 diabetes mellitus.

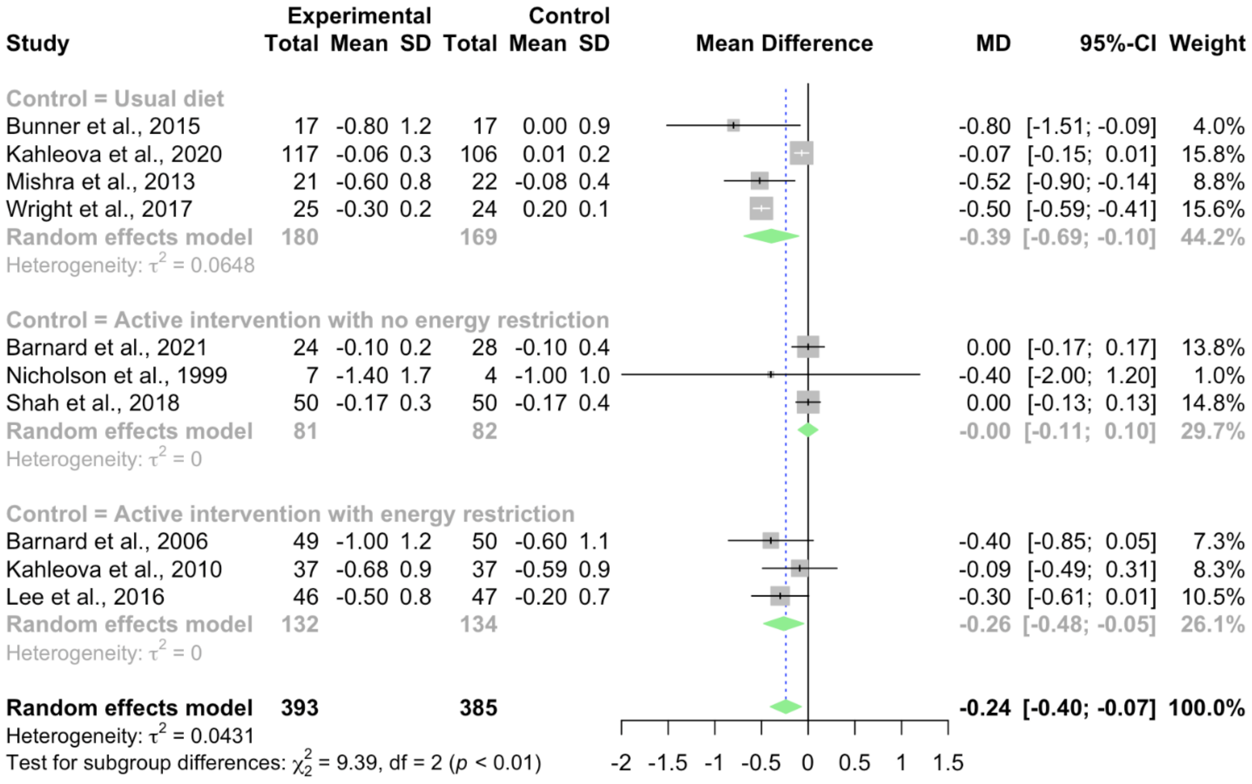

eFigure 18. Random effects model meta-analysis for changes in HbA1c comparing vegetarian diets intervention and all comparison diets, grouped by control diets. CI, confidence interval; MD, mean difference; SD, standard deviation.

© 2023 Wang T et al. *JAMA Network Open.*

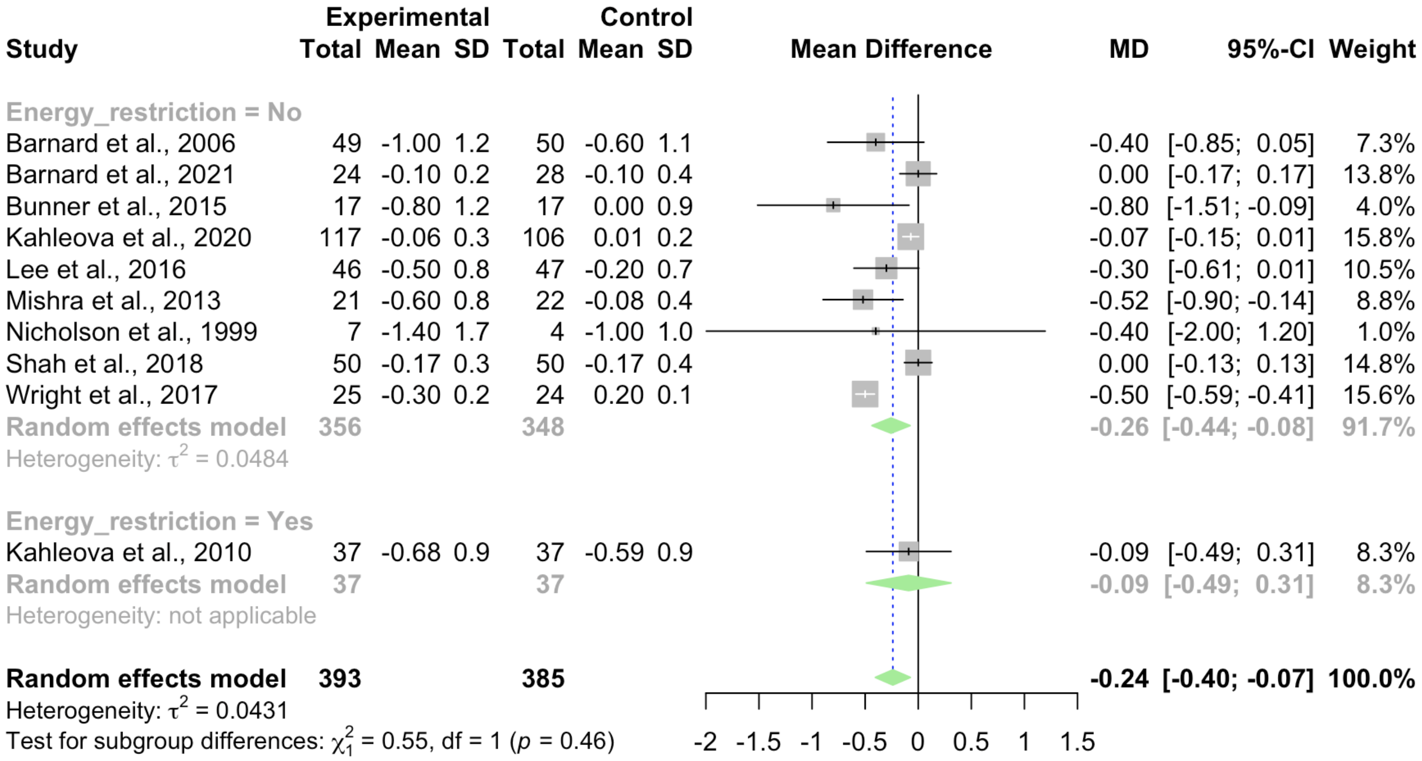

eFigure 19. Random effects model meta-analysis for changes in HbA1c comparing vegetarian diets intervention and all comparison diets, grouped by energy restriction. CI, confidence interval; MD, mean difference; SD, standard deviation.

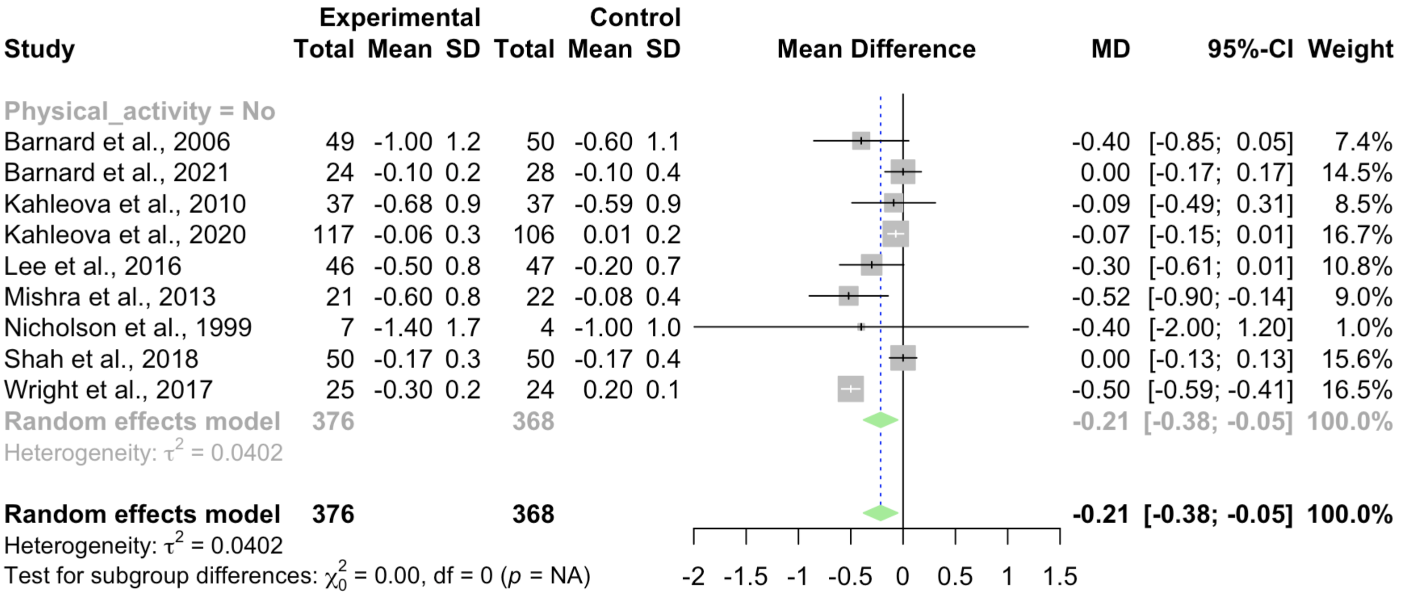

eFigure 20. Random effects model meta-analysis for changes in HbA1c comparing vegetarian diets intervention and all comparison diets, grouped by physical activity. CI, confidence interval; MD, mean difference; SD, standard deviation.

© 2023 Wang T et al. *JAMA Network Open.*

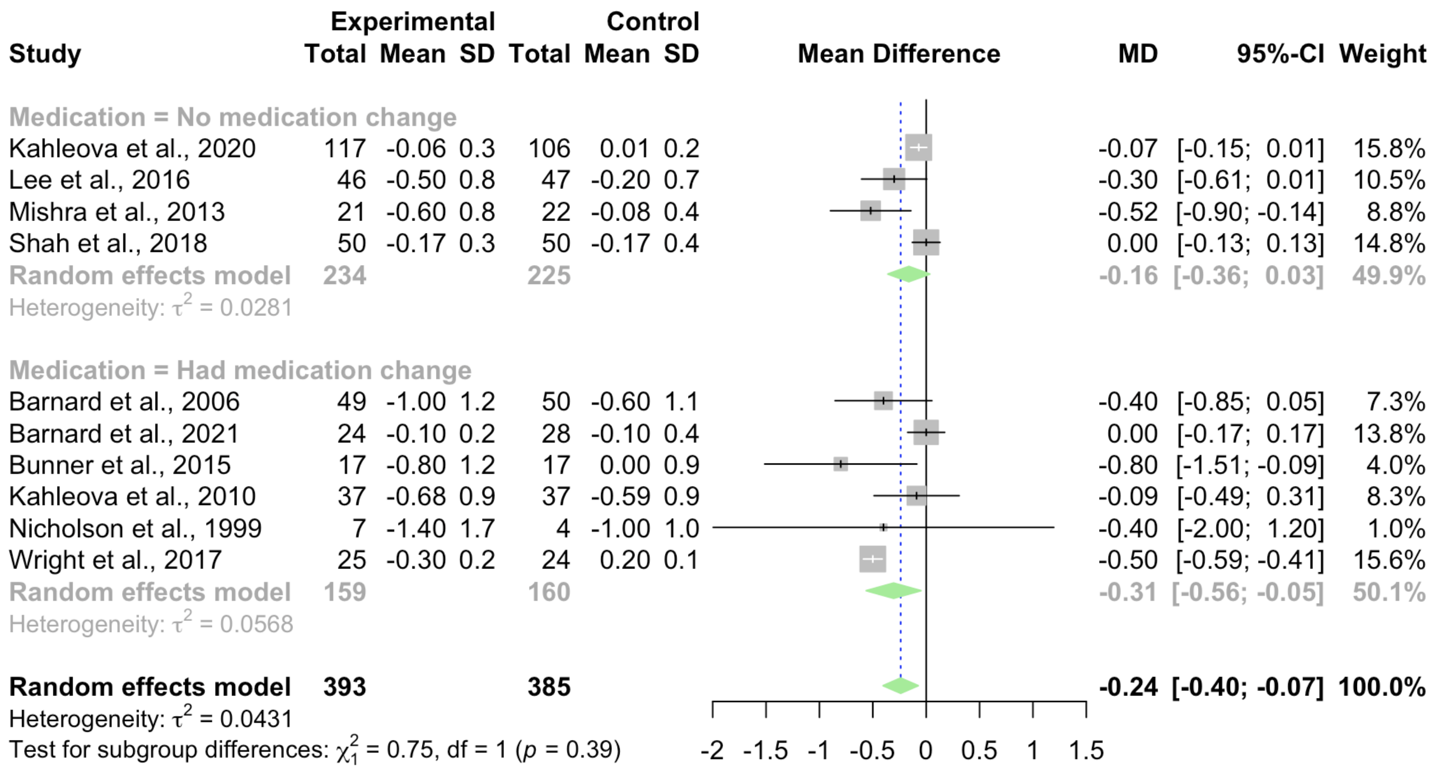

eFigure 21. Random effects model meta-analysis for changes in HbA1c comparing vegetarian diets intervention and all comparison diets, grouped by medication use. CI, confidence interval; MD, mean difference; SD, standard deviation.

© 2023 Wang T et al. *JAMA Network Open.*

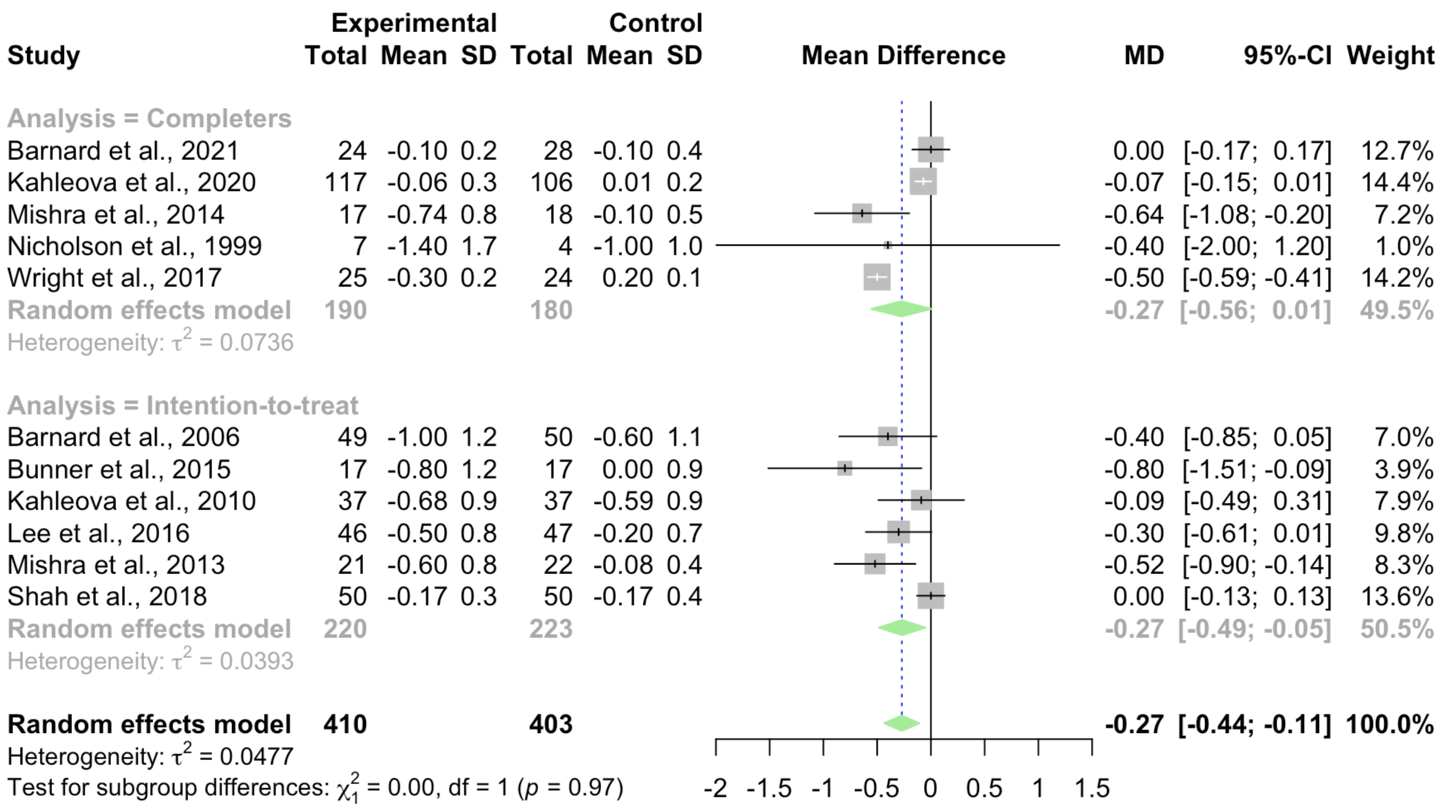

eFigure 22. Random effects model meta-analysis for changes in HbA1c comparing vegetarian diets intervention and all comparison diets, grouped by analysis method. CI, confidence interval; MD, mean difference; SD, standard deviation.

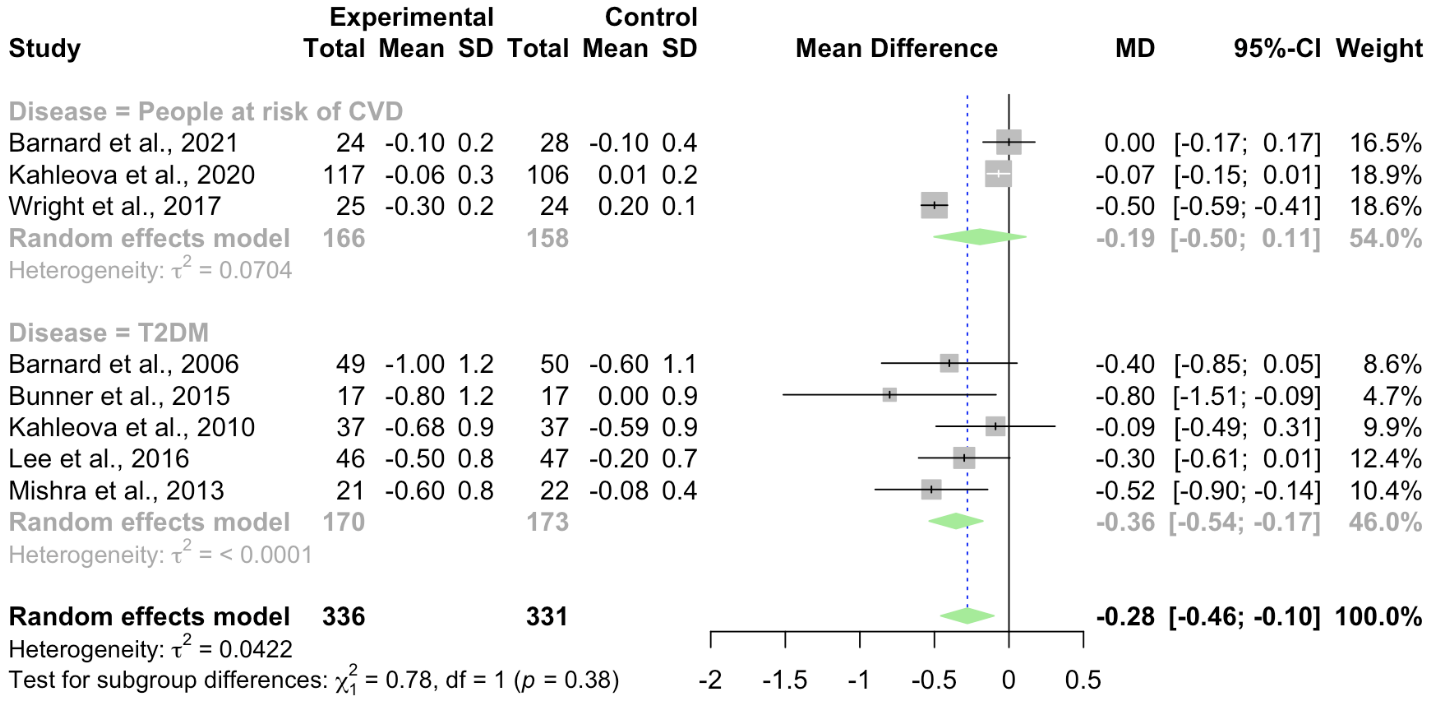

eFigure 23. Random effects model meta-analysis for changes in HbA1c comparing vegetarian diets intervention and all comparison diets, grouped by disease status of participants excluding imputed data. CI, confidence interval; CVD, cardiovascular disease; MD, mean difference; SD, standard deviation; T2DM, type 2 diabetes mellitus.

© 2023 Wang T et al. *JAMA Network Open.*

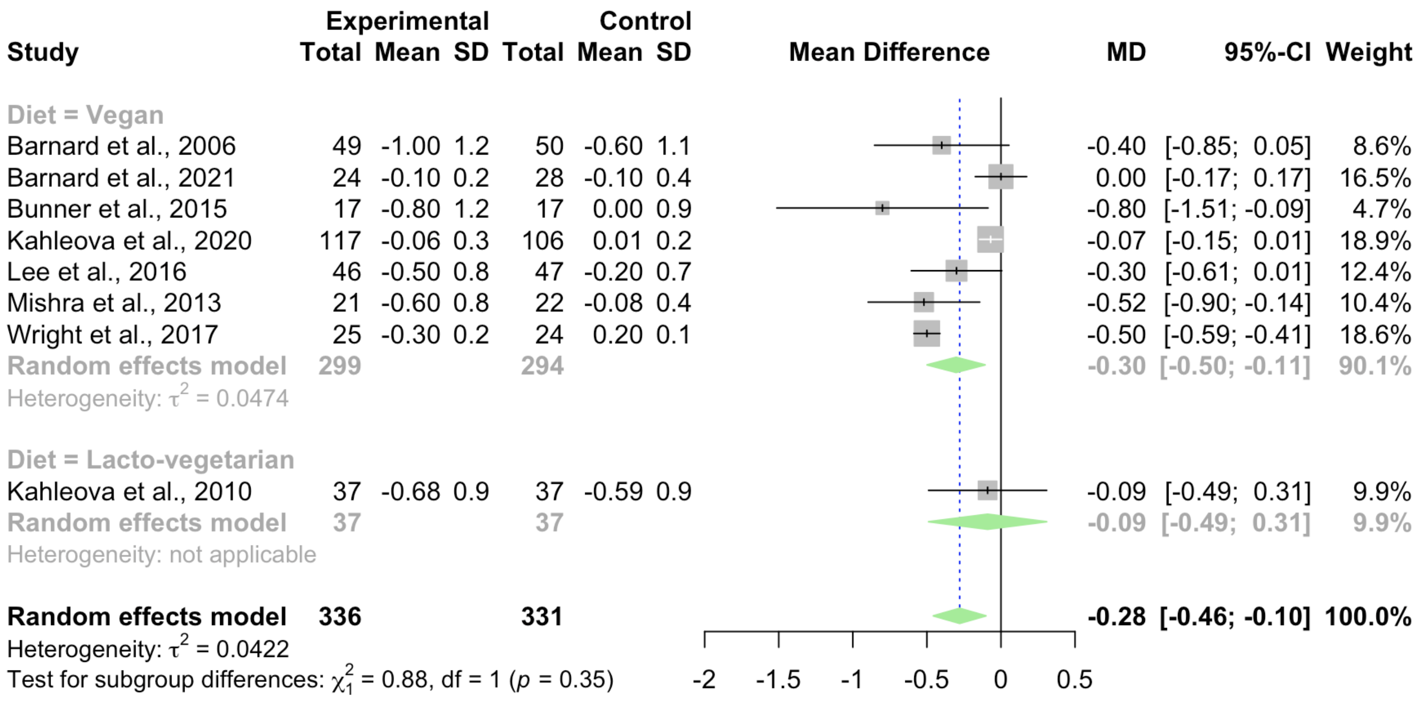

eFigure 24. Random effects model meta-analysis for changes in HbA1c comparing vegetarian diets intervention and all comparison diets, grouped by various vegetarian diets excluding imputed data. CI, confidence interval; MD, mean difference; SD, standard deviation.

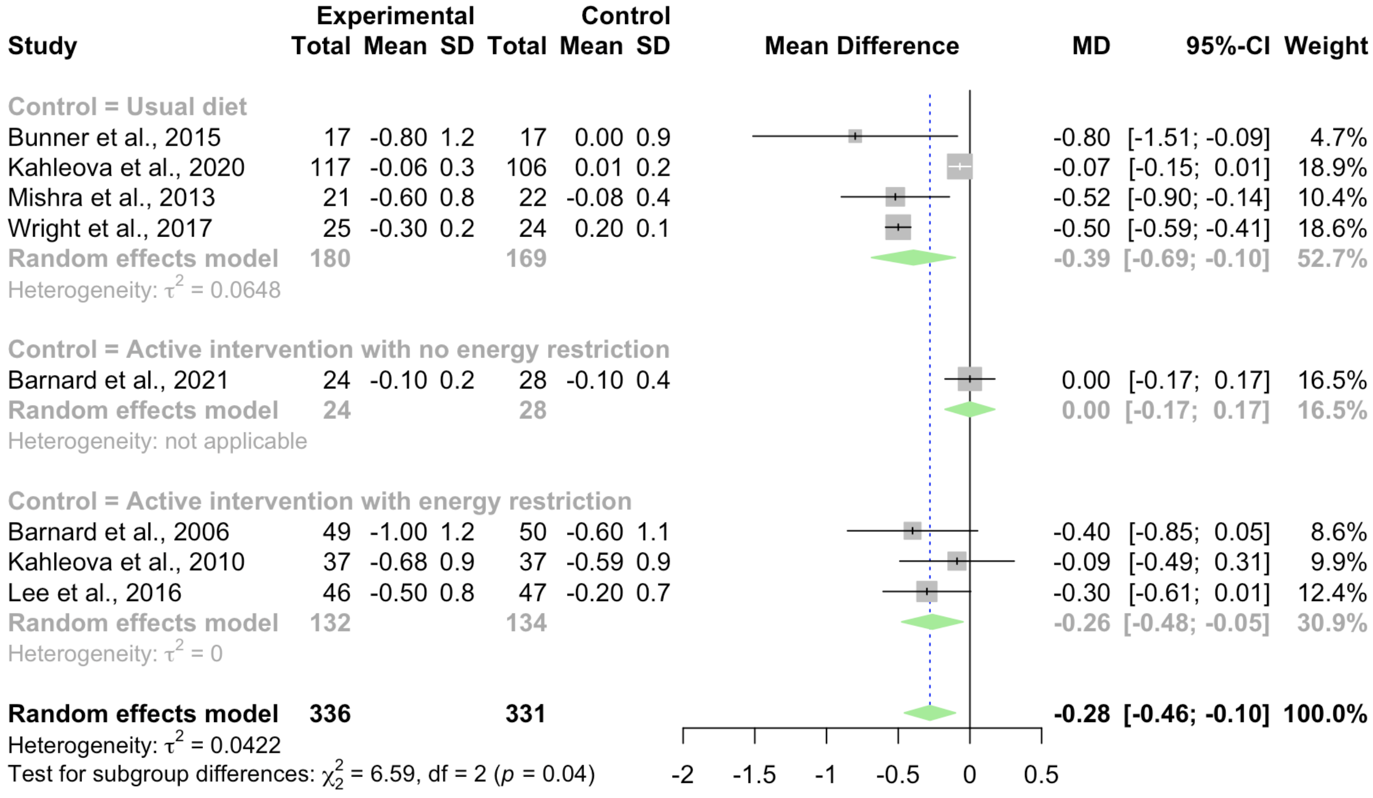

eFigure 25. Random effects model meta-analysis for changes in HbA1c comparing vegetarian diets intervention and all comparison diets, grouped by control diets excluding imputed data. CI, confidence interval; MD, mean difference; SD, standard deviation.

© 2023 Wang T et al. *JAMA Network Open.*

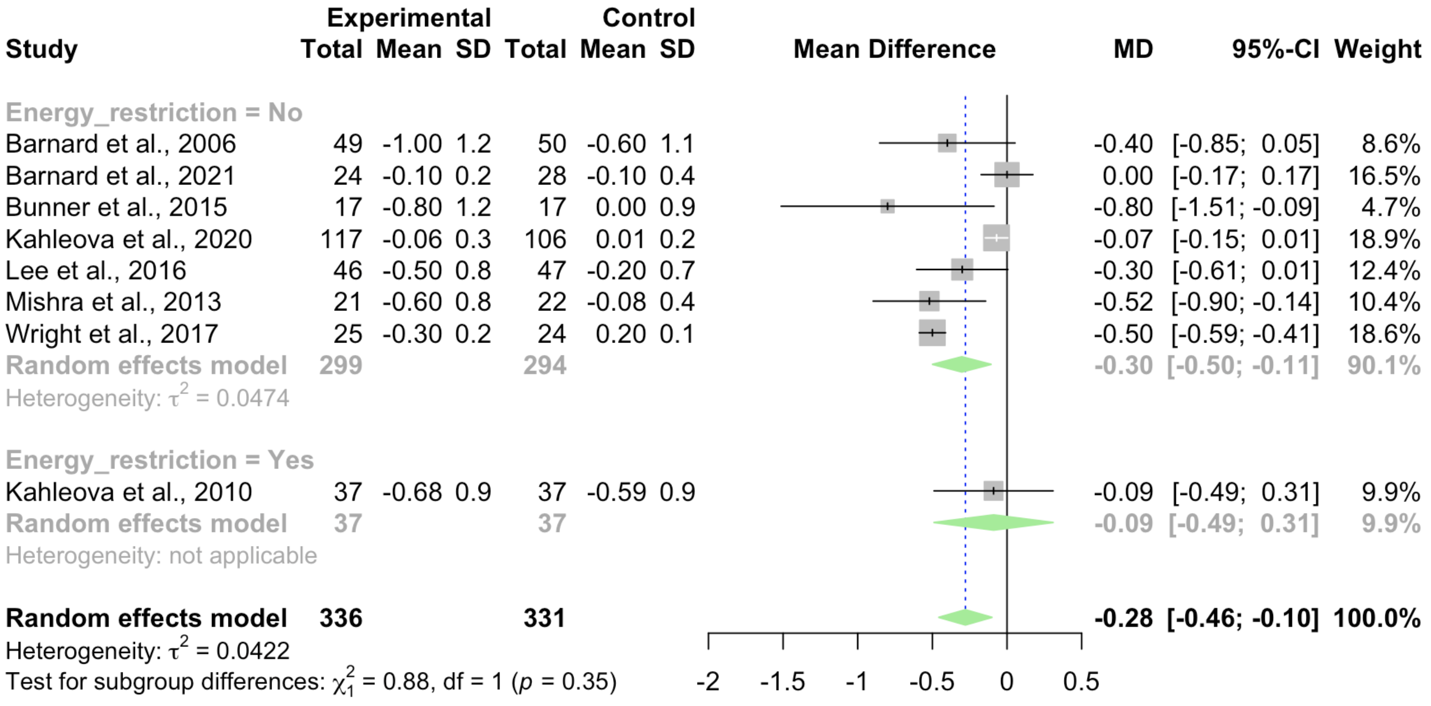

eFigure 26. Random effects model meta-analysis for changes in HbA1c comparing vegetarian diets intervention and all comparison diets, grouped by energy restriction excluding imputed data. CI, confidence interval; MD, mean difference; SD, standard deviation.

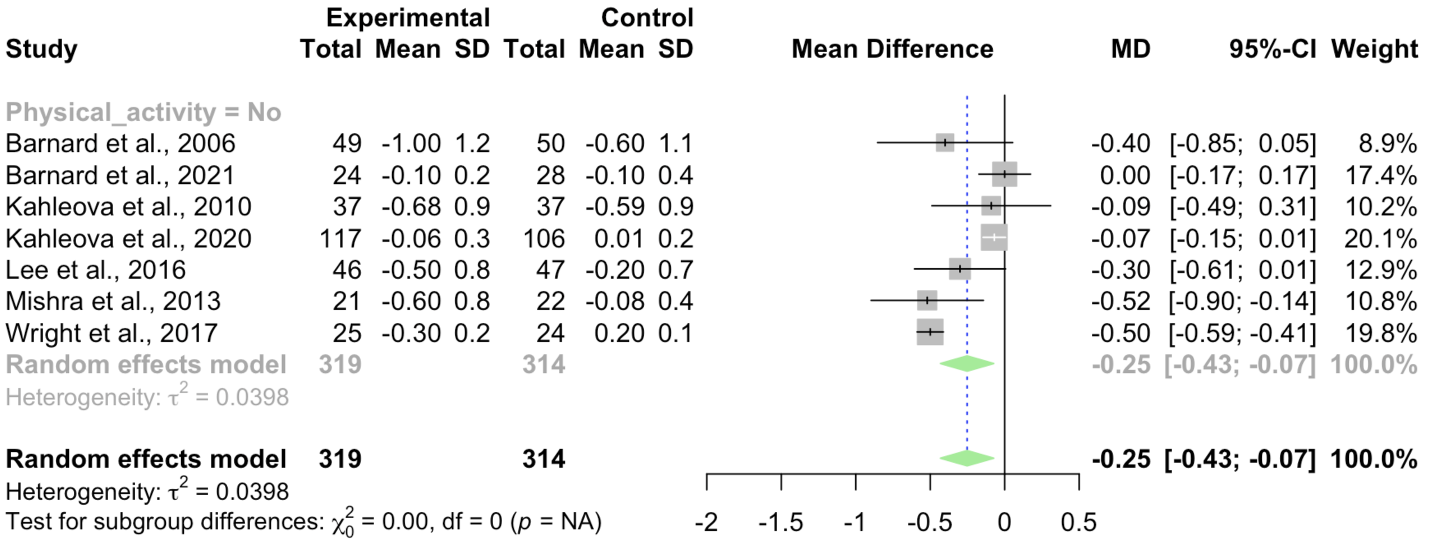

eFigure 27. Random effects model meta-analysis for changes in HbA1c comparing vegetarian diets intervention and all comparison diets, grouped by physical activity excluding imputed data. CI, confidence interval; MD, mean difference; SD, standard deviation.

© 2023 Wang T et al. *JAMA Network Open.*

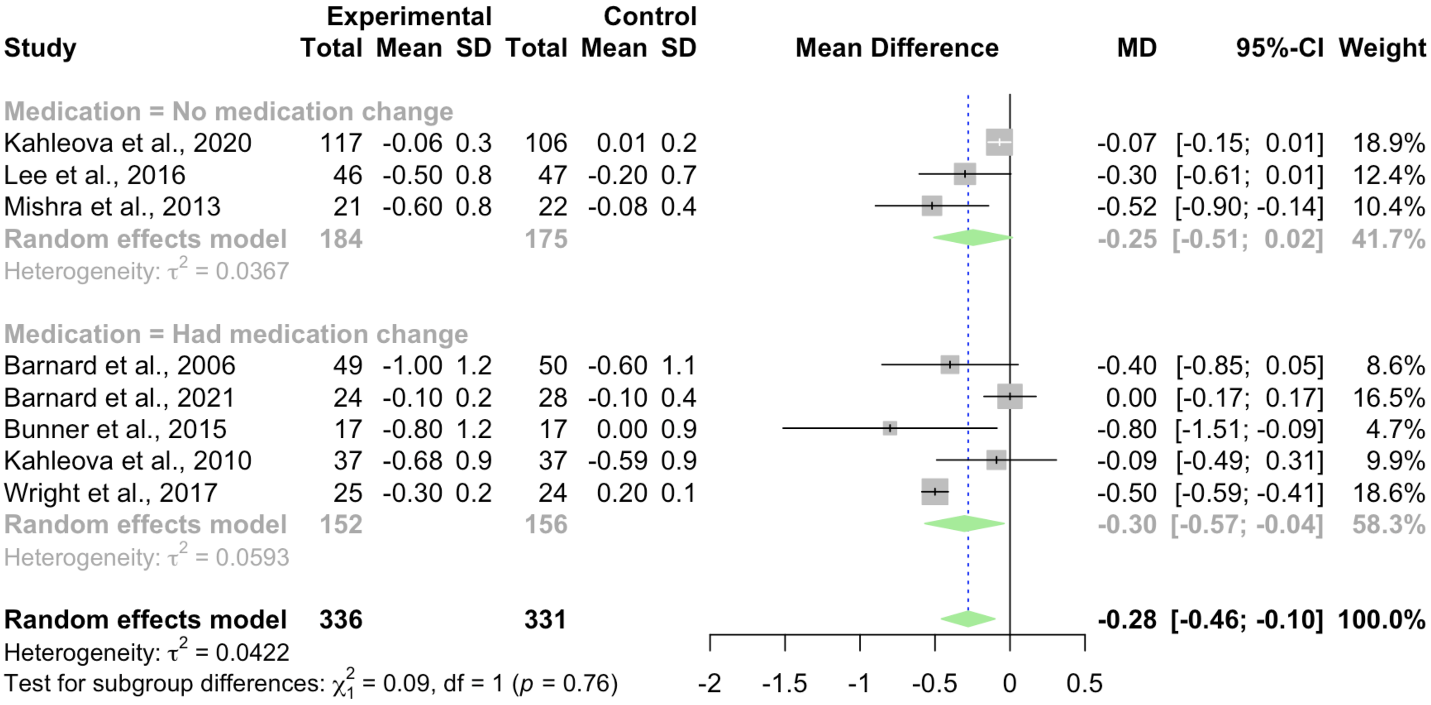

eFigure 28. Random effects model meta-analysis for changes in HbA1c comparing vegetarian diets intervention and all comparison diets, grouped by medication use excluding imputed data. CI, confidence interval; MD, mean difference; SD, standard deviation.

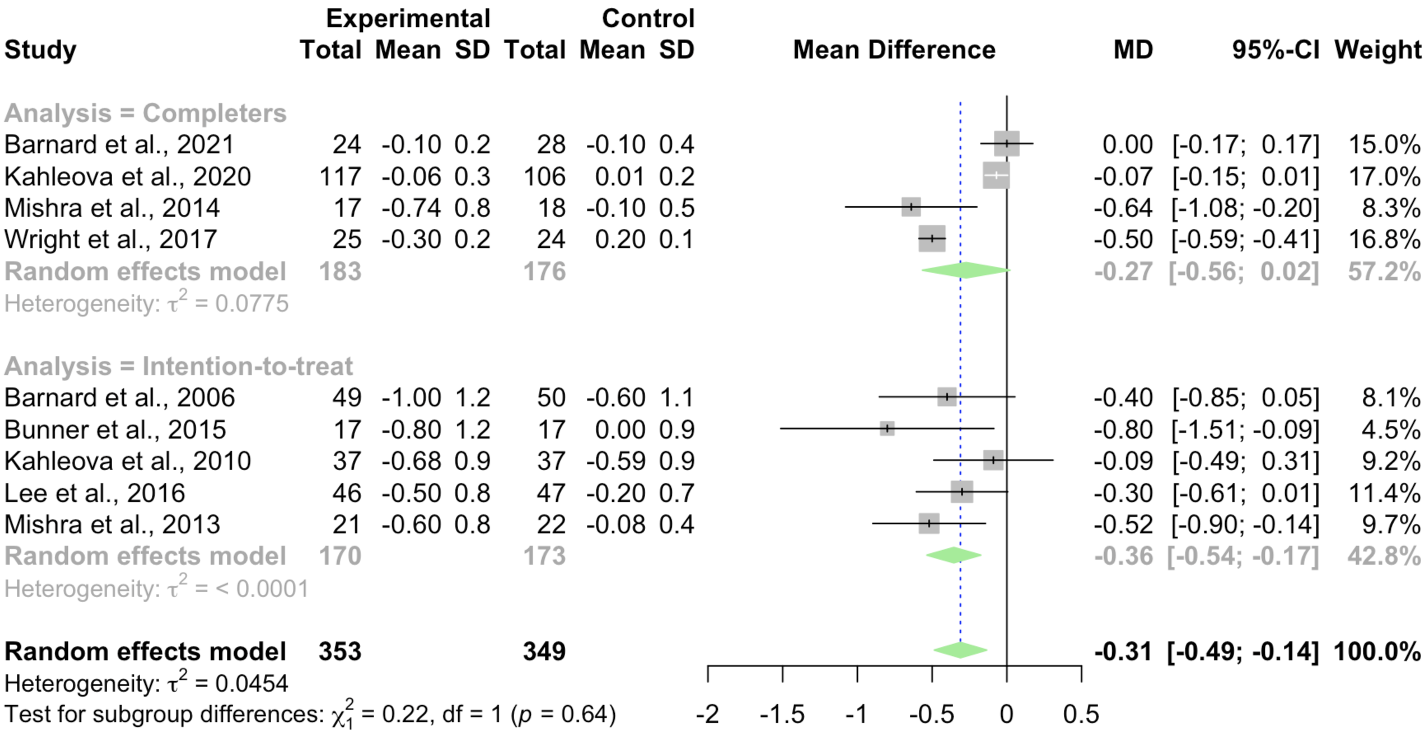

eFigure 29. Random effects model meta-analysis for changes in HbA1c comparing vegetarian diets intervention and all comparison diets, grouped by analysis method excluding imputed data. CI, confidence interval; MD, mean difference; SD, standard deviation.

© 2023 Wang T et al. *JAMA Network Open.*

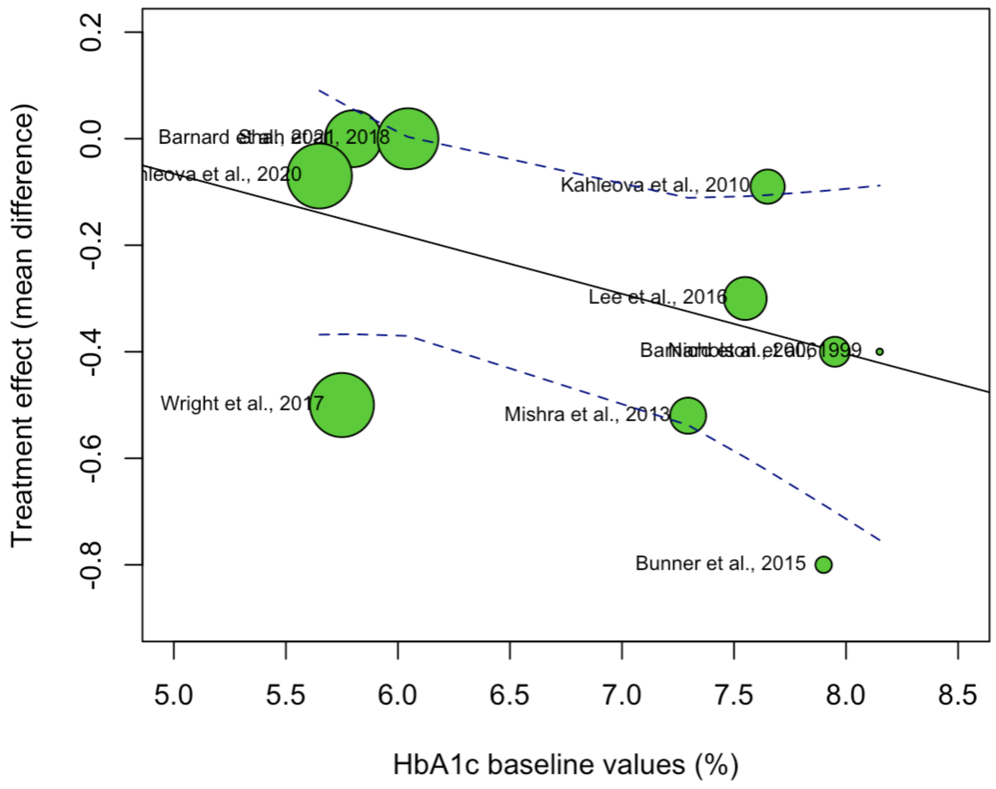

eFigure 30. Treatment effect of HbA1c across increasing levels of baseline HbA1c (%)

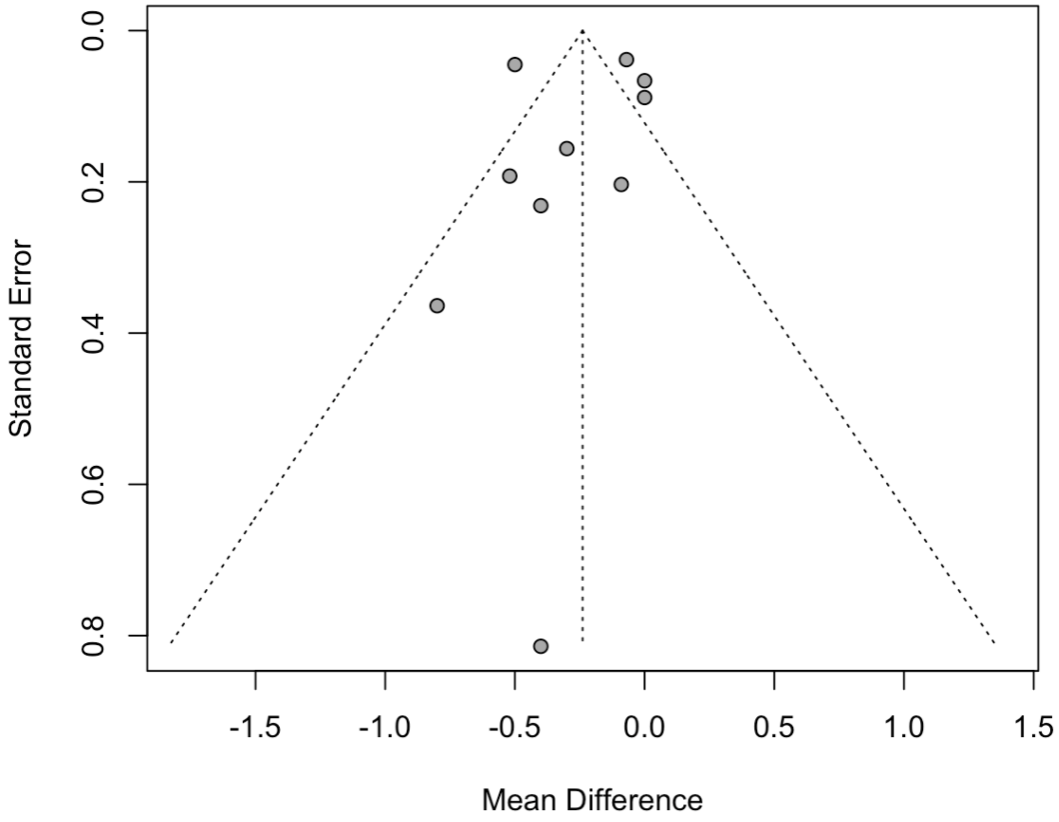

eFigure 31. Funnel plot of HbA1c for risk of publication bias. Mean change in HbA1c plotted against the SE of the mean change. The plot appears fairly symmetrical.

© 2023 Wang T et al. *JAMA Network Open.*

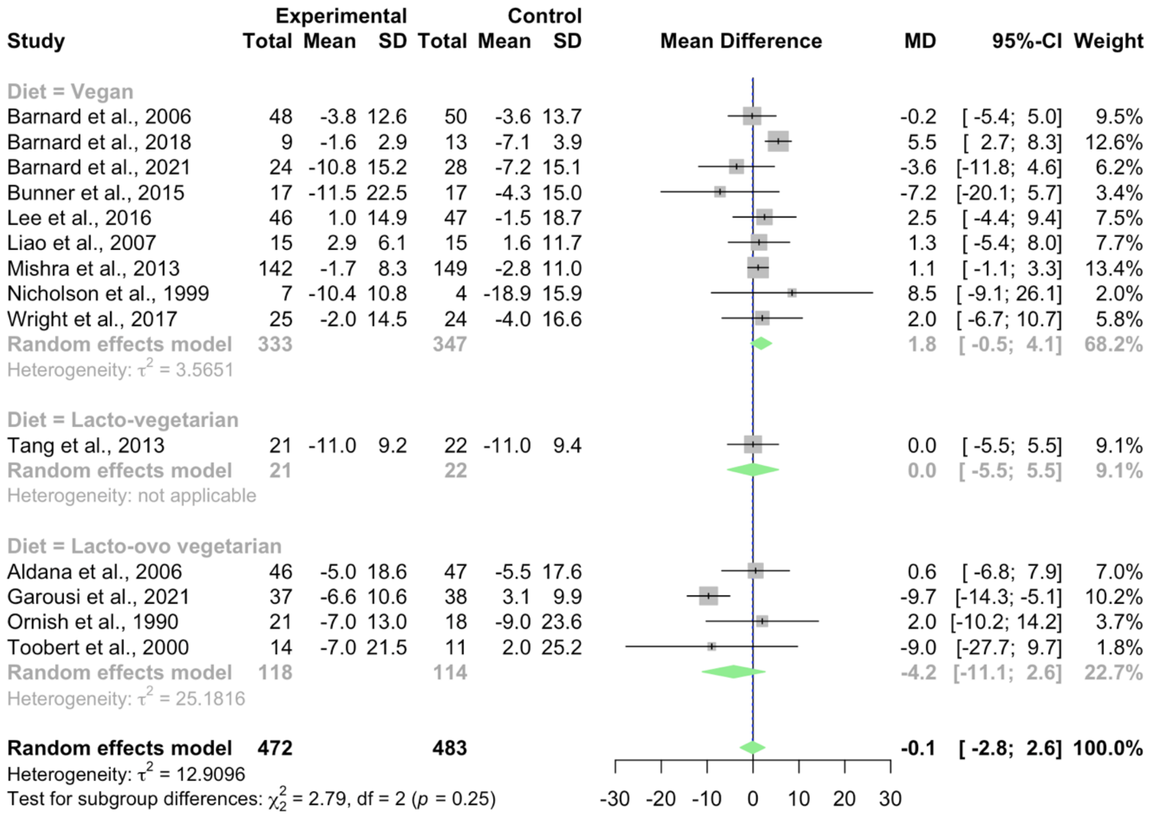

eFigure 32. Random effects model meta-analysis for changes in systolic blood pressure comparing vegetarian diets intervention and all comparison diets, grouped by vegetarian diets. CI, confidence interval; MD, mean difference; SD, standard deviation.

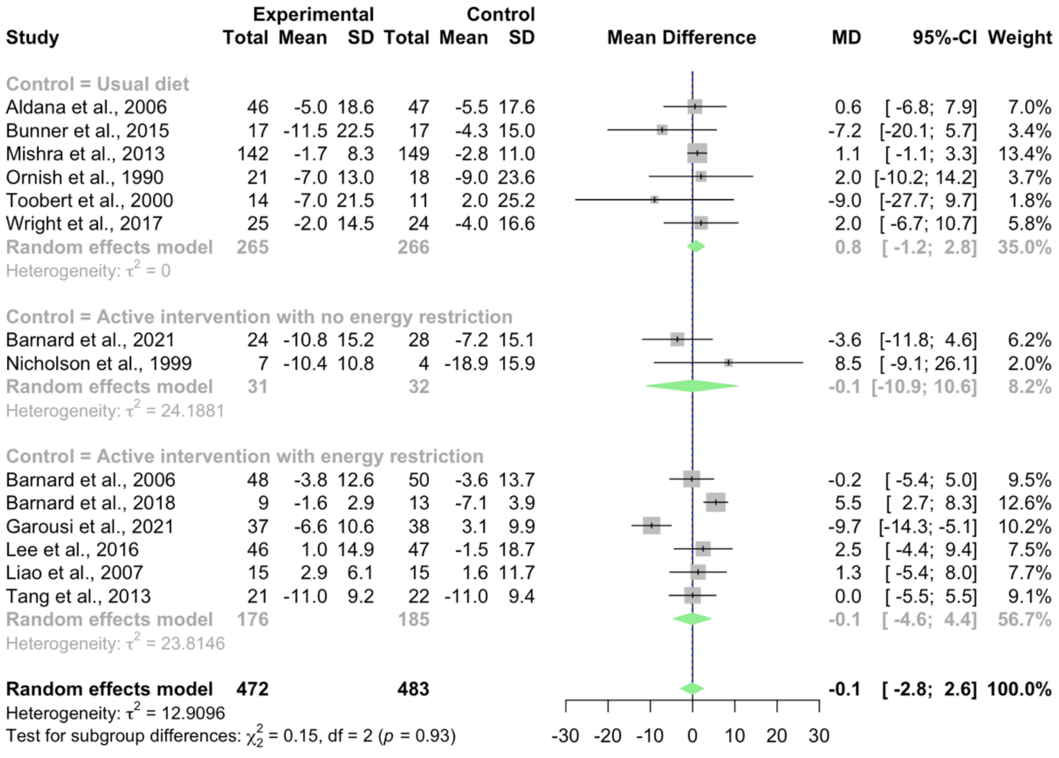

eFigure 33. Random effects model meta-analysis for changes in systolic blood pressure comparing vegetarian diets intervention and all comparison diets, grouped by control diets. CI, confidence interval; MD, mean difference; SD, standard deviation.

© 2023 Wang T et al. *JAMA Network Open.*

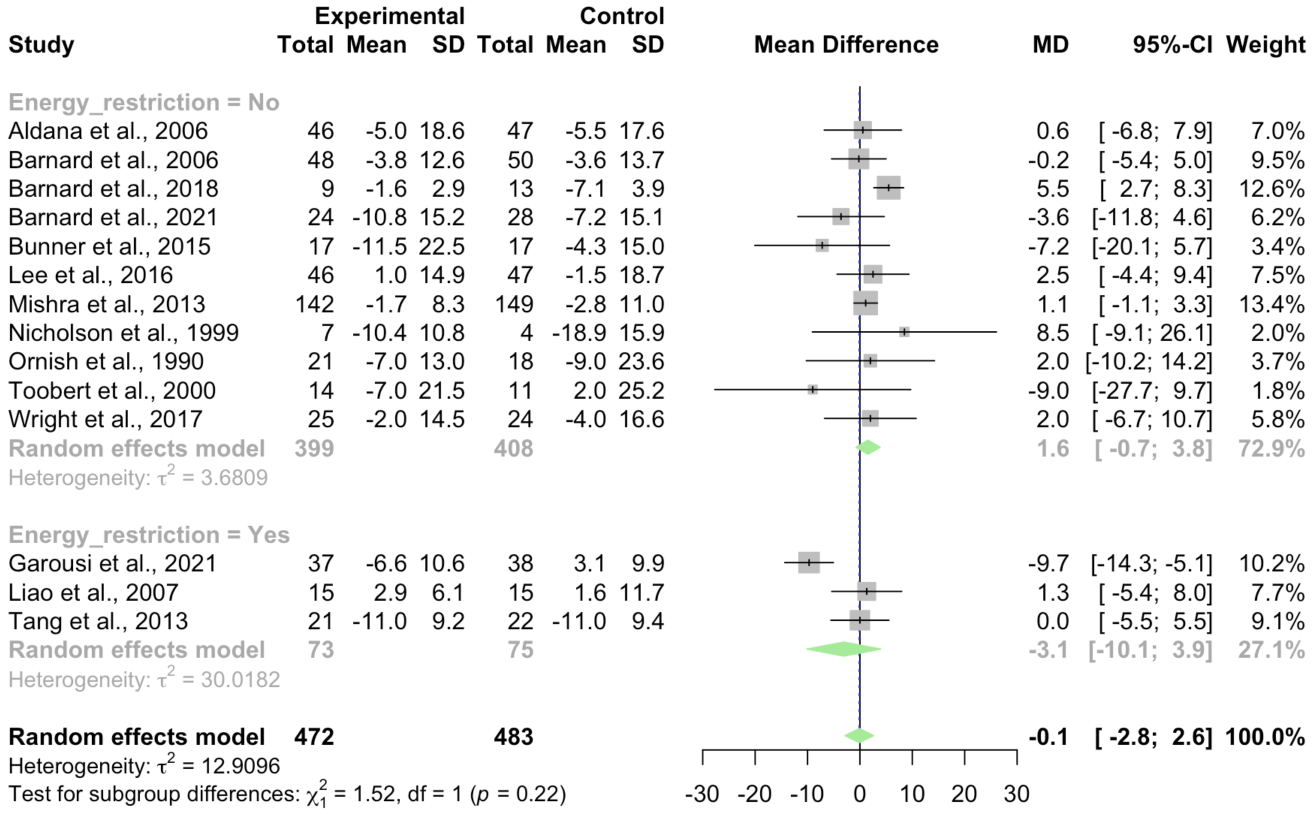

eFigure 34. Random effects model meta-analysis for changes in systolic blood pressure comparing vegetarian diets intervention and all comparison diets, grouped by energy restriction. CI, confidence interval; MD, mean difference; SD, standard deviation.

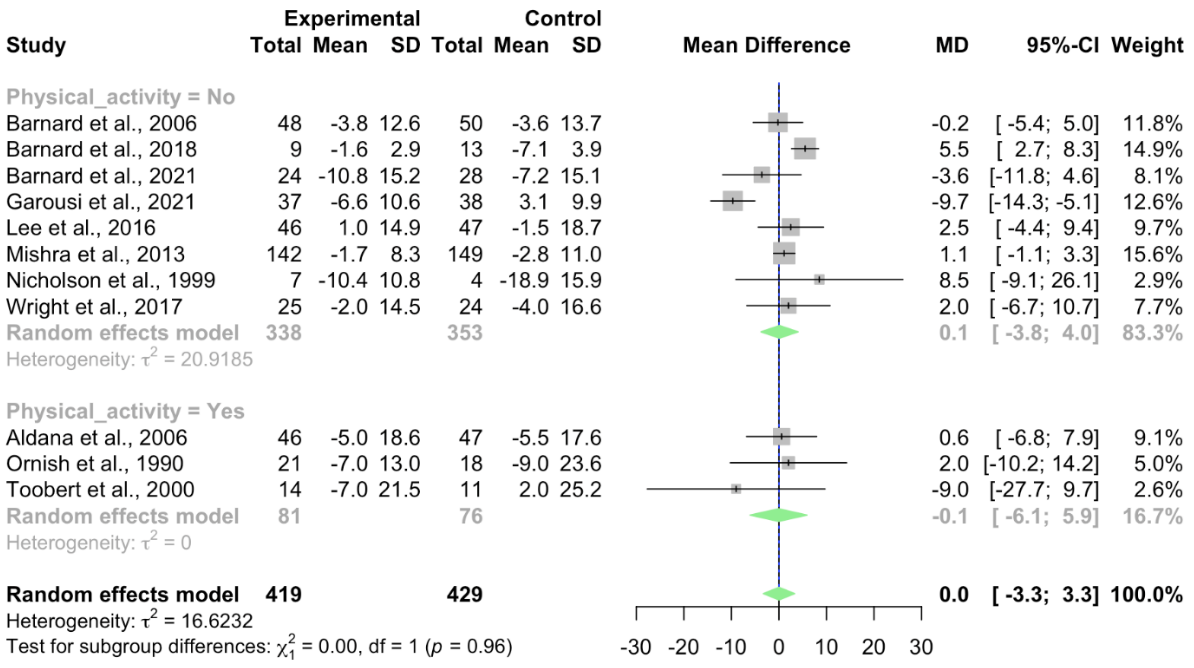

eFigure 35. Random effects model meta-analysis for changes in systolic blood pressure comparing vegetarian diets intervention and all comparison diets, grouped by physical activity. CI, confidence interval; MD, mean difference; SD, standard deviation.

© 2023 Wang T et al. *JAMA Network Open.*

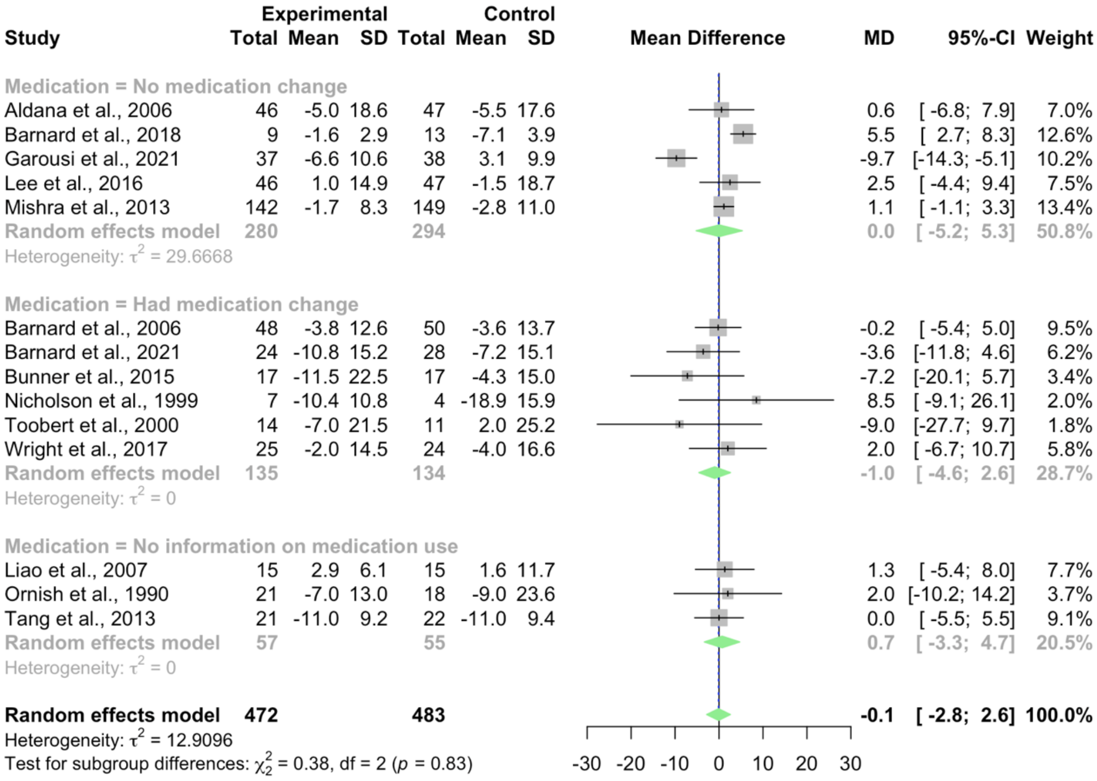

eFigure 36. Random effects model meta-analysis for changes in systolic blood pressure comparing vegetarian diets intervention and all comparison diets, grouped by medication use. CI, confidence interval; MD, mean difference; SD, standard deviation.

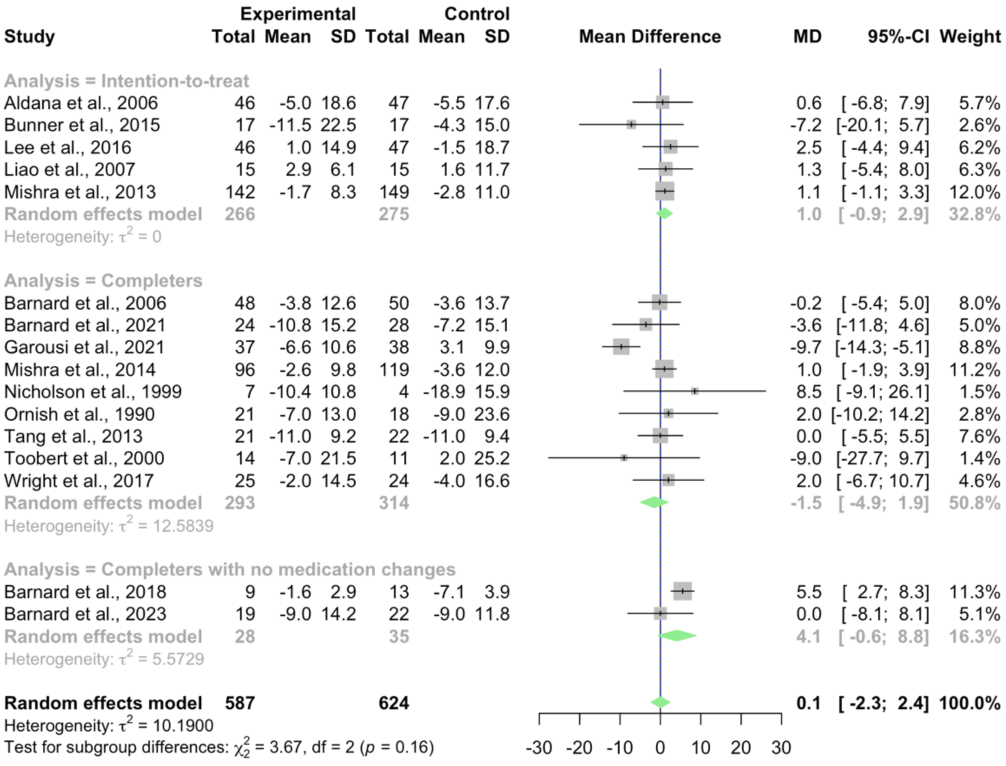

eFigure 37. Random effects model meta-analysis for changes in systolic blood pressure comparing vegetarian diets intervention and all comparison diets, grouped by analysis methods. CI, confidence interval; MD, mean difference; SD, standard deviation.

© 2023 Wang T et al. *JAMA Network Open.*

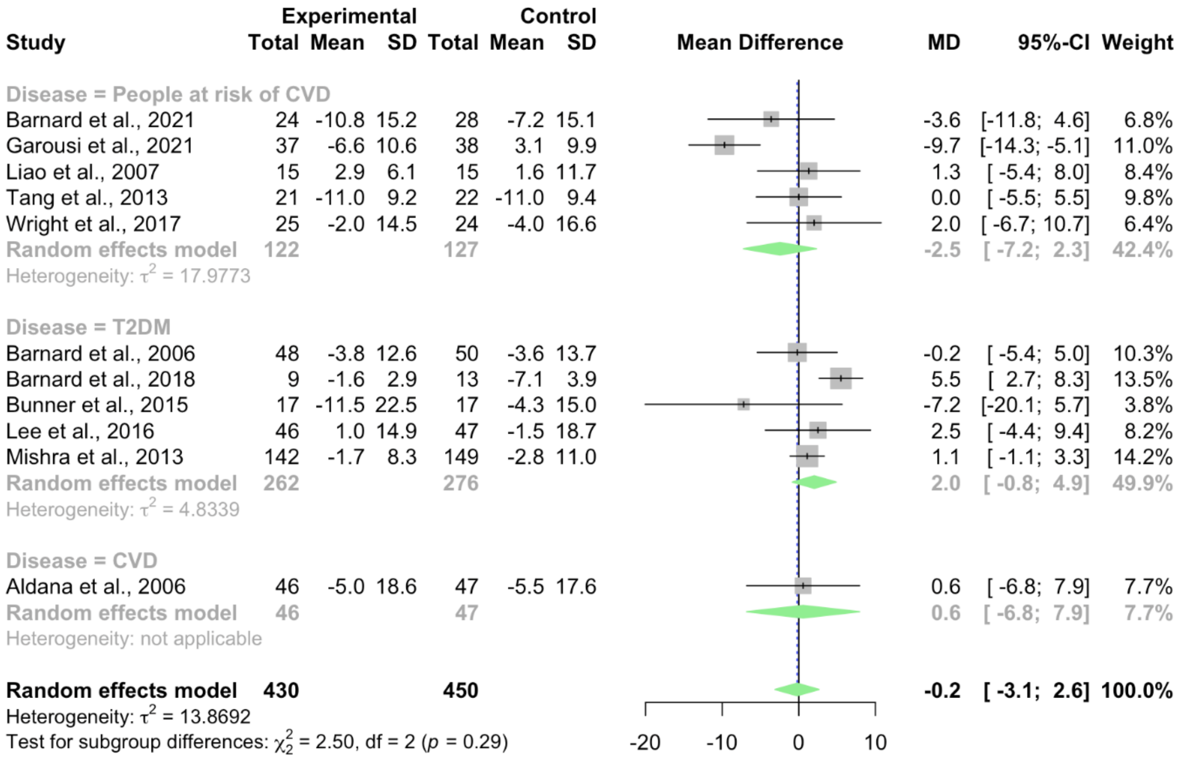

eFigure 38. Random effects model meta-analysis for changes in systolic blood pressure comparing vegetarian diets intervention and all comparison diets, grouped by disease status of participants excluding imputed data. CI, confidence interval; CVD, cardiovascular disease; MD, mean difference; SD, standard deviation; T2DM, type 2 diabetes mellitus.

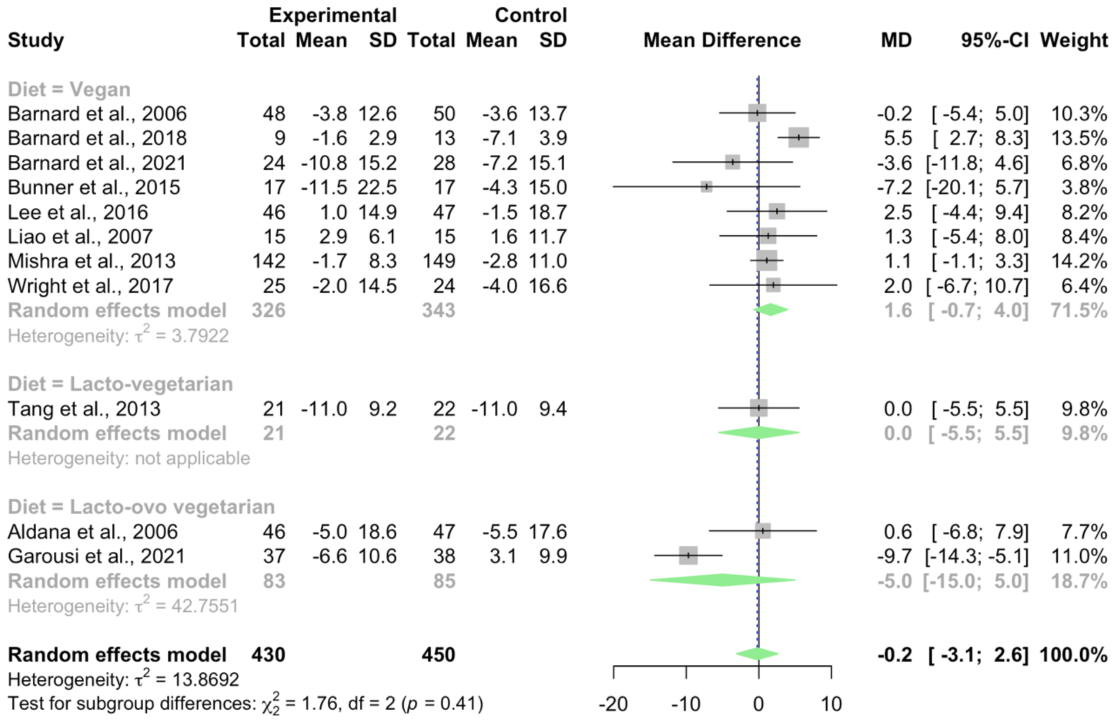

eFigure 39. Random effects model meta-analysis for changes in systolic blood pressure comparing vegetarian diets intervention and all comparison diets, grouped by various vegetarian diets excluding imputed data. CI, confidence interval; MD, mean difference; SD, standard deviation.

© 2023 Wang T et al. *JAMA Network Open.*

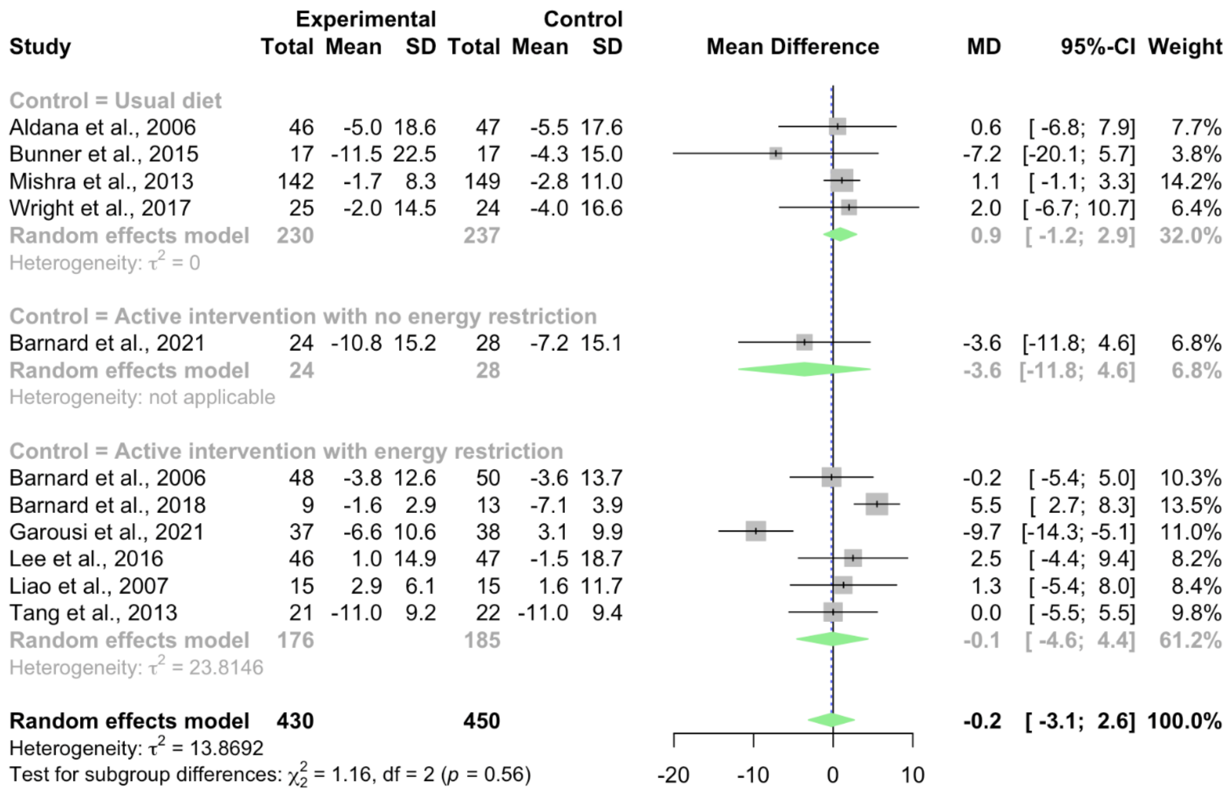

eFigure 40. Random effects model meta-analysis for changes in systolic blood pressure comparing vegetarian diets intervention and all comparison diets, grouped by control diets excluding imputed data. CI, confidence interval; MD, mean difference; SD, standard deviation.

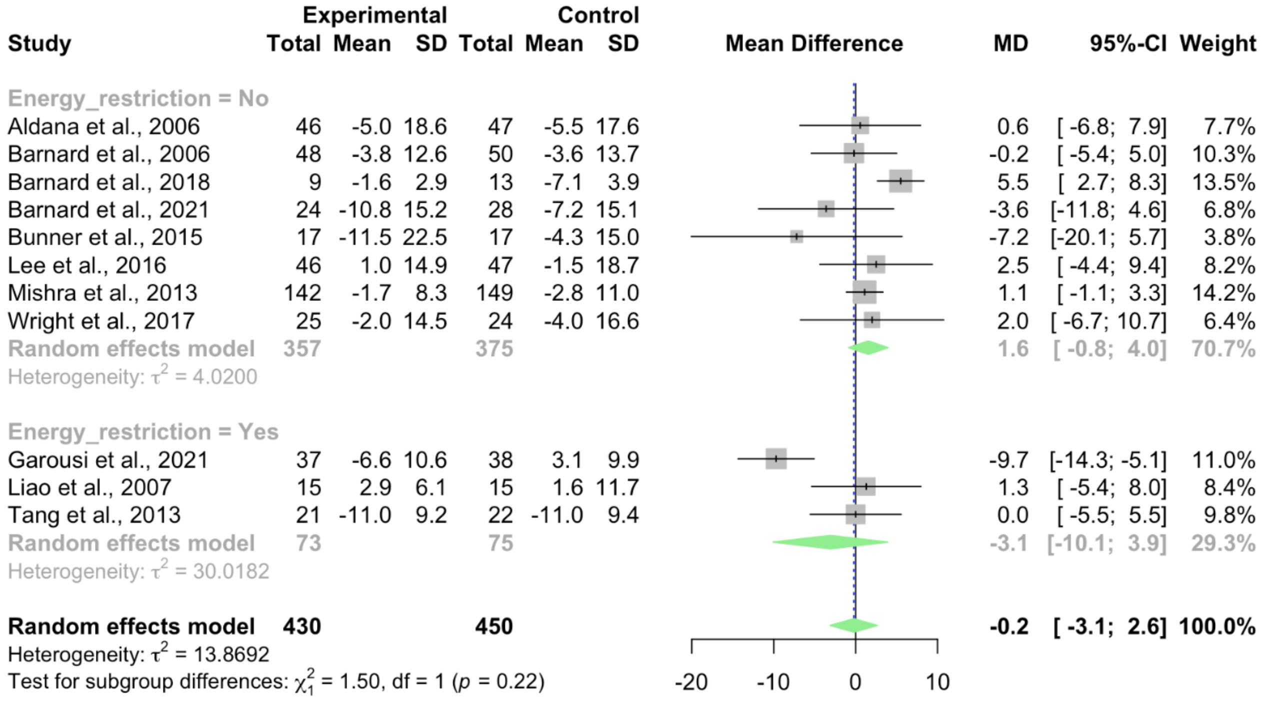

eFigure 41. Random effects model meta-analysis for changes in systolic blood pressure comparing vegetarian diets intervention and all comparison diets, grouped by energy restriction excluding imputed data. CI, confidence interval; MD, mean difference; SD, standard deviation.

© 2023 Wang T et al. *JAMA Network Open.*

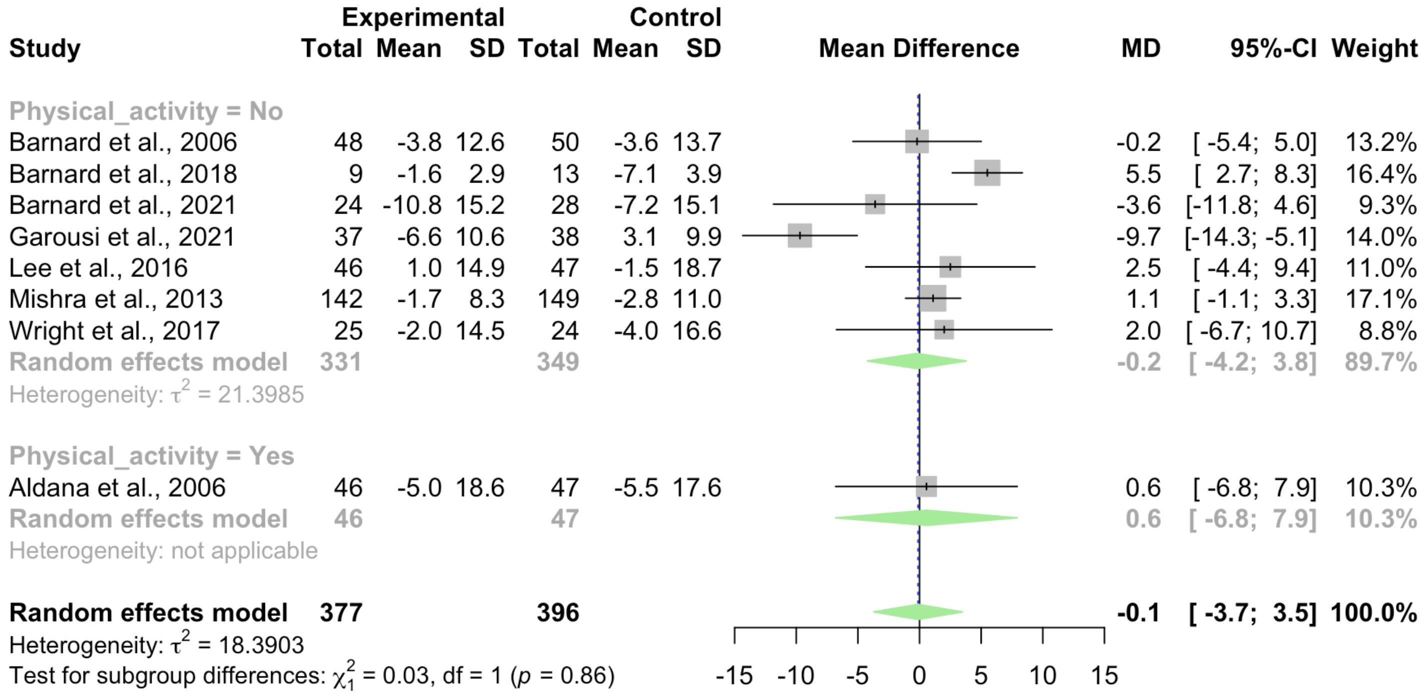

eFigure 42. Random effects model meta-analysis for changes in systolic blood pressure comparing vegetarian diets intervention and all comparison diets, grouped by physical activity excluding imputed data. CI, confidence interval; MD, mean difference; SD, standard deviation.

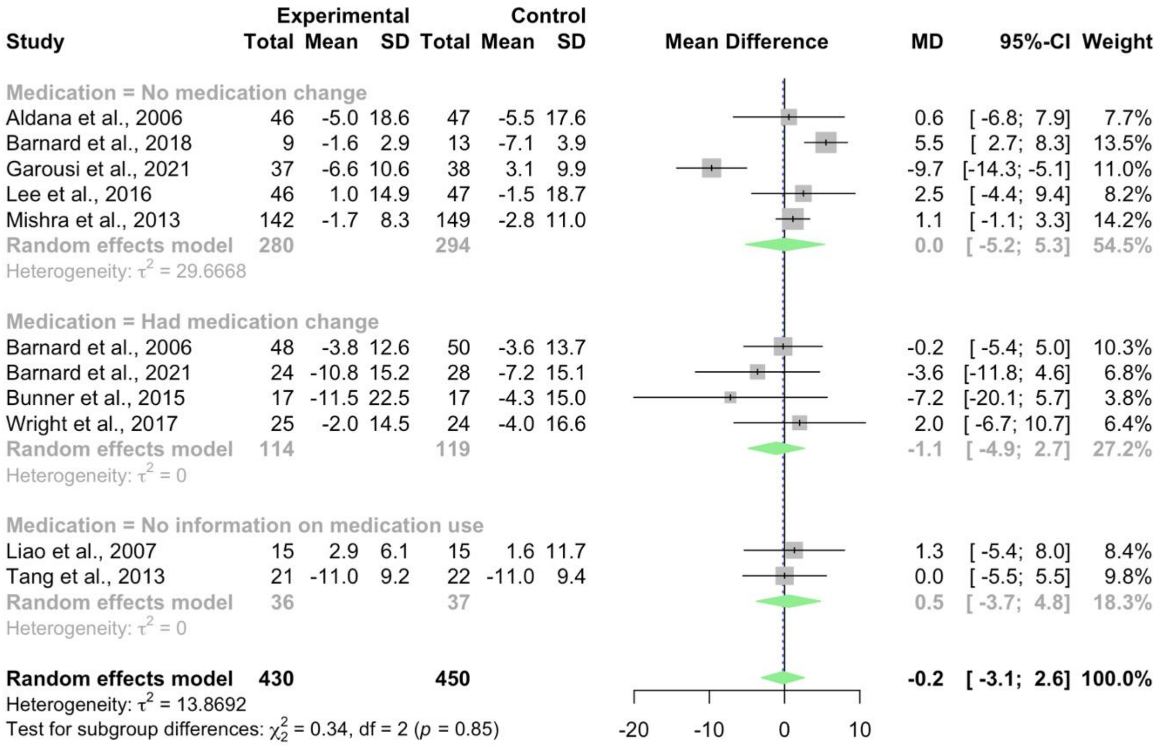

eFigure 43. Random effects model meta-analysis for changes in systolic blood pressure comparing vegetarian diets intervention and all comparison diets, grouped by medication use excluding imputed data. CI, confidence interval; MD, mean difference; SD, standard deviation.

© 2023 Wang T et al. *JAMA Network Open.*

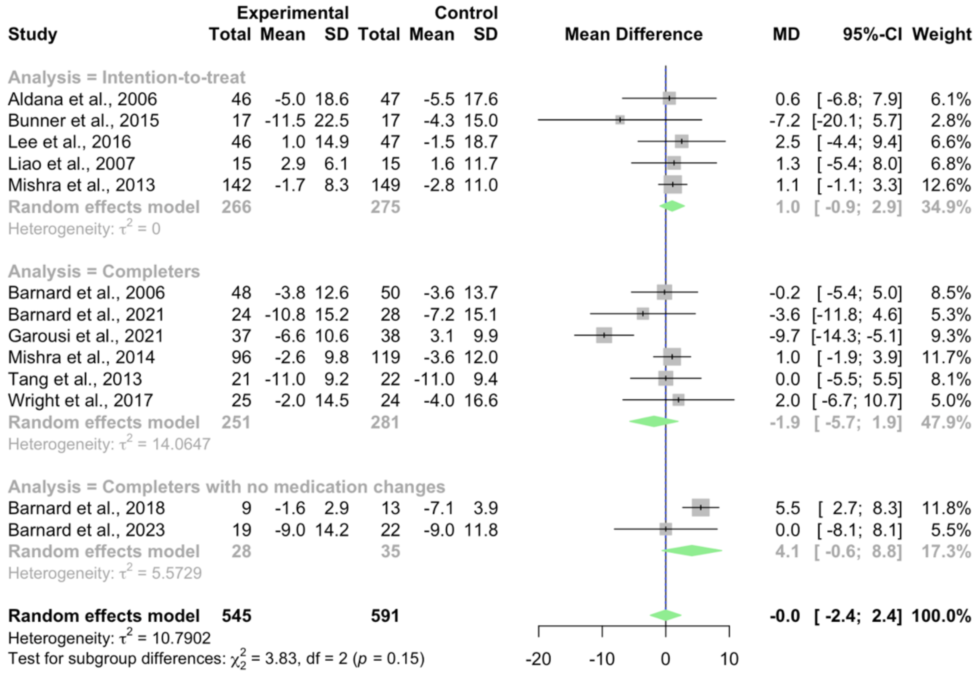

eFigure 44. Random effects model meta-analysis for changes in systolic blood pressure comparing vegetarian diets intervention and all comparison diets, grouped by analysis method excluding imputed data. CI, confidence interval; MD, mean difference; SD, standard deviation.

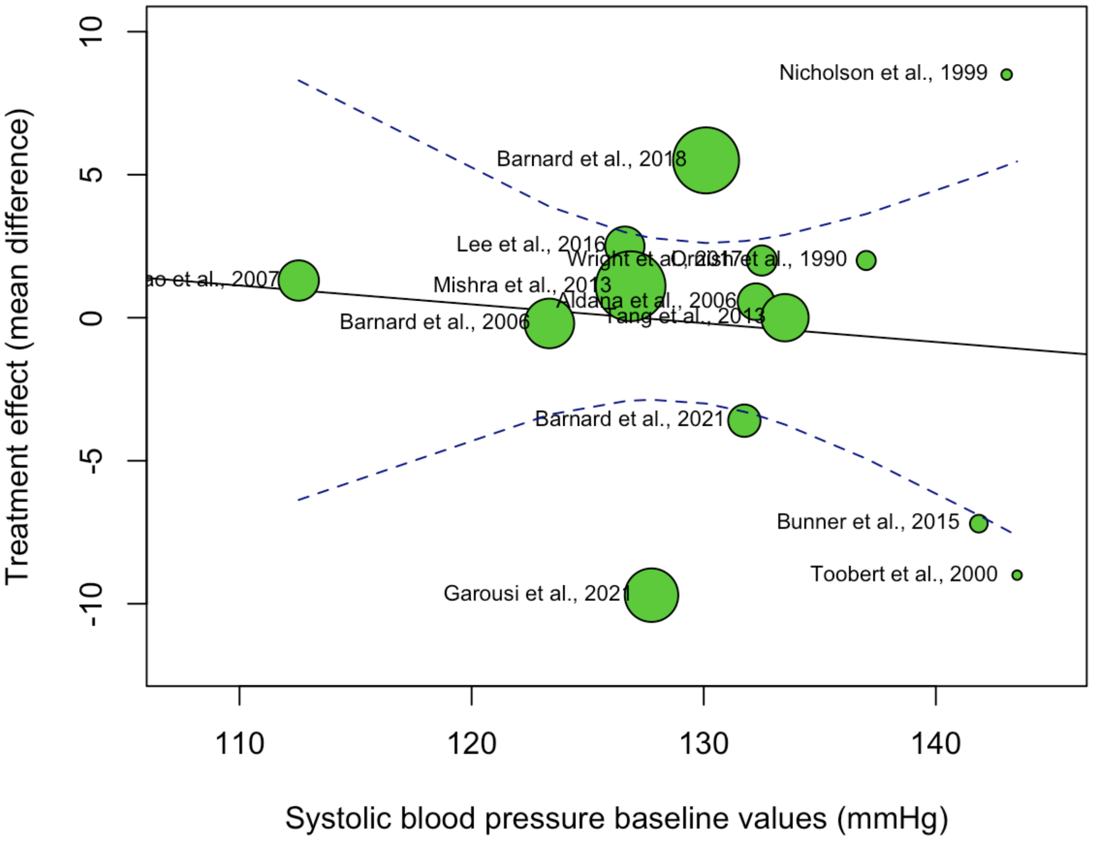

eFigure 45. Treatment effect of systolic blood pressure across increasing levels of baseline systolic blood pressure (mmHg)

© 2023 Wang T et al. *JAMA Network Open.*

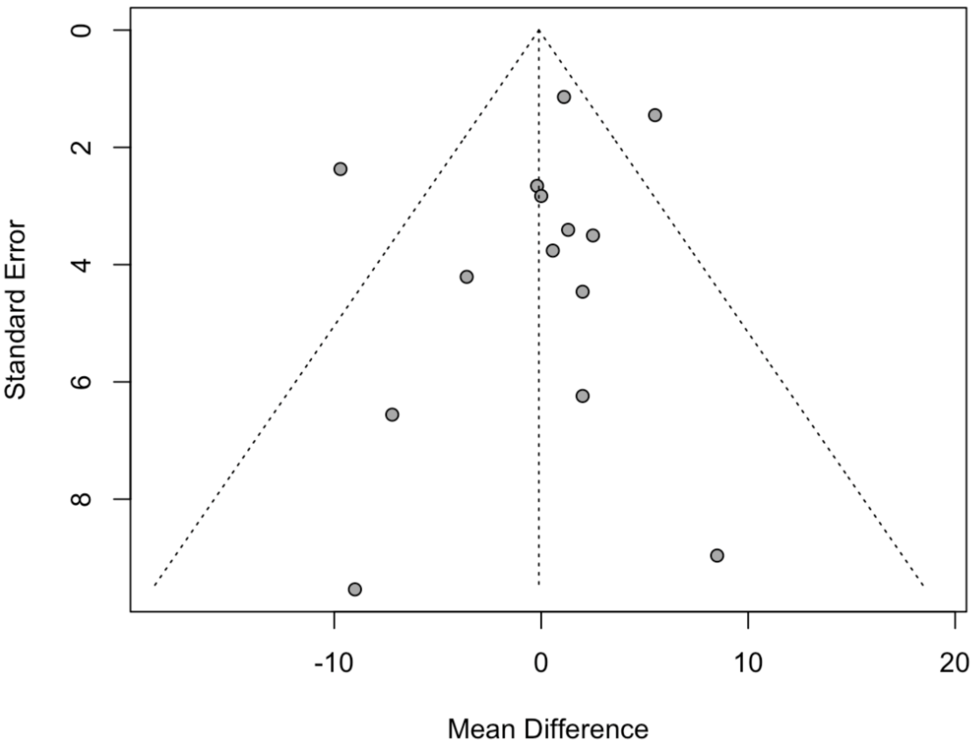

eFigure 46. Funnel plot of systolic blood pressure for risk of publication bias. Mean change in systolic blood pressure plotted against the SE of the mean change. The plot appears fairly symmetrical.

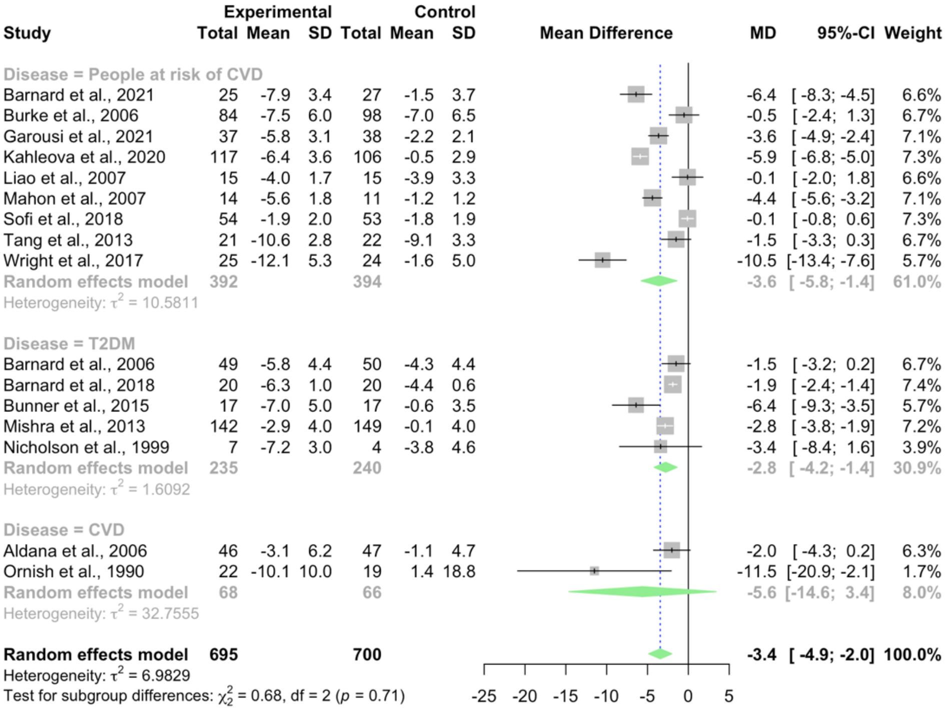

eFigure 47. Random effects model meta-analysis for changes in body weight comparing vegetarian diets intervention and all comparison diets, grouped by disease status of participants. CI, confidence interval; CVDd, cardiovascular disease; MD, mean difference; SD, standard deviation; T2DM, type 2 diabetes mellitus.

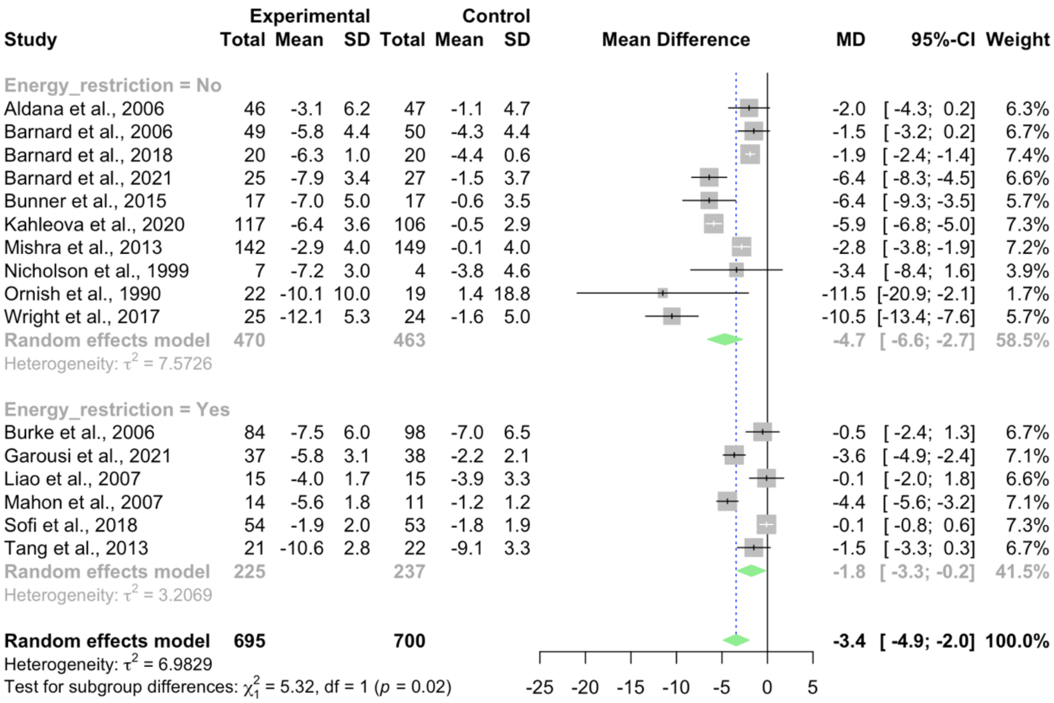

eFigure 48. Random effects model meta-analysis for changes in body weight comparing vegetarian diets intervention and all comparison diets, grouped by energy restriction. CI, confidence interval; MD, mean difference; SD, standard deviation.

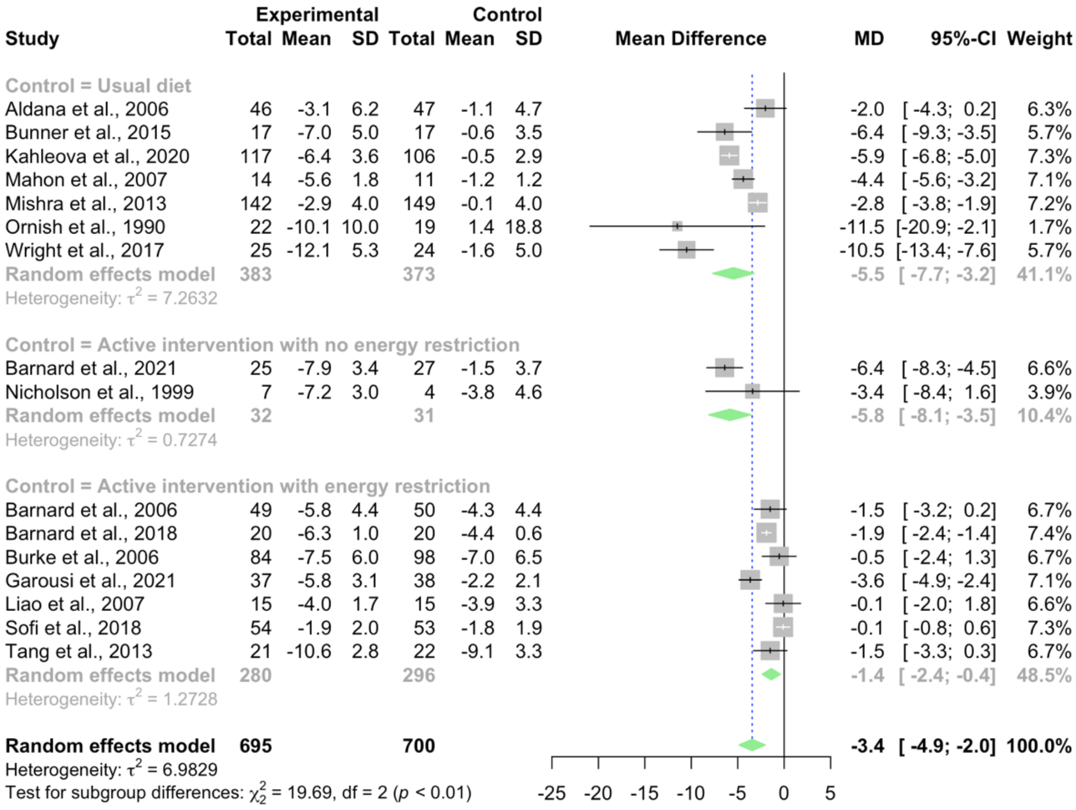

eFigure 49. Random effects model meta-analysis for changes in body weight comparing vegetarian diets intervention and all comparison diets, grouped by comparison diet. CI, confidence interval; MD, mean difference; SD, standard deviation.

© 2023 Wang T et al. *JAMA Network Open.*

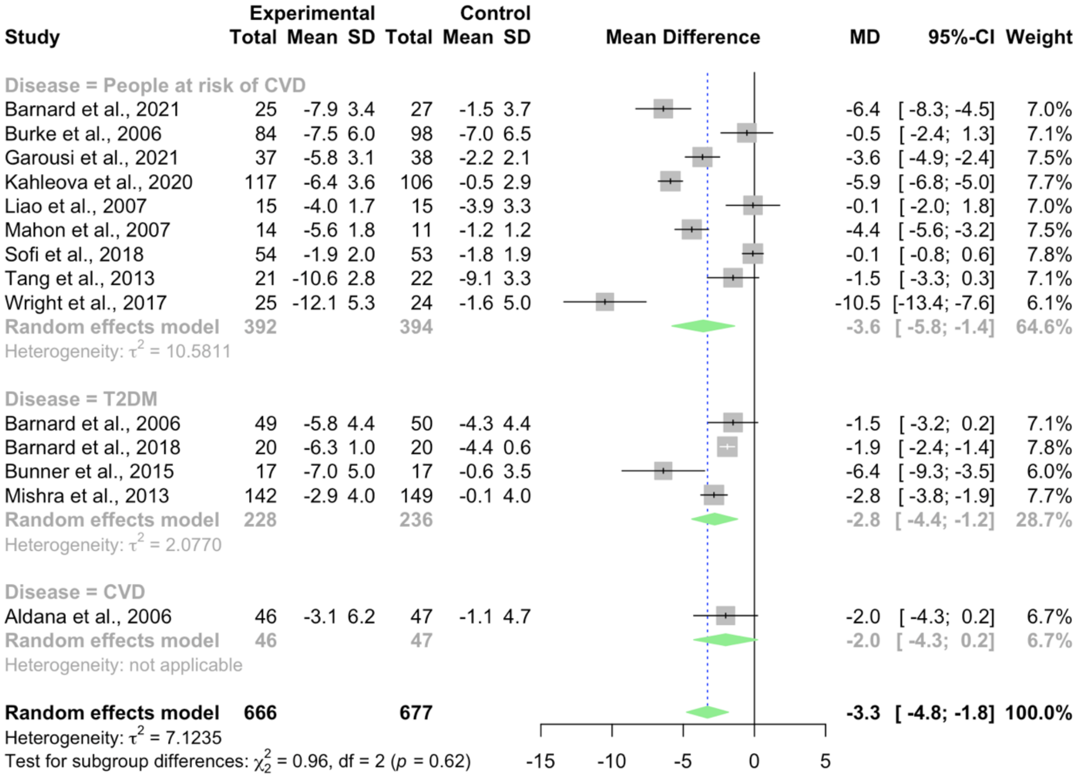

eFigure 50. Random effects model meta-analysis for changes in body weight comparing vegetarian diets intervention and all comparison diets, grouped by disease status of participants excluding imputed data. CI, confidence interval; CVD, cardiovascular disease; MD, mean difference; SD, standard deviation; T2DM, type 2 diabetes mellitus.

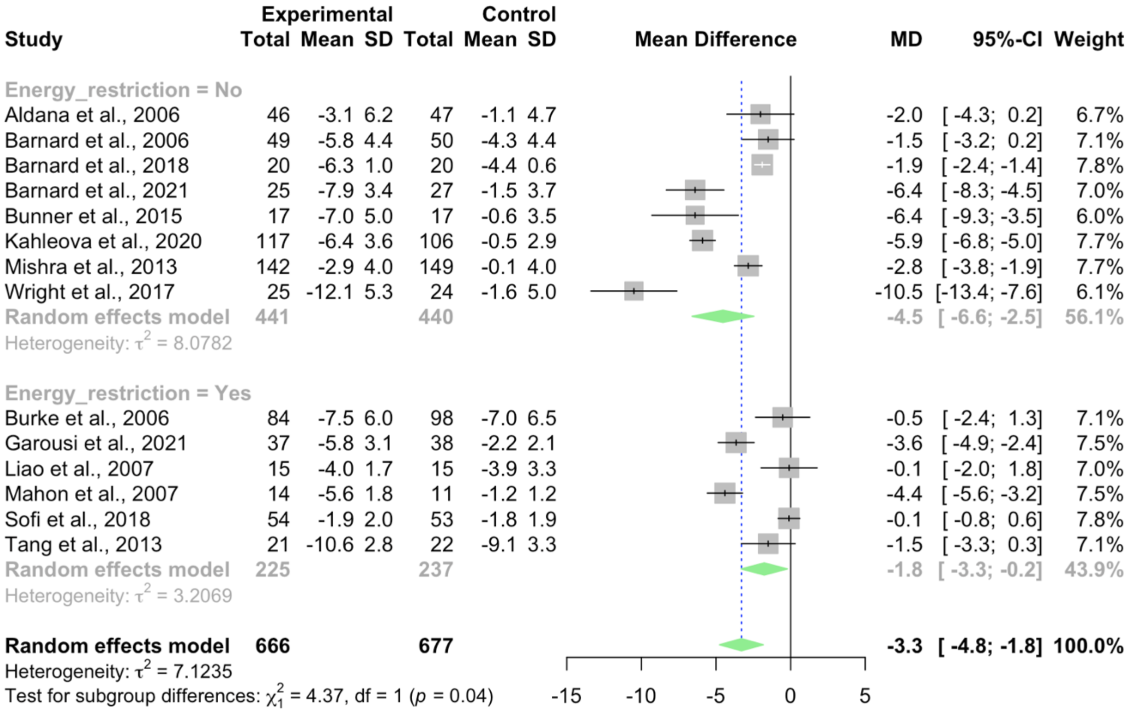

eFigure 51. Random effects model meta-analysis for changes in body weight comparing vegetarian diets intervention and all comparison diets, grouped by energy restriction excluding imputed data. CI, confidence interval; MD, mean difference; SD, standard deviation.

© 2023 Wang T et al. *JAMA Network Open.*

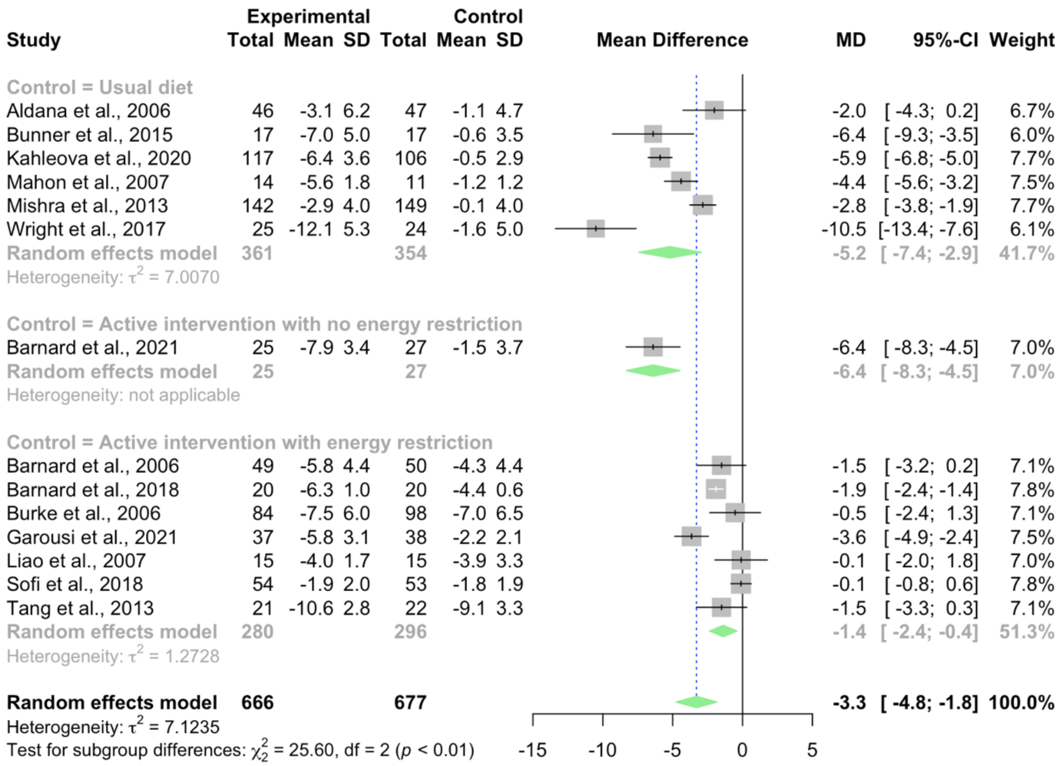

eFigure 52. Random effects model meta-analysis for changes in body weight comparing vegetarian diets intervention and all comparison diets, grouped by control diets excluding imputed data. CI, confidence interval; MD, mean difference; SD, standard deviation.

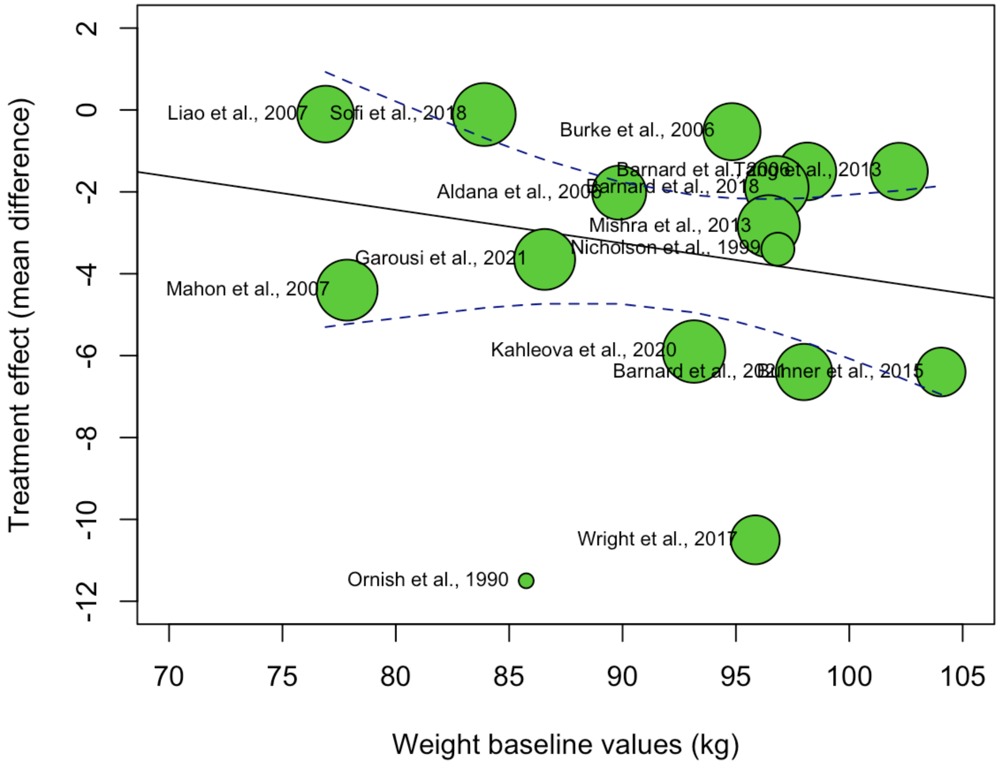

eFigure 53. Treatment effect of body weight across increasing levels of baseline body weight (kg)

© 2023 Wang T et al. *JAMA Network Open.*

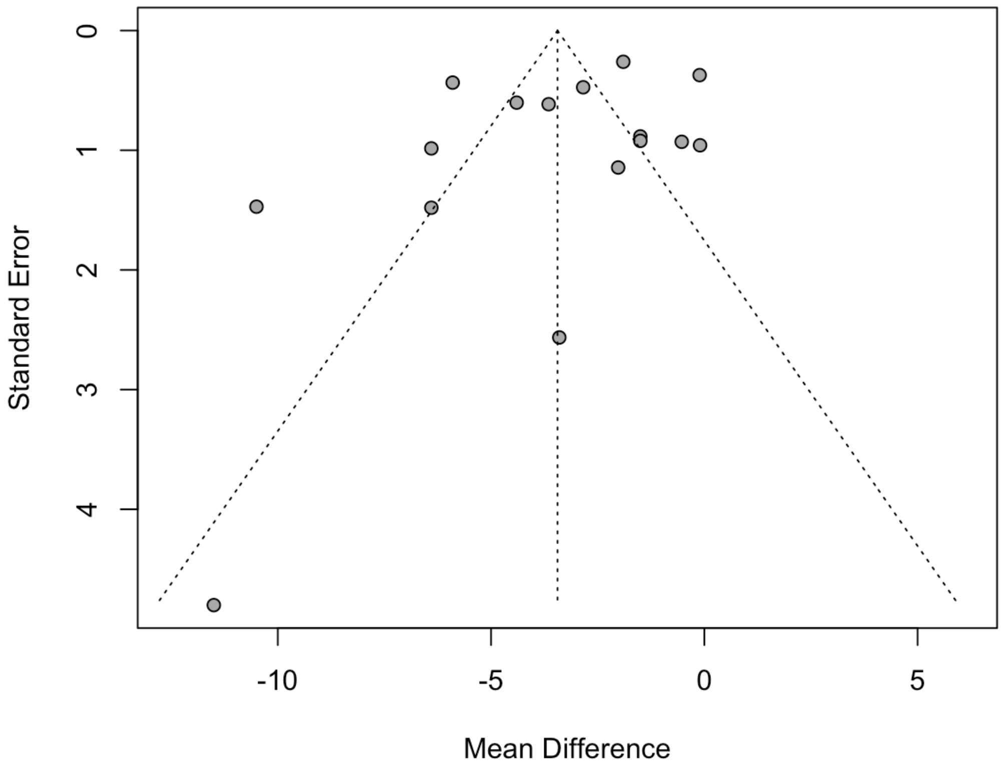

eFigure 54. Funnel plot of body weight for risk of publication bias. Mean change in body weight plotted against the SE of the mean change. The plot appears fairly symmetrical.

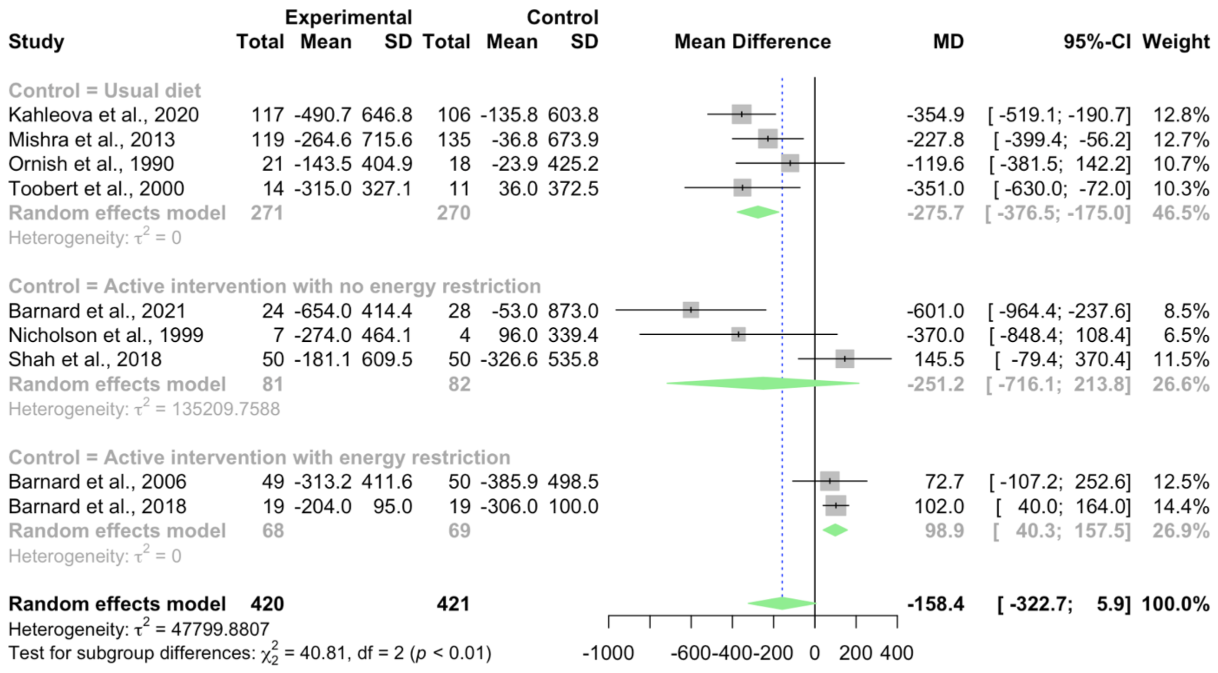

eFigure 55. Random effects model meta-analysis for changes in energy intake comparing vegetarian diets intervention without energy restriction and all comparison diets, grouped by control diets. CI, confidence interval; MD, mean difference; SD, standard deviation.

© 2023 Wang T et al. *JAMA Network Open.*

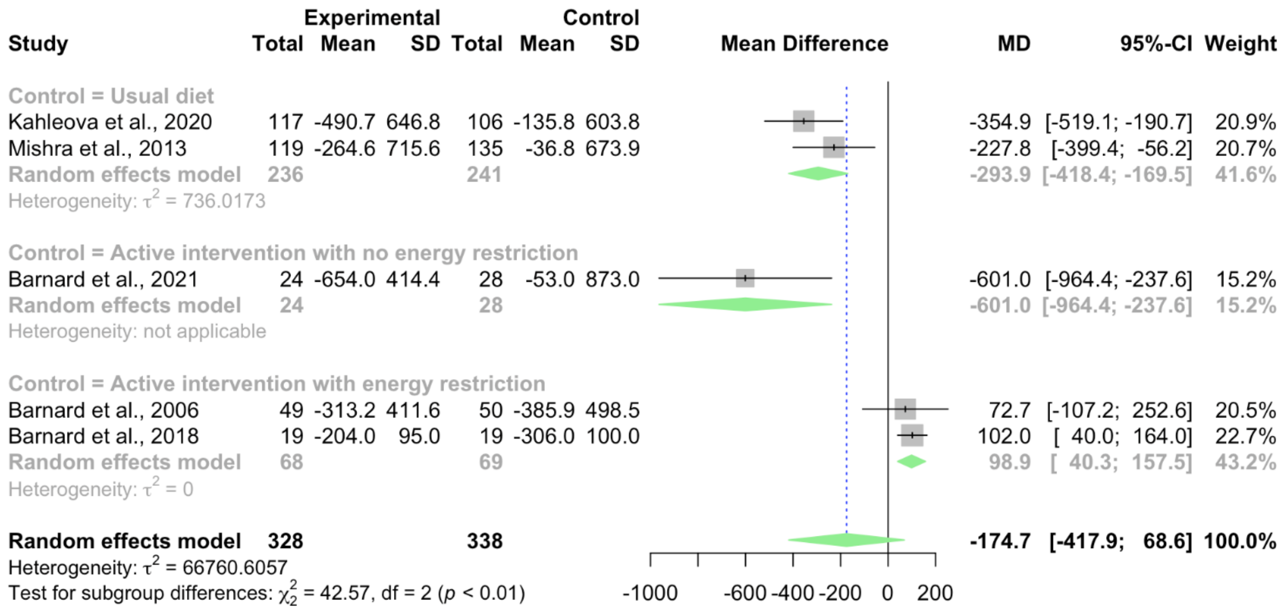

eFigure 56. Random effects model meta-analysis for changes in energy intake comparing vegetarian diets intervention without energy restriction and all comparison diets, grouped by control diets excluding imputed data. CI, confidence interval; MD, mean difference; SD, standard deviation.

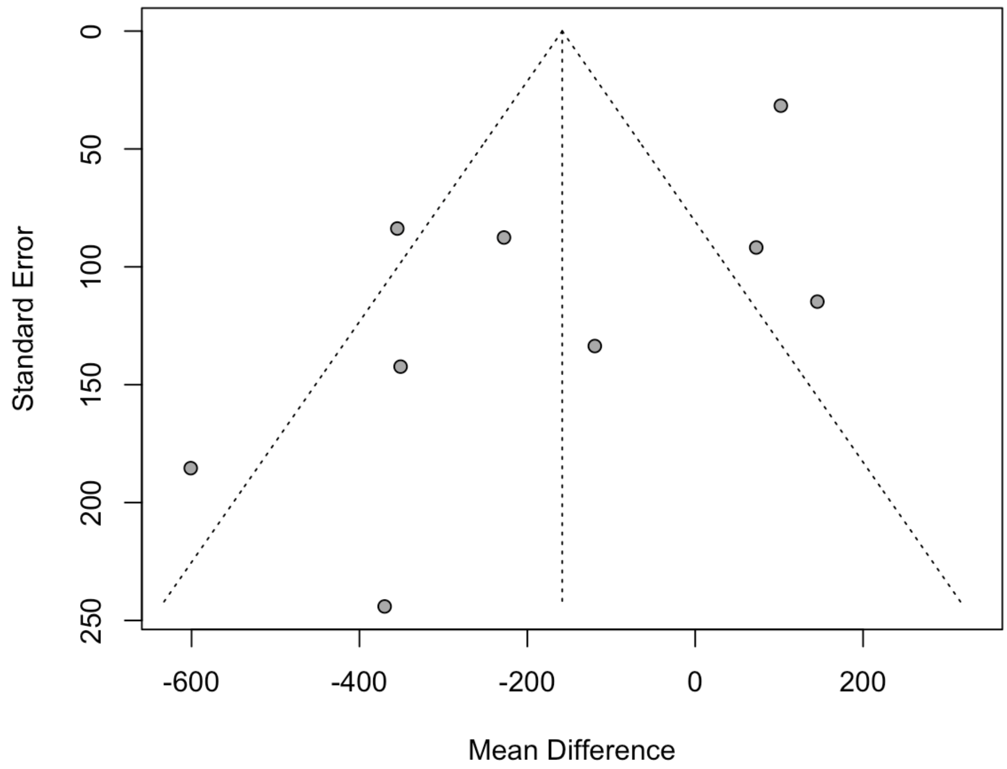

eFigure 57. Funnel plot of energy intake in studies without energy restriction for risk of publication bias. Mean change in energy intake plotted against the SE of the mean change. The plot appears fairly symmetrical.

© 2023 Wang T et al. *JAMA Network Open.*

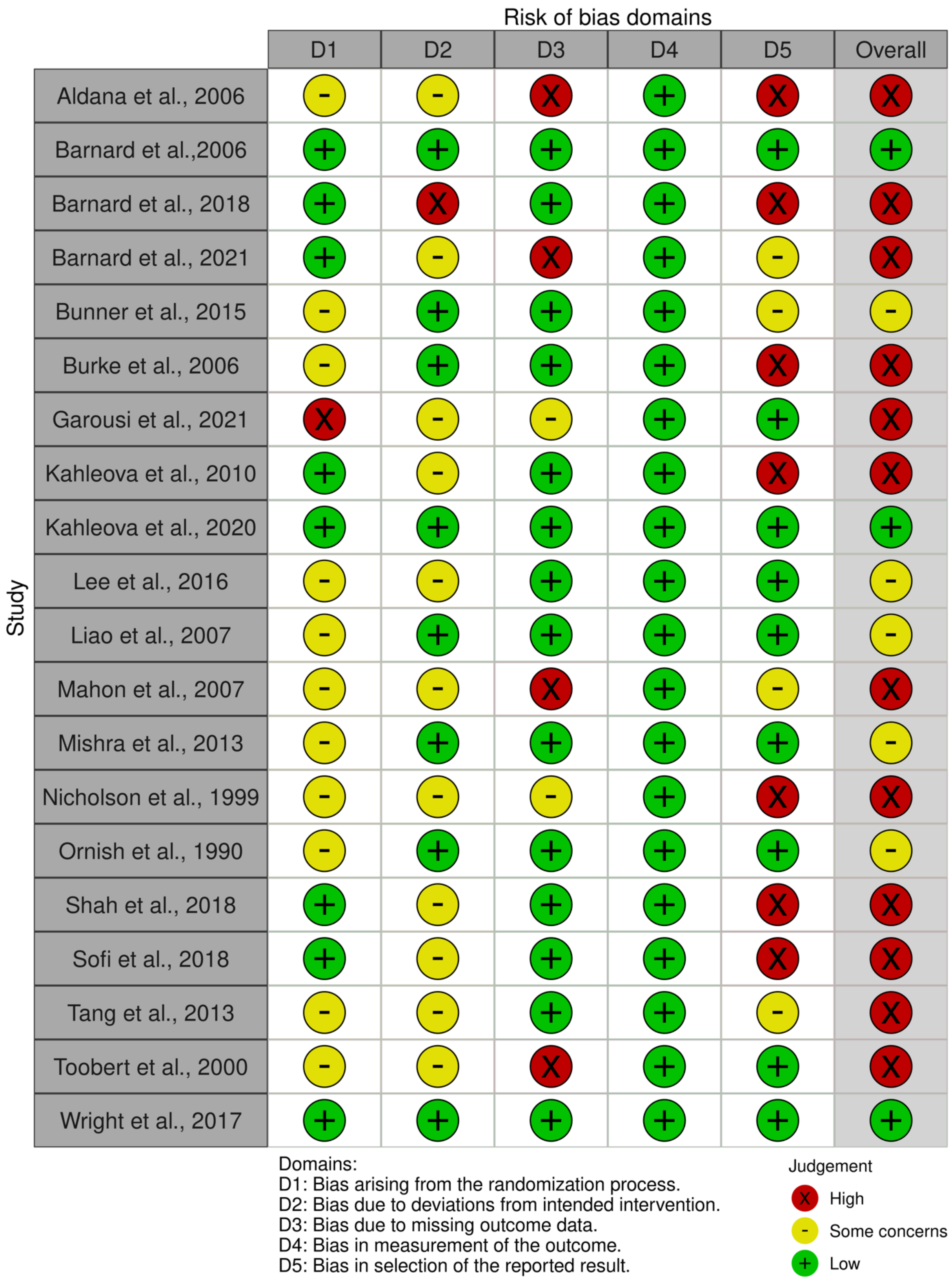

eFigure 58. Risk of Bias assessment of included randomised controlled trials
The green, yellow and red colours indicates low risk, some concerns and high risk of bias, respectively. Twelve out of the 20 included studies were assessed as overall "high risk" of bias. The randomisation process was clear in eight trials. Only eight studies had no deviations from the intended intervention. Outcome data were missing for four trials. All studies measured outcomes appropriately. Seven trials selectively reported outcome data.

© 2023 Wang T et al. *JAMA Network Open.*

References

1. Page MJ, McKenzie JE, Bossuyt PM, et al. The PRISMA 2020 statement: an updated guideline for reporting systematic reviews. *BMJ*. 2021;**372**:n71.
2. Shea BJ, Reeves BC, Wells G, et al. AMSTAR 2: a critical appraisal tool for systematic reviews that include randomised or non-randomised studies of healthcare interventions, or both. *BMJ*. 2017;**358**:j4008.
3. Higgins JPT TJ, Chandler J, Cumpston M, Li T, Page MJ, Welch VA (editors),. Cochrane Handbook for Systematic Reviews of Interventions version 6.2 (updated February 2021): Cochrane; 2021. Available from: www.training.cochrane.org/handbook.
4. Grundy SM, Stone NJ, Bailey AL, et al. 2018 AHA/ACC/AACVPR/AAPA/ABC/ACPM/ADA/AGS/APhA/ASPC/NLA/PCNA Guideline on the Management of Blood Cholesterol: Executive Summary: A Report of the American College of Cardiology/American Heart Association Task Force on Clinical Practice Guidelines. *J Am Coll Cardiol*. 2019;**73**(24):3168-209.
5. Whelton PK, Carey RM, Aronow WS, et al. 2017 ACC/AHA/AAPA/ABC/ACPM/AGS/APhA/ASH/ASPC/NMA/PCNA Guideline for the Prevention, Detection, Evaluation, and Management of High Blood Pressure in Adults: A Report of the American College of Cardiology/American Heart Association Task Force on Clinical Practice Guidelines. *Hypertension*. 2018;**71**(6):e13-e115.
6. Wan X, Wang W, Liu J, Tong T. Estimating the sample mean and standard deviation from the sample size, median, range and/or interquartile range. *BMC Medical Research Methodology*. 2014;**14**(1):135.
7. Nordestgaard BG, Langsted A, Mora S, et al. Fasting is not routinely required for determination of a lipid profile: clinical and laboratory implications including flagging at desirable concentration cut-points-a joint consensus statement from the European Atherosclerosis Society and European Federation of Clinical Chemistry and Laboratory Medicine. *Eur Heart J*. 2016;**37**(25):1944-58.
8. Wing RR, Lang W, Wadden TA, et al. Benefits of modest weight loss in improving cardiovascular risk factors in overweight and obese individuals with type 2 diabetes. *Diabetes Care*. 2011;**34**(7):1481-6.
9. Balduzzi S, Rücker G, Schwarzer G. How to perform a meta-analysis with R: a practical tutorial. *Evid Based Ment Health*. 2019;**22**(4):153-60.
10. DerSimonian R, Laird N. Meta-analysis in clinical trials. *Control Clin Trials*. 1986;**7**(3):177-88.
11. Kulinskaya E, Hoaglin DC, Bakbergenuly I, Newman J. A Q statistic with constant weights for assessing heterogeneity in meta-analysis. *Res Synth Methods*. 2021;**12**(6):711-30.
12. Sterne JAC SJ, Page MJ, Elbers RG, Blencowe NS, Boutron I, Cates CJ, Cheng H-Y, Corbett MS, Eldridge SM, Hernán MA, Hopewell S, Hróbjartsson A, Junqueira DR, Jüni P, Kirkham JJ, Lasserson T, Li T, McAleenan A, Reeves BC, Shepperd S, Shrier I, Stewart LA, Tilling K, White IR, Whiting PF, Higgins JPT. RoB 2: a revised tool for assessing risk of bias in randomised trials. *BMJ*. 2019;**366**(l4898).
13. Guyatt G, Oxman AD, Akl EA, et al. GRADE guidelines: 1. Introduction—GRADE evidence profiles and summary of findings tables. *Journal of Clinical Epidemiology*. 2011;**64**(4):383-94.
14. GRADEpro GDT. GRADEpro Guideline Development Tool [Software]: McMaster University and Evidence Prime; 2021 [cited 2021 15th Dec]. Available from: gradepro.org.
15. Guyatt GH, Oxman AD, Kunz R, et al. GRADE guidelines: 2. Framing the question and deciding on important outcomes. *Journal of Clinical Epidemiology*. 2011;**64**(4):395-400.
16. Hajar R. Risk Factors for Coronary Artery Disease: Historical Perspectives. *Heart views : the official journal of the Gulf Heart Association*. 2017;**18**(3):109-14.
17. Nelson RH. Hyperlipidemia as a risk factor for cardiovascular disease. *Primary care*. 2013;**40**(1):195-211.
18. Cavero-Redondo I, Peleteiro B, Álvarez-Bueno C, Rodriguez-Artalejo F, Martínez-Vizcaíno V. Glycated haemoglobin A1c as a risk factor of cardiovascular outcomes and all-cause mortality in diabetic and non-diabetic populations: a systematic review and meta-analysis. *BMJ Open*. 2017;**7**(7):e015949.
19. Tune JD, Goodwill AG, Sassoon DJ, Mather KJ. Cardiovascular consequences of metabolic syndrome. *Translational research : the journal of laboratory and clinical medicine*. 2017;**183**:57-70.

© 2023 Wang T et al. *JAMA Network Open.*

20. Whelton PK, Carey RM, Aronow WS, et al. 2017 ACC/AHA/AAPA/ABC/ACPM/AGS/APhA/ASH/ASPC/NMA/PCNA Guideline for the Prevention, Detection, Evaluation, and Management of High Blood Pressure in Adults: A Report of the American College of Cardiology/American Heart Association Task Force on Clinical Practice Guidelines. *J Am Coll Cardiol*. 2018;**71**(19):e127-e248.
21. National Institutes of Health. High Blood Cholesterol. National Cholesterol Education Program [Internet]. 2005. Available from: https://www.nhlbi.nih.gov/files/docs/public/heart/wyntk.pdf.
22. American Diabetes Association. Diagnosis and Classification of Diabetes Mellitus. *Diabetes Care*. 2010;**33**(Supplement 1):S62-S9.
23. Huang PL. A comprehensive definition for metabolic syndrome. *Disease models & mechanisms*. 2009;**2**(5-6):231-7.
24. Aldana SG, Greenlaw R, Salberg A, Merrill RM, Hager R, Jorgensen RB. The Effects of an Intensive Lifestyle Modification Program on Carotid Artery Intima-Media Thickness: A Randomized Trial. *American Journal of Health Promotion*. 2007;**21**(6):510-6.
25. Barnard ND, Cohen J, Jenkins DJ, et al. A low-fat vegan diet improves glycemic control and cardiovascular risk factors in a randomized clinical trial in individuals with type 2 diabetes. *Diabetes Care*. 2006;**29**(8):1777-83.
26. Barnard ND, Cohen J, Jenkins DJ, et al. A low-fat vegan diet and a conventional diabetes diet in the treatment of type 2 diabetes: a randomized, controlled, 74-wk clinical trial. *American journal of clinical nutrition*. 2009;**89**(5):1588S-96S.
27. Barnard ND, Gloede L, Cohen J, et al. A low-fat vegan diet elicits greater macronutrient changes, but is comparable in adherence and acceptability, compared with a more conventional diabetes diet among individuals with type 2 diabetes. *Journal of the American Dietetic Association*. 2009;**109**(2):263-72.
28. Turner-McGrievy GM, Barnard ND, Cohen J, Jenkins DJ, Gloede L, Green AA. Changes in nutrient intake and dietary quality among participants with type 2 diabetes following a low-fat vegan diet or a conventional diabetes diet for 22 weeks. *Journal of the American Dietetic Association*. 2008;**108**(10):1636-45.
29. Barnard ND, Levin SM, Gloede L, Flores R. Turning the Waiting Room into a Classroom: Weekly Classes Using a Vegan or a Portion-Controlled Eating Plan Improve Diabetes Control in a Randomized Translational Study. *Journal of the Academy of Nutrition & Dietetics*. 2018;**118**(6):1072-9.
30. Barnard ND, Alwarith J, Rembert E, et al. A Mediterranean Diet and Low-Fat Vegan Diet to Improve Body Weight and Cardiometabolic Risk Factors: A Randomized, Cross-over Trial. *Journal of the American College of Nutrition*. 2021:1-13.
31. Bunner AE, Wells CL, Gonzales J, Agarwal U, Bayat E, Barnard ND. A dietary intervention for chronic diabetic neuropathy pain: a randomized controlled pilot study. *Nutrition & Diabetes*. 2015;**5**:e158.
32. Burke LE, Styn MA, Steenkiste AR, Music E, Warziski M, Choo J. A randomized clinical trial testing treatment preference and two dietary options in behavioral weight management: preliminary results of the impact of diet at 6 months--PREFER study. *Obesity*. 2006;**14**(11):2007-17.
33. Burke LE, Choo J, Music E, et al. PREFER study: A randomized clinical trial testing treatment preference and two dietary options in behavioral weight management - Rationale, design and baseline characteristics. *Contemporary Clinical Trials*. 2006;**27**(1):34-48.
34. Burke LE, Warziski M, Styn MA, Music E, Hudson AG, Sereika SM. A randomized clinical trial of a standard versus vegetarian diet for weight loss: the impact of treatment preference. *International Journal of Obesity*. 2008;**32**(1):166-76.
35. Garousi N, Tamizifar B, Pourmasoumi M, et al. Effects of lacto-ovo-vegetarian diet vs. standard-weight-loss diet on obese and overweight adults with non-alcoholic fatty liver disease: a randomised clinical trial. *Archives of physiology and biochemistry*. 2021:1-9.
36. Kahleova H, Matoulek M, Malinska H, et al. Vegetarian diet improves insulin resistance and oxidative stress markers more than conventional diet in subjects with Type 2 diabetes. *Diabetic Medicine*. 2011;**28**(5):549-59.

© 2023 Wang T et al. *JAMA Network Open.*

37. Kahleova H, Petersen KF, Shulman GI, et al. Effect of a Low-Fat Vegan Diet on Body Weight, Insulin Sensitivity, Postprandial Metabolism, and Intramyocellular and Hepatocellular Lipid Levels in Overweight Adults: A Randomized Clinical Trial. *JAMA Network Open*. 2020;**3**(11):e2025454.
38. Lee YM, Kim SA, Lee IK, et al. Effect of a Brown Rice Based Vegan Diet and Conventional Diabetic Diet on Glycemic Control of Patients with Type 2 Diabetes: A 12-Week Randomized Clinical Trial. *PLoS ONE [Electronic Resource]*. 2016;**11**(6):e0155918.
39. Liao F-H, Shieh M-J, Yang S-C, Lin S-H, Chien Y-W. Effectiveness of a soy-based compared with a traditional low-calorie diet on weight loss and lipid levels in overweight adults. *Nutrition*. 2007;**23**(7):551-6.
40. Mahon AK, Flynn MG, Stewart LK, et al. Protein intake during energy restriction: effects on body composition and markers of metabolic and cardiovascular health in postmenopausal women. *Journal of the American College of Nutrition*. 2007;**26**(2):182-9.
41. Mishra S, Xu J, Agarwal U, Gonzales J, Levin S, Barnard ND. A multicenter randomized controlled trial of a plant-based nutrition program to reduce body weight and cardiovascular risk in the corporate setting: the GEICO study. *European Journal of Clinical Nutrition*. 2013;**67**(7):718-24.
42. Mishra S BNDGJXJAULS. Nutrient intake in the GEICO multicenter trial: the effects of a multicomponent worksite intervention. *European journal of clinical nutrition*. 2013;**67**(10):1066.
43. Nicholson AS, Sklar M, Barnard ND, Gore S, Sullivan R, Browning S. Toward improved management of NIDDM: A randomized, controlled, pilot intervention using a lowfat, vegetarian diet. *Preventive Medicine*. 1999;**29**(2):87-91.
44. Ornish D, Brown SE, Scherwitz LW, et al. Can lifestyle changes reverse coronary heart disease? The Lifestyle Heart Trial. *Lancet*. 1990;**336**(8708):129-33.
45. Ornish D, Scherwitz LW, Billings JH, et al. Intensive lifestyle changes for reversal of coronary heart disease JAMA 1999 Apr 21;281(15): 1380. *Jama*. 1998;**280**(23):2001-7.
46. Shah B, Ganguzza L, Slater J, et al. The effect of a vegan versus AHA DiEt in coronary artery disease (EVADE CAD) trial: study design and rationale. *Contemporary clinical trials communications*. 2017;**8**:90-8.
47. Shah B, Newman JD, Woolf K, et al. Anti-Inflammatory Effects of a Vegan Diet Versus the American Heart Association-Recommended Diet in Coronary Artery Disease Trial. *Journal of the American Heart Association*. 2018;**7**(23):e011367.
48. Sofi F, Dinu M, Pagliai G, et al. Low-Calorie Vegetarian Versus Mediterranean Diets for Reducing Body Weight and Improving Cardiovascular Risk Profile: CARDIVEG Study (Cardiovascular Prevention With Vegetarian Diet). *Circulation*. 2018;**137**(11):1103-13.
49. Sofi F, Dinu M, Pagliai G, Cesari F, Marcucci R, Casini A. Mediterranean versus vegetarian diet for cardiovascular disease prevention (the CARDIVEG study): study protocol for a randomized controlled trial. *Trials [Electronic Resource]*. 2016;**17**(1):233.
50. Tang M, Armstrong CLH, Leidy HJ, Campbell WW. Normal vs. high-protein weight loss diets in men: Effects on body composition and indices of metabolic syndrome. *Obesity*. 2013;**21**(3):E204-E10.
51. Toobert DJ, Glasgow RE, Radcliffe JL. Physiologic and related behavioral outcomes from the Women's Lifestyle Heart Trial. *Annals of Behavioral Medicine*. 2000;**22**(1):1-9.
52. Wright N, Wilson L, Smith M, Duncan B, McHugh P. The BROAD study: A randomised controlled trial using a whole food plant-based diet in the community for obesity, ischaemic heart disease or diabetes. *Nutrition and Diabetes*. 2017;**7**(3).
53. Cassidy S. A randomised controlled trial to evaluate an intensive lifestyle program for reversal of coronary heart disease 2020 [updated 9th Nov 2020; cited 2021 22nd Sep]. Available from: https://trialsearch.who.int/?TrialID=ACTRN12620001151921.
54. Kahleova H. Effect of a Dietary Intervention on Intracellular Lipid, Insulin Sensitivity, and Glycemic Control in Type 2 Diabetes. *https://clinicaltrialsgov/show/NCT04088981*. 2019.
55. Michalsen A. Plant-based Nutrition for Patients With Cardiovascular Risk Factors (CardioVeg) 2019 [updated 12th Feb, 2021; cited 2021 23rd Sep]. Available from: https://clinicaltrials.gov/ct2/show/NCT03901183.
56. Turner-McGrievy B. Nutritious Eating With Soul (The NEW Soul Study). *https://clinicaltrialsgov/show/NCT03354377*. 2017.
© 2023 Wang T et al. *JAMA Network Open.*

57. Wright N. The EDGe (End Diabetes Gisborne) trial. Using the whole-foods, plant-based diet in a community programme for people with obesity and diabetes. http://wwwwhoint/trialsearch/Trial2aspx?TrialID=ACTRN12617000541303. 2017.
58. Acharya SD, Elci OU, Sereika SM, et al. Adherence to a behavioral weight loss treatment program enhances weight loss and improvements in biomarkers. *Patient preference & adherence*. 2009;**3**:151-60.
59. Acharya SD, Brooks MM, Evans RW, Linkov F, Burke LE. Weight loss is more important than the diet type in improving adiponectin levels among overweight/obese adults. *Journal of the American College of Nutrition*. 2013;**32**(4):264-71.
60. Adebawo O, Salau B, Ezima E, et al. Fruits and vegetables moderate lipid cardiovascular risk factor in hypertensive patients. *Lipids in Health & Disease*. 2006;**5**:14.
61. Agarwal U, Mishra S, Barnard ND, et al. A plant-based diet reduces depression and anxiety and improves work productivity: The GEICO multicenter trial. *Diabetes*. 2013;**62**:A195.
62. Agarwal U, Mishra S, Xu J, Levin S, Gonzales J, Barnard ND. A multicenter randomized controlled trial of a nutrition intervention program in a multiethnic adult population in the corporate setting reduces depression and anxiety and improves quality of life: the GEICO study. *American journal of health promotion*. 2015;**29**(4):245-54.
63. Agren JJ, Tvrzicka E, Nenonen MT, Helve T, Hanninen O. Divergent changes in serum sterols during a strict uncooked vegan diet in patients with rheumatoid arthritis. *British Journal of Nutrition*. 2001;**85**(2):137-9.
64. Ahc M. Vegetarian Diet vs. Mediterranean Diet to Reduce Cardiovascular Risk. *Clinical Cardiology Alert*. 2018;**37**(6):N.PAG-N.PAG.
65. Allen AJ, Talreja DR, Buchanan HA, Wetmore J, Winegar D. The effects of plant-based, mediterranean, paleolithic, and dash diets on cardiovascular disease risk. *Cardiology (Switzerland)*. 2015;**131**:91.
66. Anderson JW. Plant fiber and blood pressure. *Annals of Internal Medicine*. 1983;**98**(5 Pt 2):842-6.
67. Anderson CAM. Dietary Patterns to Reduce Weight and Optimize Cardiovascular Health: Persuasive Evidence for Promoting Multiple, Healthful Approaches. *Circulation*. 2018;**137**(11):1114-6.
68. Andrews L. Vegetarian Diets & Heart Disease. *Communicating Food for Health*. 2017:8-.
69. Anonymous. Vegetarian diet cuts cholesterol. *Pharmaceutical Journal*. 2002;**269**(7227):799.
70. Anonymous. Special cholesterol-lowering diet can achieve same effects as statin therapy. *Pharmaceutical Journal*. 2003;**271**(7260):140.
71. Arntzenius AC. Diet, lipoproteins and the progression of coronary atherosclerosis. The Leiden Intervention Trial. *Drugs*. 1986;**31 Suppl 1**:61-5.
72. Barnard ND, Scherwitz LW, Ornish D. Adherence and acceptability of a low-fat, vegetarian diet among patients with cardiac disease. *Journal of Cardiopulmonary Rehabilitation*. 1992;**12**(6):423-31.
73. Barnard ND, Scialli AR, Bertron P, Hurlock D, Edmonds K, Talev L. Effectiveness of a low-fat vegetarian diet in altering serum lipids in healthy premenopausal women. *American Journal of Cardiology*. 2000;**85**(8):969-72.
74. Barnard ND, Scialli AR, Turner-McGrievy G, Lanou AJ. Acceptability of a low-fat vegan diet compares favorably to a step II diet in a randomized, controlled trial. *Journal of Cardiopulmonary Rehabilitation*. 2004;**24**(4):229-35.
75. Barnard ND, Scialli AR, Turner-McGrievy G, Lanou AJ, Glass J. The effects of a low-fat, plant-based dietary intervention on body weight, metabolism, and insulin sensitivity. *American Journal of Medicine*. 2005;**118**(9):991-7.
76. Barnard ND, Noble EP, Ritchie T, et al. D2 dopamine receptor Taq1A polymorphism, body weight, and dietary intake in type 2 diabetes. *Nutrition*. 2009;**25**(1):58-65.
77. Barnard ND, Rembert E, Freeman A, Bradshaw M, Holubkov R, Kahleova H. Blood Type Is Not Associated with Changes in Cardiometabolic Outcomes in Response to a Plant-Based Dietary Intervention. *Journal of the Academy of Nutrition & Dietetics*. 2020;**10**:10.
78. Beilin LJ. Vegetarian diets, alcohol consumption, and hypertension. *Annals of the New York Academy of Sciences*. 1993;**676**:83-91.
79. Bennett FC, Ingram DM. Diet and female sex hormone concentrations: an intervention study for the type of fat consumed. *American Journal of Clinical Nutrition*. 1990;**52**(5):808-12.
© 2023 Wang T et al. *JAMA Network Open.*

80. Bhardwaj S, Misra A, Gulati S, Anoop S, Kamal VK, Pandey RM. A randomized controlled trial to evaluate the effects of high Protein Complete (lActo) VEgetaRian (PACER) diet in non-diabetic obese Asian Indians in North India. *Heliyon*. 2017;**3**(12):e00472.
81. Brestrich M, Claus J, Blumchen G. Lactovegetarian diet: Influence on weight, lipids, fibrinogen and lipoprotein (a) of heart-patients in the course of their clinical rehabilitation. [German]. *Zeitschrift fur Kardiologie*. 1996;**85**(6):418-27.
82. Brom B. Integrative approach to management of heart disease is heart disease reversible? *South African Family Practice*. 2009;**51**(3):196-7.
83. Bunner AE, Gonzalez J, Agarwal U, Valente F, Barnard ND. Nutrition intervention for diabetic neuropathy. *Diabetes*. 2014;**63**:A578.
84. Burke LE, Hudson AG, Warziski MT, et al. Effects of a vegetarian diet and treatment preference on biochemical and dietary variables in overweight and obese adults: a randomized clinical trial. *American Journal of Clinical Nutrition*. 2007;**86**(3):588-96.
85. Campbell T. A plant-based diet and stroke. *Journal of Geriatric Cardiology*. 2017;**14**(5):321-6.
86. Campbell WW, Barton Jr ML, Cyr-Campbell D, et al. Effects of an omnivorous diet compared with a lactoovovegetarian diet on resistance-training-induced changes in body composition and skeletal muscle in older men. *American Journal of Clinical Nutrition*. 1999;**70**(6):1032-9.
87. Campbell WW, Tang M. Protein intake, weight loss, and bone mineral density in postmenopausal women. *Journals of Gerontology Series A-Biological Sciences & Medical Sciences*. 2010;**65**(10):1115-22.
88. Cesari F, Dinu M, Pagliai G, et al. Mediterranean, but not lacto-ovo-vegetarian, diet positively influence circulating progenitor cells for cardiovascular prevention: The CARDIVEG study. *Nutrition Metabolism & Cardiovascular Diseases*. 2019;**29**(6):604-10.
89. Chamorro R, Gonzalez MF, Aliaga R, et al. Diet, Plasma, Erythrocytes, and Spermatozoa Fatty Acid Composition Changes in Young Vegan Men. *Lipids*. 2020;**55**(6):639-48.
90. Crimarco A, Springfield S, Petlura C, et al. A randomized crossover trial on the effect of plant-based compared with animal-based meat on trimethylamine-N-oxide and cardiovascular disease risk factors in generally healthy adults: Study With Appetizing Plantfood-Meat Eating Alternative Trial (SWAP-MEAT). *American Journal of Clinical Nutrition*. 2020;**112**(5):1188-99.
91. Dewell A, Weidner G, Sumner MD, Chi CS, Ornish D. A very-low-fat vegan diet increases intake of protective dietary factors and decreases intake of pathogenic dietary factors. *Journal of the American Dietetic Association*. 2008;**108**(2):347-56.
92. Dinu M, Colombini B, Pagliai G, et al. Effects of a dietary intervention with Mediterranean and vegetarian diets on hormones that influence energy balance: results from the CARDIVEG study. *International Journal of Food Sciences & Nutrition*. 2020;**71**(3):362-9.
93. Dinu M, Pagliai G, Cesari F, et al. Effects of a 3-month dietary intervention with a lacto-ovo-vegetarian diet on vitamin B12 levels in a group of omnivores: results from the CARDIVEG (Cardiovascular Prevention with Vegetarian Diet) study. *British Journal of Nutrition*. 2019;**121**(7):756-62.
94. Dinu M, Pagliai G, Cesari F, et al. Effects of a 3-months' dietary intervention with lacto-ovo-vegetarian diet on vitamin B12 levels: Results of the CARDIVEG study. *Nutrition, Metabolism and Cardiovascular Diseases*. 2019;**29 (8)**:882-3.
95. Dinu M, Pagliai G, Colombini B, et al. Dietary intervention with vegetarian and mediterranean diets for cardiovascular prevention: Effects on hormones involved in the energy balance. *Nutrition, Metabolism and Cardiovascular Diseases*. 2019;**29 (8)**:881.
96. Dogra S, Getz A, Woolf K, et al. Long-term Dietary and Weight Changes Following a Short-term Dietary Intervention Study: 4-year Follow-up of the Evade Cad Trial. *Circulation Conference: American Heart Association Scientific Sessions, AHA*. 2020;**142**(SUPPL 3).
97. Elkan A-C, Sjöberg B, Kolsrud B, Ringertz B, Hafström I, Frostegård J. Gluten-free vegan diet induces decreased LDL and oxidized LDL levels and raised atheroprotective natural antibodies against phosphorylcholine in patients with rheumatoid arthritis: a randomized study. *Arthritis research & therapy*. 2008;**10**(2):R34-R.

© 2023 Wang T et al. *JAMA Network Open.*

98. Elkoustaf RA, Ismail M, Batiste C, et al. Diet and ischemic burden: A comparative analysis of a plant based regimen versus a mediterranean regimen in patients with coronary artery disease. *Journal of the American College of Cardiology*. 2017;**69 (11 Supplement 1)**:48.
99. Elkoustaf RA, Aldaas OM, Batiste CD, et al. Lifestyle Interventions and Carotid Plaque Burden: A Comparative Analysis of Two Lifestyle Intervention Programs in Patients with Coronary Artery Disease. *The Permanente journal*. 2019;**23**:18.196.
100. Fallah Noroozinejad E, Hosseinpour-Niazi S, Mirmiran P, Hedayati M, Delshad H. Effect of legumes intake on the oxidative stress markers in type 2 diabetes patients: A randomized cross-over study. [Persian]. *Iranian Journal of Endocrinology and Metabolism*. 2015;**16**(5).
101. Feifer C, Upadhyay P, Potter K. Lifestyle change for weight loss in the inner-city. *Californian Journal of Health Promotion*. 2003;**1**(3):7-13.
102. Fenton T, Gillis C. Plant-based diets do not prevent most chronic diseases. *Critical Reviews in Food Science & Nutrition*. 2019;**59**(7):1044-5.
103. Ferdowsian HR, Barnard ND, Hoover VJ, et al. A multicomponent intervention reduces body weight and cardiovascular risk at a GEICO corporate site. *American Journal of Health Promotion*. 2010;**24**(6):384-7.
104. Flynn MM, Reinert SE. Comparing an olive oil-enriched diet to a standard lower-fat diet for weight loss in breast cancer survivors: a pilot study. *Journal of Women's Health (15409996)*. 2010;**19**(6):1155-61.
105. Frassetto L, Pra M, Thomas B, Talreja D. The VA Beach Diet Study: A comparison of the effects of Plant-based, Mediterranean, Paleolithic, and DASH Diets on cardiovascular disease risk. *Journal of Evolution and Health*. 2019;**3**.
106. Gage A. Growing old in a circle of friends: Sangha and the lifestyle heart trial. *Topics in Geriatric Rehabilitation*. 2011;**27**(2):162-6.
107. Gardner CD, Kiazand A, Alhassan S, et al. Comparison of the Atkins, Zone, Ornish, and LEARN Diets for Change in Weight and Related Risk Factors Among Overweight Premenopausal WomenThe A TO Z Weight Loss Study: A Randomized Trial. *JAMA*. 2007;**297**(9):969-77.
108. Gorder DD, Dolecek TA, Coleman GG. Dietary intake in the Multiple Risk Factor Intervention Trial (MRFIT): Nutrient and food group changes over 6 years. *Journal of the American Dietetic Association*. 1986;**86**(6):744-51.
109. Gould KL, Ornish D, Kirkeeide R, et al. Improved stenosis geometry by quantitative coronary arteriography after vigorous risk factor modification. *American Journal of Cardiology*. 1992;**69**(9):845-53.
110. Gould KL, Ornish D, Scherwitz L, et al. Changes in myocardial perfusion abnormalities by positron emission tomography after long-term, intense risk factor modification. *Jama*. 1995;**274**(11):894-901.
111. Hakala P, Karvetti RL. Weight reduction on lactovegetarian and mixed diets. Changes in weight, nutrient intake, skinfold thicknesses and blood pressure. *European Journal of Clinical Nutrition*. 1989;**43**(6):421-30.
112. Harsha DW, Lin PH, Obarzanek E, Karanja NM, Moore TJ, Caballero B. Dietary Approaches to Stop Hypertension: A summary of study results. *Journal of the American Dietetic Association*. 1999;**99**(8 SUPPL.):S35-S9.
113. Harvey T, Smillie L, Ikin A, Farshid A. 753 The Effect of a Plant-Based Diet on Cardiovascular Risk Factors in Patients With Ischaemic Heart Disease. *Heart Lung and Circulation*. 2020;**29 (Supplement 2)**:S375.
114. Haub MD, Wells AM, Campbell WW. Beef and soy-based food supplements differentially affect serum lipoprotein-lipid profiles because of changes in carbohydrate intake and novel nutrient intake ratios in older men who resistive-train. *Metabolism: Clinical & Experimental*. 2005;**54**(6):769-74.
115. Hosseinpour-Niazi S, Mirmiran P, Hedayati M, Azizi F. Substitution of red meat with legumes in the therapeutic lifestyle change diet based on dietary advice improves cardiometabolic risk factors in overweight type 2 diabetes patients: a cross-over randomized clinical trial. *European Journal of Clinical Nutrition*. 2015;**69**(5):592-7.
116. Hunt JR, Matthys LA, Johnson LK. Zinc absorption, mineral balance, and blood lipids in women consuming controlled lactoovovegetarian and omnivorous diets for 8 wk. *American Journal of Clinical Nutrition*. 1998;**67**(3):421-30.

© 2023 Wang T et al. *JAMA Network Open.*

117. Jenkins DJA, Chiavaroli L, Wong JMW, et al. Adding monounsaturated fatty acids to a dietary portfolio of cholesterol-lowering foods in hypercholesterolemia. *CMAJ : Canadian Medical Association journal = journal de l'Association medicale canadienne*. 2010;**182**(18):1961-7.
118. Jibani MM, Bloodworth LL, Foden E, Griffiths KD, Galpin OP. Predominantly vegetarian diet in patients with incipient and early clinical diabetic nephropathy: effects on albumin excretion rate and nutritional status. *Diabetic Medicine*. 1991;**8**(10):949-53.
119. Kahleova H, Fleeman R, Hlozkova A, Holubkov R, Barnard ND. A plant-based diet in overweight individuals in a 16-week randomized clinical trial: metabolic benefits of plant protein. *Nutrition & Diabetes*. 2018;**8**(1):58.
120. Kahleova H, Hill M, Pelikanova T. Vegetarian vs. conventional diabetic diet - A 1-year follow-up. *Cor et Vasa*. 2014;**56**(2):e140-e4.
121. Kahleova H, Klementova M, Herynek V, et al. The Effect of a Vegetarian vs Conventional Hypocaloric Diabetic Diet on Thigh Adipose Tissue Distribution in Subjects with Type 2 Diabetes: A Randomized Study. *Journal of the American College of Nutrition*. 2017;**36**(5):364-9.
122. Kahleova H, Klemetova M, Herynek V, et al. The effect of a vegetarian vs. Conventional hypocaloric diabetic diet on thigh adipose tissue distribution in subjects with type 2 diabetes. *Diabetes*. 2017;**66 (Supplement 1)**:A202.
123. Kahleova H, Matoulek M, Hill M, Pelikanova T. Vegetarian diet increases resting energy expenditure more than conventional diet in subjects with type 2 diabetes after aerobic exercise. *Diabetes*. 2011;**60**:A217.
124. Kahleova H, Matoulek M, Kazdova L, Hill M, Pelikanova T. The effect of vegetarian diet on fatty acid composition of serum phopholipids and the association with insulin sensitivity and visceral fat in subjects with type 2 diabetes. *Diabetologia*. 2011;**54**:S359-S60.
125. Kahleova H, Matoulek M, Malinska H, Oliyarnik O, Kazdova L, Pelikanova T. Vegetarian diet improves plasma concentrations of adipokines and oxidative stress markers more than conventional diabetic diet in subjects with type 2 diabetes. *Diabetologia*. 2010;**53**:S380.
126. Kahleova H, Petersen KF, Shulman GI, et al. A dietary intervention to alter insulin sensitivity, intramyocellular and hepatocellular lipids, postprandial metabolism, and body weight: A 16-week randomised trial. *Diabetologia*. 2020;**63 (SUPPL 1)**:S16-S7.
127. Kahleova H, Rembert E, Alwarith J, et al. Effects of a Low-Fat Vegan Diet on Gut Microbiota in Overweight Individuals and Relationships with Body Weight, Body Composition, and Insulin Sensitivity. A Randomized Clinical Trial. *Nutrients*. 2020;**12**(10):24.
128. Kahleova H, Rembert E, Nowak A, Holubkov R, Barnard ND. Effect of a diet intervention on cardiometabolic outcomes: Does race matter? A randomized clinical trial. *Clinical Nutrition ESPEN*. 2021;**41**:126-8.
129. Kahleova H, Tonstad S, Rosmus J, et al. The effect of a vegetarian versus conventional hypocaloric diet on serum concentrations of persistent organic pollutants in patients with type 2 diabetes. *Nutrition Metabolism & Cardiovascular Diseases*. 2016;**26**(5):430-8.
130. Kahleova H, Tura A, Hill M, Holubkov R, Barnard ND. A Plant-Based Dietary Intervention Improves Beta-Cell Function and Insulin Resistance in Overweight Adults: A 16-Week Randomized Clinical Trial. *Nutrients*. 2018;**10**(2):09.
131. Kahleova H, Dort S, Barnard ND, Holubkov R. A Plant-Based High-Carbohydrate, Low-Fat Diet in Overweight Individuals in a 16-Week Randomized Clinical Trial: The Role of Carbohydrates. *Nutrients*. 2018;**10**(9):1302.
132. Kahleova H, Hlozkova A, Fleeman R, Fletcher K, Barnard ND, Holubkov R. Fat Quantity and Quality, as Part of a Low-Fat, Vegan Diet, Are Associated with Changes in Body Composition, Insulin Resistance, and Insulin Secretion. A 16-Week Randomized Controlled Trial. *Nutrients*. 2019;**11**(3):615-.
133. Kahleova H, McCann J, Alwarith J, et al. A plant-based diet in overweight adults in a 16-week randomized clinical trial: The role of dietary acid load. *Clinical Nutrition ESPEN*. 2021.
134. Kalinina OS, Shtraikher AP. [Changes of arterial pressure, pulse, circulation rate and electrocardiographic indices in hypertension following meat and vegetable diets]. *Terapevticheskii Arkhiv*. 1958;**30**(7):72-6.
© 2023 Wang T et al. *JAMA Network Open.*

135. Katcher HI, Ferdowsian HR, Hoover VJ, Cohen JL, Barnard ND. A worksite vegan nutrition program is well-accepted and improves health-related quality of life and work productivity. *Annals of Nutrition & Metabolism*. 2010;**56**(4):245-52.
136. Kestin M, Rouse IL, Correll RA, Nestel PJ. Cardiovascular disease risk factors in free-living men: comparison of two prudent diets, one based on lactoovovegetarianism and the other allowing lean meat. *American Journal of Clinical Nutrition*. 1989;**50**(2):280-7.
137. Kjeldsen-Kragh J, Borchgrevink CF, Laerum E, et al. Controlled trial of fasting and one-year vegetarian diet in rheumatoid arthritis. *The Lancet*. 1991;**338**(8772):899-902.
138. Kochan Z, Mironiuk K, Mickiewicz A, et al. N-3 Pufa-Enriched Semi-Vegetarian Diet Lowers Ldl-Cholesterol and Uric Acid Levels in Patients with Familial Hypercholesterolemia. *Clinical Nutrition*. 2019;**38 (Supplement 1)**:S233.
139. Koertge J, Weidner G, Elliott-Eller M, et al. Improvement in medical risk factors and quality of life in women and men with coronary artery disease in the Multicenter Lifestyle Demonstration Project. *American Journal of Cardiology*. 2003;**91**(11):1316-22.
140. Krishnamoorthy KM. Diet and coronary artery disease. *Indian Heart Journal*. 1999;**51**(3):268-74.
141. Lasserre N. Effectiveness of a dietotherapeutic intervention on the lipids profile and the nutritional status in breast cancer women. *Annals of nutrition & metabolism*. 2017;**71**(895).
142. Levin SM, Ferdowsian HR, Hoover VJ, Green AA, Barnard ND. A worksite programme significantly alters nutrient intakes. *Public Health Nutrition*. 2010;**13**(10):1629-35.
143. Mangels R. People with Type 2 Diabetes Benefit from Vegan and "Plant-Based" Diets. *Vegetarian Journal*. 2019;**38**(2):13-.
144. Marniemi J, Seppanen A, Hakala P. Long-term effects on lipid metabolism of weight reduction on lactovegetarian and mixed diet. *International Journal of Obesity*. 1990;**14**(2):113-25.
145. Masarei JR, Rouse IL, Lynch WJ, Robertson K, Vandongen R, Beilin LJ. Effects of a lacto-ovo vegetarian diet on serum concentrations of cholesterol, triglyceride, HDL-C, HDL2-C, HDL3-C, apoprotein-B, and Lp(a). *American Journal of Clinical Nutrition*. 1984;**40**(3):468-78.
146. Medkova IL, Ivanov AN, Mosiakina LI, Goncharov LF. Blood lipids and intensity of free radical oxidant processes in elderly patients with ischemic heart disease on antiatherogenic vegetarian diet. [Russian]. *Klinicheskaia meditsina*. 2000;**78**(1):21-4.
147. Medkova IL, Ivanov AN, Mosyakina LI, Biryukova LS. Clinicohemadynamic and biochemical the effect of patients with coronary heart disease use combinated lactoovovegetarian diets and simvastatin. [Russian]. *Voprosy Pitaniia*. 2006;**75**(5):49-52.
148. Medkova IL, Mosiakina LI, Biriukova LS. Estimation of action of lactoovovegetarian and vegan diets on blood level of atherogenic lipoproteins in healthy people. [Russian]. *Voprosy pitaniia*. 2002;**71**(4):17-9.
149. Medkova IL, Mosiakina LI, Pavlova VE, Zharkov AP, Bugaev VA, Koryshev VI. [Balanced vegetarian diet in combined rehabilitation of patients suffering from ischemic heart disease]. *Klinicheskaia Meditsina*. 1997;**75**(1):28-31.
150. Mishra S, Barnard ND, Xu J, Trap C. A plant-based diet reduces body weight and cardiovascular risk: The geico multicenter trial. *Diabetes*. 2012;**61**:A192.
151. Misquitta R, Ergas I, Edwards L, Rahbar J, Kushi L. Preliminary evaluation of the health achieved through lifestyle transformation (HALT) program, a kaiser permanente lifestyle intervention. *Journal of Alternative and Complementary Medicine*. 2020;**26 (11)**:A9.
152. Monica Dinu M, Pagliai G, Mangino A, et al. Comparison between Mediterranean and Vegetarian diets for cardiovascular prevention: The CARDIVEG study. *European Journal of Preventive Cardiology*. 2017;**24 (1 Supplement 1)**:S136.
153. Moore WJ, McGrievy ME, Turner-McGrievy GM. Dietary adherence and acceptability of five different diets, including vegan and vegetarian diets, for weight loss: The New DIETs study. *Eating Behaviors*. 2015;**19**:33-8.
154. Navas-Carretero S, Pérez-Granados AM, Schoppen S, Vaquero MP. An oily fish diet increases insulin sensitivity compared to a red meat diet in young iron-deficient women. *British Journal of Nutrition*. 2009;**102**(4):546-53.
© 2023 Wang T et al. *JAMA Network Open.*

155. Nichols A, Grosel J. Vegetarianism in the fight against CVD. *Clinical Advisor*. 2014;**17**(8):24-38.
156. Nicholson AS. Effect of a low-fat, unrefined, vegan diet on type 2 diabetes. *American journal of clinical nutrition*. 1999;**70**(35):624S-5S.
157. Nomura G. Non-pharmacological treatment of hypertension. [Japanese]. *Nippon rinsho*. 1989;**Japanese journal of clinical medicine. 47**(9):2074-8.
158. Pagliai G, Dinu M, Cesari F, et al. Randomized controlled dietary intervention trial comparing mediterranean and vegetarian diets for cardiovascular prevention: preliminary results. *European heart journal*. 2016;**37**(340).
159. Pagliai G, Dinu M, Mangino A, et al. Comparison between mediterranean and vegetarian diets for cardiovascular prevention: The cardiveg study. *Nutrition, Metabolism and Cardiovascular Diseases*. 2017;**27 (1)**:e30-e1.
160. Pagliai G, Russo E, Baldi S, et al. Impact of mediterranean vs vegetarian diets on gut microbiota and short chain fatty acids: The CARDIVEG study. *Nutrition, Metabolism and Cardiovascular Diseases*. 2019;**29 (8)**:879.
161. Pagliai G, Russo E, Niccolai E, et al. Influence of a 3-month low-calorie Mediterranean diet compared to the vegetarian diet on human gut microbiota and SCFA: the CARDIVEG Study. *European Journal of Nutrition*. 2020;**59**(5):2011-24.
162. Phillips KM, Stewart KK, Karanja NM, et al. Validation of diet composition for the dietary approaches to stop hypertension trial. *Journal of the American Dietetic Association*. 1999;**99**(8 SUPPL.):S60-S8.
163. Prescott SL, Jenner DA, Beilin LJ, Margetts BM, Vandongen R. Controlled study of the effects of dietary protein on blood pressure in normotensive humans. *Clinical & Experimental Pharmacology & Physiology*. 1987;**14**(3):159-62.
164. Prescott SL, Jenner DA, Beilin LJ, Margetts BM, Vandongen R. A randomized controlled trial of the effect on blood pressure of dietary non-meat protein versus meat protein in normotensive omnivores. *Clinical Science*. 1988;**74**(6):665-72.
165. Rajaram S, Sabate J. Health benefits of a vegetarian diet. *Nutrition*. 2000;**16**(7-8):531-3.
166. Rodriguez-Rodriguez E, Ortega RM, Lopez-Sobaler AM, et al. Restricted-energy diets rich in vegetables or cereals improve cardiovascular risk factors in overweight/obese women. *Nutrition Research*. 2007;**27**(6):313-20.
167. Rouse IL, Beilin LJ. Vegetarian diet and blood pressure. *Journal of Hypertension*. 1984;**2**(3):231-40.
168. Rouse IL, Beilin LJ, Mahoney DP, et al. Nutrient intake, blood pressure, serum and urinary prostaglandins and serum thromboxane B2 in a controlled trial with a lacto-ovo-vegetarian diet. *Journal of Hypertension*. 1986;**4**(2):241-50.
169. Rugulies R, Jager A, Benesch L, Siegrist J. Comprehensive lifestyle changes among patients with CHD and the integration of inpatient and outpatient cardiac rehabilitation: First results of a secondary prevention intervention trial. [German]. *Zeitschrift fur Gesundheitswissenschaften*. 1996;**4**(3):234-47.
170. Sanchez A, Chung SC, Mejia A, et al. Multiple lifestyle interventions reverses hypertension. *Cogent Medicine*. 2019;**6**(1).
171. Sanders TA, Ellis FR. Serum cholesterol and triglycerides concentrations in vegans. *Proceedings of the Nutrition Society*. 1977;**36**(1):43A.
172. Sarvesh Kumar J, Preetha S. Effect of aerobics exercise and dietary habits on blood pressure in hypertensives. *International Journal of Pharmaceutical Sciences Review and Research*. 2016;**39**(2):81-4.
173. Schmidt T, Wijga A, Von Zur Muhlen A, Brabant G, Wagner TOF. Changes in cardiovascular risk factors and hormones during a comprehensive residential three month kriya yoga training and vegetarian nutrition. *Acta Physiologica Scandinavica, Supplement*. 1997;**161**(640):158-62.
174. Sethna N, Magar NG. Cholesterol and phospholipids in aged groups. *Indian Journal of Medical Research*. 1960:225-30.
175. Singh RB, Rastogi SS, Niaz MA, Ghosh S, Singh R, Gupta S. Effect of fat-modified and fruit- and vegetable-enriched diets on blood lipids in the Indian Diet Heart Study. *American Journal of Cardiology*. 1992;**70**(9):869-74.

© 2023 Wang T et al. *JAMA Network Open.*

176. Sinha SD, Prasad RR, Sharma SN. Study of cardiovascular autonomic function in relation to the duration of menopause and vegetarian diet. *Indian Journal of Physiology and Pharmacology*. 2012;**1)**:122.
177. Soare A, Khazrai YM, Del Toro R, et al. The effect of the macrobiotic Ma-Pi 2 diet vs. the recommended diet in the management of type 2 diabetes: the randomized controlled MADIAB trial. *Nutrition & Metabolism*. 2014;**11**(1):39.
178. Spence JD, Tangney C. Lower risk of stroke with a vegetarian diet. *Neurology*. 2020;**94**(11):463-4.
179. Talreja A, Talreja S, Talreja DR. An investigation of plant-based, mediterranean, paleolithic, and dash diets study (the Va Beach Diet Study). *Catheterization and Cardiovascular Interventions*. 2021;**97**(SUPPL 1):S109.
180. Talreja DR, Talreja S, Talreja A. THE VA-BEACH DIET STUDY: A COMPARISON OF PLANT-BASED, MEDITERRANEAN, PALEOLITHIC AND DASH DIETS ON LIPID AND BIOMETRIC PARAMETERS. *Journal of the American College of Cardiology*. 2021;**77**(18 Supplement 1):1478.
181. Talreja S, Talreja D. Prospective Comparison of Lipid Responses in Plant-based, Mediterranean, Paleolithic an Dash Diets. *Circulation Conference: American Heart Association Scientific Sessions, AHA*. 2020;**142**(SUPPL 3).
182. Thedford K, Raj S. A vegetarian diet for weight management. *Journal of the American Dietetic Association*. 2011;**111**(6):816-8.
183. Thomson CA, Rock CL, Giuliano AR, et al. Longitudinal changes in body weight and body composition among women previously treated for breast cancer consuming a high-vegetable, fruit and fiber, low-fat diet. *European Journal of Nutrition*. 2005;**44**(1):18-25.
184. Tjiong BK, Groen J, Kamminga CE, Willebrands AF. Influence of diet and of some other factors on the blood cholesterol. [Dutch]. *Nederlands Tijdschrift voor Geneeskunde*. 1952;**96**(8):472-4.
185. Toobert DJ, Glasgow RE, Nettekoven LA, Brown JE. Behavioral and psychosocial effects of intensive lifestyle management for women with coronary heart disease. *Patient Education & Counseling*. 1998;**35**(3):177-88.
186. Turner-McGrievy GM, Barnard ND, Scialli AR. A two-year randomized weight loss trial comparing a vegan diet to a more moderate low-fat diet. *Obesity*. 2007;**15**(9):2276-81.
187. Turner-McGrievy GM, Barnard ND, Scialli AR, Lanou AJ. Effects of a low-fat vegan diet and a Step II diet on macro- and micronutrient intakes in overweight postmenopausal women. *Nutrition*. 2004;**20**(9):738-46.
188. Turner-McGrievy GM, Davidson CR, Wingard EE, Billings DL. Low glycemic index vegan or low-calorie weight loss diets for women with polycystic ovary syndrome: a randomized controlled feasibility study. *Nutrition Research*. 2014;**34**(6):552-8.
189. Turner-McGrievy GM, Davidson CR, Wingard EE, Wilcox S, Frongillo EA. Comparative effectiveness of plant-based diets for weight loss: a randomized controlled trial of five different diets. *Nutrition*. 2015;**31**(2):350-8.
190. Turner-McGrievy GM, Leach AM, Wilcox S, Frongillo EA. Differences in Environmental Impact and Food Expenditures of Four Different Plant-based Diets and an Omnivorous Diet: Results of a Randomized, Controlled Intervention. *Journal of Hunger and Environmental Nutrition*. 2016;**11**(3):382-95.
191. Turner-McGrievy GM, Wirth MD, Shivappa N, et al. Randomization to plant-based dietary approaches leads to larger short-term improvements in Dietary Inflammatory Index scores and macronutrient intake compared with diets that contain meat. *Nutrition Research*. 2015;**35**(2):97-106.
192. Veleba J, Matoulek M, Hill M, Pelikanova T, Kahleova H. "A Vegetarian vs. Conventional Hypocaloric Diet: The Effect on Physical Fitness in Response to Aerobic Exercise in Patients with Type 2 Diabetes." A Parallel Randomized Study. *Nutrients*. 2016;**8**(11):26.
193. Whitten C. Vegetarian diets and ischemic heart disease. *Topics in Clinical Nutrition*. 1995;**10**(2):27-33.
194. Wirths W, Rehage-Thones C, Bonnhoff N, Passelewitz U. [Effect of an egg-milk-vegetarian diet on nutritional and blood status. II. Findings of a study on circulation, blood status; discussion]. *Zeitschrift fur Ernahrungswissenschaft*. 1988;**27**(2):84-100.
195. Yarnell SR. Cardioprotective diet after recent myocardial infarction. *Annals of Internal Medicine*. 1992;**117**(SUPPL. 3):76.

© 2023 Wang T et al. *JAMA Network Open.*

196. Zeis BM, Muller-Nothmann SD. Health effect of a fruit and vegetable rich diet. Part 1: Cardiovascular diseases. [German]. *Schweizerische Zeitschrift fur GanzheitsMedizin*. 2008;**20**(3):144-8.
197. Zemel MB. Dietary pattern and hypertension: The DASH study. *Nutrition Reviews*. 1997;**55**(8):303-5.
198. Vegan diet helps treat type 2 diabetes. *Environmental Nutrition*. 2006;**29**(10):1-.
199. Plant-based diets can lower CV mortality risk. *Nephrology News & Issues*. 2019;**33**(6):34-.
200. Impact of a 12-month Inflammation Management Intervention on the Dietary Inflammatory Index, inflammation, and lipids. *Clinical nutrition ESPEN*. 2019;**30**:42-51.
201. Effects of the lifestyle modification program to reduce serum lipoprotein(a) and other cardiovascular risk factors in Korean college women. *Gazzetta medica italiana archivio per LE scienze mediche*. 2018;**177**(9):468-74.

© 2023 Wang T et al. *JAMA Network Open.*
