# Supplementary material for: Vegetarian Dietary Patterns and Cardiometabolic Risk in People With or at High Risk of Cardiovascular Disease: A Systematic Review and Meta-analysis
Source: JAMA Netw Open. 2023 Jul 25;6(7):e2325658. doi: 10.1001/jamanetworkopen.2023.25658 (PMC10369207; doi:10.1001/jamanetworkopen.2023.25658)
Supplement: Supplement 3. — Data Sharing Statement [file jamanetwopen-e2325658-s003.pdf]

Data Sharing Statement

Wang. Vegetarian Dietary Patterns and Cardiometabolic Risk in People With or at High Risk of Cardiovascular Disease. *JAMA Netw Open*. Published July 25, 2023.
doi:10.1001/jamanetworkopen.2023.25658

Data
Data available: Yes
Data types: Deidentified participant data
How to access data: Most extracted data and study materials are available from previously published research. The authors confirm that all data underlying the findings are fully available without restriction. All data and R script are available in the supplementary material.
When available: With publication

Supporting Documents
Document types: Statistical/analytic code
How to access documents: With publication
When available: With publication

Additional Information
Who can access the data: All readers.
Types of analyses: For any purpose.
Mechanisms of data availability: With publication.
